# Ultrathin Ferroelectric Films: Growth, Characterization, Physics and Applications

**DOI:** 10.3390/ma7096377

**Published:** 2014-09-11

**Authors:** Ying Wang, Weijin Chen, Biao Wang, Yue Zheng

**Affiliations:** 1 State Key Laboratory of Optoelectronic Materials and Technologies, School of Physics and Engineering, Sun Yat-sen University, Guangzhou 510275, China; E-Mail: wangyn96@mail2.sysu.edu.cn; 2 Micro & Nano Physics and Mechanics Research Laboratory, School of Physics and Engineering, Sun Yat-sen University, Guangzhou 510275, China; E-Mail: chenwjin@mail2.sysu.edu.cn

**Keywords:** ultrathin ferroelectric films, growth methods, characterization techniques, phenomena and properties, applications

## Abstract

Ultrathin ferroelectric films are of increasing interests these years, owing to the need of device miniaturization and their wide spectrum of appealing properties. Recent advanced deposition methods and characterization techniques have largely broadened the scope of experimental researches of ultrathin ferroelectric films, pushing intensive property study and promising device applications. This review aims to cover state-of-the-art experimental works of ultrathin ferroelectric films, with a comprehensive survey of growth methods, characterization techniques, important phenomena and properties, as well as device applications. The strongest emphasis is on those aspects intimately related to the unique phenomena and physics of ultrathin ferroelectric films. Prospects and challenges of this field also have been highlighted.

## 1. Introduction

Ferroelectrics are defined as polar materials that possess at least two equilibrium orientations of the spontaneous polarization vector in absence of an external electric field, and can be switched between those orientations by an electric field [1]. Its investigation can date back to 1920s when Valasek made researches on Rochelle salt [2]. Typically, ferroelectric materials undergo a structural phase transition at Curie point (*T_c_*), transforming from a nonpolar high-temperature paraelectric phase into a polar low-temperature ferroelectric phase accompanied with a lowering of symmetry.

A fundamental issue in ferroelectrics is the scaling of ferroelectric properties with size, namely ferroelectric size effects. Among various ferroelectric systems, thin film ferroelectrics have been the objectives of great interests for decades; a very large number of works has been established. Driven by the trend of device miniaturization and the fast developments achieved in film deposition methods and characterization techniques, ultrathin ferroelectric films (typically, with thickness smaller than 100 nm) have been successfully grown. Owing to their striking properties and application potential, they have drawn increasingly interests and have become the most popular research branch in the field of ferroelectrics. Recently, a variety of works both theoretical and experimental have been carried out on ultrathin ferroelectric films with remarkable progresses being made in many aspects, including film deposition, characterization, property research, and device design, *etc*. Although there have been some reviews on the topic of ferroelectric thin films, an up-to-date review specially focusing on ultrathin ferroelectric films and the recent progresses in this field is in demand.

Based on a comprehensive survey of excellent experimental works on ultrathin ferroelectric films, we set the target of this review paper as providing an extensive insight on state-of-the-art researches in the field. In the first part, the common deposition methods of ferroelectric ultrathin films have been introduced with thin film growth physics, also with special attention to the choice of substrates and the growth of heterostructures. For a deep understanding and for investigation of the behaviors of ultrathin films with thickness at nanometer magnitude, it is necessary to establish the analysis both at the level of macroscale and nanoscale. Thus, characterization methods for ultrathin ferroelectric films have been summarized in the second part, focusing on film property characterization, particularly on those unique properties in ultrathin ferroelectric films such as nanoscale domain structure and tunneling property. It will be emphasized that when the film thickness shrinks to a great extent, many intriguing phenomena can be observed and controlled. In the next part, significant developments of researches on important phenomena and properties in ultrathin ferroelectric films are thoroughly reviewed and discussed, aiming at providing readers snapshots on physics in ultrathin ferroelectric films. Associated with the fast development of integrated circuit technology, the advantages to be gained from the availability of ferroelectric thin films have been widely appreciated and significant efforts have been directed towards researches on exploitation of ferroelectric thin films in devices and multifunctional integrated MEMS [3]. In the final part of this review, an overview of the current state of ferroelectric thin film devices is introduced followed by identification and discussion of the key physics issues that determine device performance. In particular, the promising applications of ultrathin ferroelectric films, such as newly nonvolatile memory devices based on ferroelectric tunnel junctions, are highlighted with the prospects and current challenges being pointed out.

## 2. Growth of Ferroelectric Thin Films

We notice that great advances have been achieved in growth of ferroelectric thin film structures during the past decades. Particularly, due to the development of epitaxial growth methods, it is now possible to prepare high quality and ultrathin ferroelectric films that are single crystal and defect free. Compared with the synthesis of thin films made of simple substances (e.g., Si) or binary compounds (e.g., GaAs and ZnO), synthesis of ultrathin ferroelectric films requires more sophisticated and special equipment, due to their complicated chemical constituents and properties. The wide variety of growth techniques of ferroelectric thin films include molecular beam epitaxy (MBE), vacuum evaporation (VE), sputtering method (SM), pulsed laser deposition (PLD), chemical solution deposition (CSD), and chemical vapor deposition (CVD), atomic layer deposition (ALD) *etc.*, and they can be generally divided into two categories, *i.e.*, physical ones and chemical ones. Each method has its own strength and weakness. Thus the growth method has to be carefully selected to obtain a certain film with desired properties. In this section, growth methods of ferroelectric thin films will be discussed and some comparisons will be made among them, which should be relevant for readers who want information of thin film growth physics and suggestions on choice of growth methods.

### 2.1. Thin Film Growth Physics

At the primary stage of thin film growth, which is the so-called nucleation stage, abundant vapor atoms or molecules condense and undergo surface diffusion and migration under the drive of both their self-energy and substrate thermal energy, then move to a stable position on the substrate [4,5]. Subsequently the nucleus ceaselessly incorporates surrounding atoms or molecules and gradually grows to a bigger size, finally resulting in film formation. The film nucleation and forming process have been well observed through many techniques [6,7,8], such as transmission electron microscopy (TEM) and scanning electron microscopy (SEM), scanning probe microscopy (SPM) and field-ion microscopy (FIM), *etc*. 

**Figure 1:**
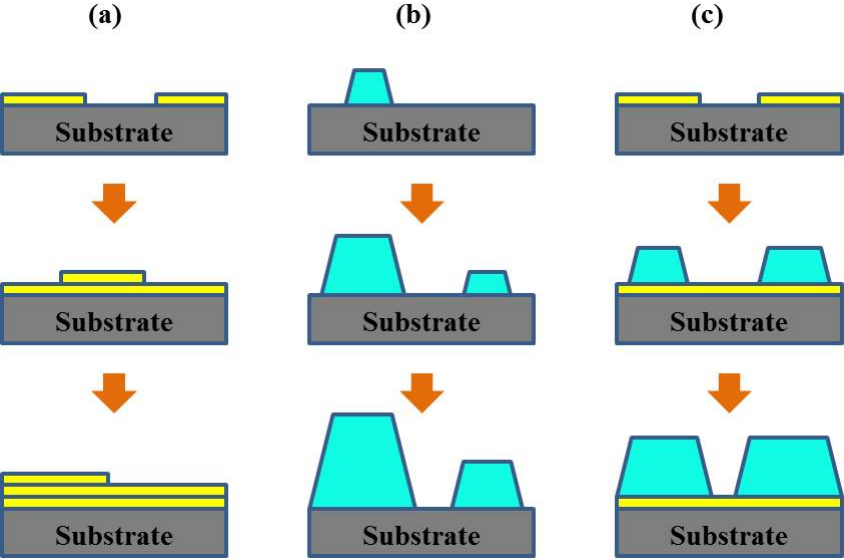
Schematic diagram of three basic growth modes: (**a**) layer-by-layer growth mode; (**b**) island growth mode; and (**c**) Stranski–Krastanov mode.

**Figure 2:**
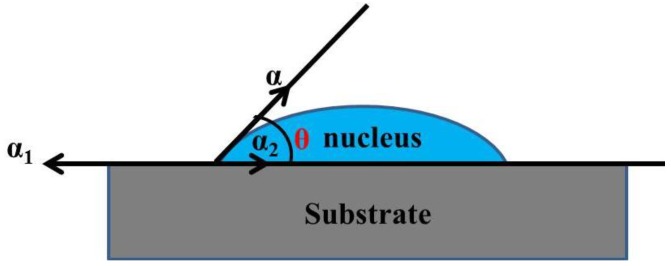
Spherical cap-shaped nucleus on surface: θ refers to the wetting angle (contact angle); α refers to surface tension of spherical cap-shaped nucleus; α_1_ refers to surface tension of surface; α_2_ refers to α_1_ refers to interfacial tension between nucleus and surface. The equilibrium equation of surface tension and interfacial tension is: αcosθ = α_1_ − α_2_.

Thin film formation on clean crystal substrates can be classified into three basic growth mode, including: (1) layer-by-layer growth mode (Frank–Van der Merwe mode); (2) island growth mode (Volmer–Weber mode); (3) Stranski–Krastanov mode, which are illustrated in Figure 1. When the wetting angle θ (see Figure 2) is approximately zero, the interaction between atoms or molecules is smaller than their bonding to the substrate. The smallest nucleuses will extent on the substrate in two dimensions, leading to the thin film growth mode of layer-by-layer (Figure 1a). It is noteworthy that the bonding effect in each layer tends to be weaker than its precious layer. This growth mode usually happens when the substrate and film are homogeneous materials or some particular dissimilar materials such as the epitaxial growth of semiconductor and oxide materials. When the wetting angle θ is greater than zero, the bonding between atoms or molecules is larger than that to the substrate, causing the atoms or molecules bonding strongly to each other and growing into many three-dimensional nucleus islands (see Figure 1b). For this island growth mode, polycrystalline thin films with rough surface are usually obtained as the continual growth of the islands. Island growth mode often happens when the substrate and film are heterogeneous. Stranski–Krastanov mode lies between the two above mentioned growth modes, *i.e.*, thin films firstly grow two-dimensionally in layer-by-layer mode, and then grow three-dimensionally in island mode (see Figure 1c), which happens in case of the generated stress impact after two-dimensional growth. The most attractive and popular film growth way is epitaxial growth, which refers to the formation of an extended single-crystal overlayer on a crystalline substrate, achieved through layer-by-layer growth mode. Introductions in details for epitaxial growth can be found in somewhere else [5,9]. 

### 2.2. The Choice of Substrates

Ferroelectric thin films are always deposited on substrates. A proper choice of substrates is important for growth of ferroelectric thin films. Effects such as substrate misfit strain can affect the film grow process and film properties, giving rise to the crucial points resting on the choice of appropriate substrates and methods to prepare highly chemically and structurally matched substrate surfaces for epitaxial growth. Perovskite epitaxial ferroelectric films have been successfully prepared with a variety of new perovskite and perovskite-related substrates [10,11], such as SrTiO_3_ [12], YAlO_3_ [13], LaAlO_3_ [14], LaGaO_3_ [15], LaSrAlO_4_ [16], LaSrGaO_4_ [17], NdGaO_3_ [18], KTaO_3_ [19], (LaAlO_3_)_0.29_-(Sr_1/2_Al_1/2_TaO_3_)_0.71_ (LSAT) [20] and ReScO_3_ [21,22], *etc*. Some commercially perovskite and perovskite-related substrates, and the pseudotetragonal or pseudocubic *a*-axis lattice constants of some frequently-used ferroelectric perovskites have been listed in Table 1, providing an intuitive reference for substrates select in film fabrication of this category [23]. Due to the fact that most of the commercially available perovskite substrates typically have lattice constants in the 3.8–3.9 Å range, it is obvious that the lattice constants of the commercially engaged perovskite substrates are generally smaller than those of the listed ferroelectric materials [23]. In atomic-scale epitaxy, substrates with defects–free surfaces of specific chemical termination are necessary. Some substrates of this kind have been produced, for instance, TiO_2_-terminated (100) SrTiO_3_ substrates and SrO-terminated (100) SrTiO_3_ substrates [24,25,26], controlled-termination substrates of NdGaO_3_ and KTaO_3_ [27,28].

**Table 1:** Commercially involved perovskite and perovskite-related substrates and the pseudotetragonal or pseudocubic a-axis lattice constants of some frequently-used ferroelectric perovskites. Substrates and thin films which are in the same lines possess similar lattice constants. In one vertical line, lattice constants of thin films or substrates are gradually increasing from top to bottom [23].

Lattice Constants (Å)	Perovskite-Related Substrates	Ferroelectric Thin Films	
**3.70–3.80**	YAlO_3_	–	
	LaSrAlO_4_		
	LaAlO_3_		
**3.80–3.90**	LaSrGaO_4_	Bi_4_Ti_3_O_12_	
	NdGaO_3_	–	
	LSAT	–	
	LaGaO_3_	–	
**3.90–4.00**	SrTiO_3_	SrBi_2_Ta_2_O_9_	(Ba,Sr)TiO_3_
	DyScO_3_	BiMnO_3_	
	GdScO_3_	BiFeO_3_	
	SmScO_3_	PbTiO_3_	
	KTaO_3_		
**4.00–4.10**	NdScO_3_	BaTiO_3_	Pb(Zr,Ti)O_3_
	–	PMN-PT	
**4.10–4.20**	–	Pb(Zr,Ti)O_3_	

Recent advances in mechanics and material science provide routes to integrated circuits that can offer the electrical properties of conventional rigid wafer-based technologies and with the ability to be deformed arbitrarily (e.g., stretched and twisted) by means of flexible substrates [29]. Flexible substrates, which are usually some plastic or elastomeric substrates, have extended the classes of substrates and attracted much attention for their applications in high-performance flexible electronics [29,30,31]. There are also significant efforts being devoted to transfer (stamp and print by using polydimethylsiloxane (PDMS) stamps or soluble glues) perovskite thin films or nanoribbons onto flexible substrates for the purpose of utilizing the high inherent piezo-properties of ferroelectric materials, or achieving stretchable properties without any loss in ferroelectric/piezoelectric properties [32,33,34]. For instance, PZT, BTO, and STO thin films, originally deposited on rigid substrates, have been successfully transferred onto flexible substrates by removing the sacrificial layers such as SiO_2_, MgO and TiO_2_, with the deformation mechanics and material properties being studied [32,33,34,35].

In spite of the widely used PDMS substrates, many flexible substrates have been employed into multifunctional flexible circuits based on ferroelectric thin films both organic (like P(VdF-TrFE) [36]) and inorganic. Some typical examples include polyethylene naphthalate (PEN) [35], polyethylene terephthalate (PET) [37], polyimide (PI) substrates [38], flexible aluminum substrates [36], and thin glass substrates [39] *etc*. However, the limited processing temperature ranges (mostly less than 300 °C) have restrained applications of those plastic substrates in extreme situations such as crystallization process of inorganic ferroelectrics at high annealing temperature, which may result in sophisticated transferring process. More recently, to solve this problem and simplify experiment process, polycrystalline metal sheet (e.g., polycrystalline Hastelloy tapes) has been proposed to be a promising candidate as the flexible substrate for growth of high performance multifunctional films to meet specific requirements [40]. In general, a buffer layer is usually needed to effectively connect flexible substrates with ferroelectric thin films [40]. It is also noteworthy that significant efforts have been devoted to low-temperature fabrication of promising inorganic ferroelectric thin films (e.g., PZT) on commonly used plastic substrates, among which activated-solution method is an effective one [41]. On the basis of these flexible substrates, ultrathin ferroelectric films can be further studied, with some piezo-related properties such as the specially-focused flexoelectric properties (as will be discussed in Section 4.2) being intensively researched. Moreover, the application scope of ultrathin ferroelectric films can be largely broadened by the use of flexible substrates, with ferroelectric thin-film-based devices such as nanogenerators, sensors and memories systems presenting stretchable properties and working efficiently with proper flexible substrates [32,33].

### 2.3. Evaporation Methods

**Figure 3:**
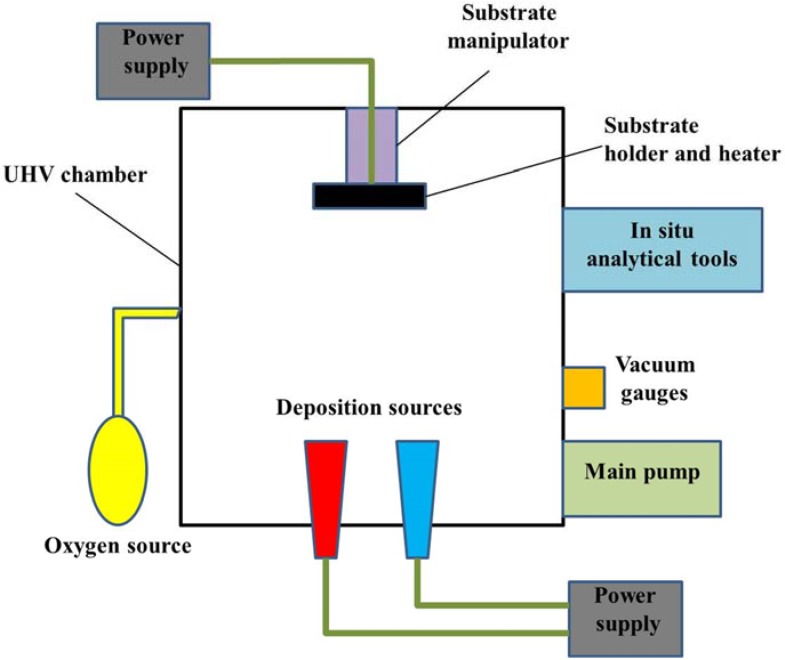
Schematic diagram of a basic physical vapor deposition (PVD) system [11].

There are a large variety of physical vapor deposition (PVD) methods widely used in thin film growth, mainly including vacuum evaporation (VE), molecular beam epitaxy (MBE), and pulsed laser deposition (PLD). For this kind of methods, the film growth is dominated by a physical evaporation process. Thermal energy is provided from a power supply unit to heat the atoms of a liquid or solid source to reach evaporating point. The vaporized atoms travel a distance (usually in a vacuum chamber) and deposit onto the heated substrate. Thin film is formed after a continuous evaporating process. According to different methods, the power supply unit may be a heating wire, electron beam, molecule beam or pulsed laser, *etc*. Note that some evaporation deposition methods also engage in chemical reactions between deposition sources, so-called activated reactive evaporation. The basic components of a modern PVD system are shown in Figure 3 [11].

Ferroelectric thin films are usually deposited through the activated reactive evaporation with reactions between sources and oxygen gas happen on the substrate. For example, for a typical perovskite ferroelectric, ABO_3_, thin film can be prepared by evaporating sources of A and B to react with introduced oxygen gas [42]. The essential reaction equation is:

A + B + 3/2O_2_ → ABO_3_ (1)

Technological problems in controlling film stoichiometry are often encountered in reactive evaporation method [43], which could be receded through individual evaporation of A and B sources to form multilayers on substrate with subsequent reactions in oxygen gas during the following thermal treatment [44].

Several factors have vital influence on the film growth speed. In general, atom/molecule mass of source, melting/boiling points of source, pressure in vacuum chamber, as well as evaporation temperature will determine the film growth speed. The distance between sources and substrate also influence the growth speed. According to kinetic theory, the number of molecules which change from gas phase to solid or liquid phase at unit time on unit area is represented by d*Z*. There is an equation as follows:

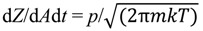
(2)


Here, *m* refers to molecule mass; *p* refers to the pressure; and *T* refers to temperature. When an equilibrium state is reached, the molecule numbers evaporated from source to gas is equal to those freezing back to source. At this moment, d*Z* is almost equivalently to the number of molecules evaporated out of source. Thus, the main affecting factors during evaporation process can be seen from the equation above.

#### 2.3.1. Vacuum Evaporation

The simplest PVD method for thin film growth is vacuum “direct” evaporation, where a deposition source is heated by a resistance heating wire or electron-beam and evaporated onto substrate in a high/ultrahigh vacuum. No chemical reactions happen during the whole deposition process, giving rise to the stoichiometry of prepared film similar to that of deposition source. The “direct” evaporation method is less utilized in growth of ferroelectric thin films than metal thin films. Another VE method is “indirect” evaporation (*i.e.*, multi-sources reactive evaporation), by which ferroelectric thin films are obtained through the reactions between sources and oxygen gas happened on the substrate. Through this method, a lot of ferroelectric thin films, e.g., PbTiO_3_ and BaTiO_3_ thin films have been obtained [44,45,46].

The main superiorities of VE are as follows. Firstly, film thickness is controllable in a wide range from several nanometers to hundreds of nanometers. Secondly, uniform film growth can be achieved and optimized through continuous source feeding with flash evaporation. Thirdly, the compatibility between thin film and substrate is good, thus there is little limit on choice of substrate. Finally, none complicated reactions are engaged in the whole procedure, resulting in easy operation of the evaporating equipment. However, there are some drawbacks of VE. Firstly, source materials with low vapor pressure are refractory, making them difficult to be evaporated and deposited onto substrate. Secondly, some source materials may react with source container, resulting in impurity of the thin film. Thirdly, non-stoichiometric films are likely to be found in multi-sources reactive evaporation. The last, evaporation happens in the whole vacuum chamber and causes large waste of sources.

#### 2.3.2. Pulsed Laser Deposition

The research of pulse laser deposition (PLD) can be dated back to the 1960s with the invention of high-power pulsed laser sources. In late 1980s, PLD was reinvented after the discovery of high-temperature superconductors. Now PLD has become an important material growth method capable of a wide range of materials and structures from single atomic layers to quasi-bulk crystalline materials [47,48,49]. Importantly, PLD provides the possibility of growing ultrathin epitaxial films, leading to enormous influence on modern film research.

In typical PLD equipment, when a focused laser pulse projects on a solid target, the target ablation will happen with the ablated materials transferring in a preferential direction along with the surface normal of the target, resulting in the formation of the plume (a feather-like luminophor). The ablated materials subsequently deposit onto the substrate and grow into a film. The deposition process can be generally divided into three stages, including laser pulses militating with target, ablated materials transferring in the chamber, and film growth on the substrate. PLD technique has been successfully applied to the growth of ultrathin ferroelectric films, such as Pb(Zr,Ti)O_3_ (PZT) [50,51], BiFeO_3_ (BFO) [52,53], BaTiO_3_/SrTiO_3_(BTO/STO) superlattices [54], Sr_x_Bi_(3−x)_Ta_2_O_9_ (SBT) [55,56], Ba_x_Sr_(1−x)_TiO_3_ (BST) [57], some complex structural films [58], and so on. The obtained films through PLD method are often epitaxial growth.

It is verified by a large amount of experiments that all elements in the solid target are evaporated simultaneously due to the fast heating in a small volume of the target surface under intense laser pulses. Therefore the most important advantage of PLD is that film stoichiometry is expected almost the same as the target, which makes PLD particular suitable for synthesis of films with sophisticated chemical constituents like ferroelectric films. In addition, targets for PLD can be obtained through standard powder ceramic techniques by simple precursors-mixing process, which is easier than most film-growth methods involving target. Since PLD is not element specific, it is easy to switch from one composition to another by simply targets changing [11]. Therefore different targets can be simultaneously settled in the same PLD vacuum chamber, which offers an opportunity for the growth of high quality ferroelectric heterostructures and superlattices. Furthermore, the working environment is flexible with the ability to use a suitable background gas in the deposition chamber, which, for example, can singly introduce oxygen gas at an appropriate ‘high’ pressure and is better for the formation of ferroelectric oxide thin films than other techniques (for example, MBE can only work under high-vacuum conditions, and sputtering requires the presence of other gases, such as argon) [11]. Moreover, reactive deposition in different ambience can be realized in a PLD system. With low-pressure gases like O_2_, O_3_, NO_2_, N_2_O or H_2_O, reactive deposition makes synthesis of complex high-quality thin film possible, which is improbable in previous synthesis technology.

Meanwhile, there are some disadvantages in PLD method. The film surface may have granuliform protuberance derived from solidification of subtle liquid drops on the surface, which causes non-uniformity and may sharply pull down the film quality. Moreover, it is difficult to prepare large-scale thin films by PLD method on account of the orientation movement of ablated materials. Attempts have been done to solve the problems. For instance, electric field bias has been employed to increase c-axis orientation of ferroelectric thin film [59], and shadow mask technique has been created to decrease surface roughness of thin films [60].

#### 2.3.3. Molecular Beam Epitaxy

Molecular beam epitaxy (MBE) is a high-precision crystal growth method developed in the late 1960s [61]. It is a vacuum evaporation technique that builds the crystal structure of a thin film with atomic-layer precision through flux control of thermally evaporated beams of constituent elements in an ultra-high vacuum (10^−8^ Pa). In typical MBE system, the beams are directed at a crystalline substrate to form the desirable film through reaction and crystallization [11]. Beams of metal atoms can be created using either radiatively heated sources or from electron beam evaporators [62,63]. RHEED (reflection high-energy electron diffraction) is usually applied in a MBE system to characterize atomic surface structure and morphology information of the sample during deposition. A predetermined crystal structure can be created by adjusting the sequence of material providing and evaporating, and the obtained crystal structures are always single crystal due to the growth mechanism of MBE. When the surface structure and chemistry do not match with the sequence of the source materials, the film grower will rely on electron-spectroscopic techniques that are inherently surface sensitive and that work well in the vacuum environment of MBE to provide feedback to the growth process, which will make MBE both scientifically and technologically successful [11]. Fruitful research developments in MBE technique for ultrathin ferroelectric film deposition have been got during its phylogeny, especially for perovskite oxides [64], such as ferroelectric YMnO_3_ [65], BTO [66,67,68], STO [69,70], PZT [71], and some superlattices [72,73], *etc*.

MBE techniques have been involved in intensive study in the past [62,74], the main characteristics are sum up and listed below. The first is that low growth rate of almost one monolayer (lattice plane) per second with in-situ control of crystal growth at the atomic level, which will not only give access to deposition of ultrathin film but also facilitate precisely control of film thickness. The second is low temperature deposition, which will reduce the possibility of lattice mismatch derive from thermal expansion and thermal diffusion pollution of substrate impurities. The third is precise control of surface composition and morphology, resulting in smooth growth surface with steps of atomic height and large flat terraces. The next is abrupt variation of chemical composition at interfaces, providing the possibility of superlattice growth. The last should be the film-doping procedure, which can be adjusted at any moment during epitaxial procedure by adjusting source material as required.

Although the quality of materials created by MBE is very good, widespread usage of this technique is limited due to a number of factors, such as high prices of the system, component, and source materials, the difficulty in guarantee of UHV background pressures, and the limits of source materials. These major stumbling blocks in the pursuit of science will be overcome through the combining of cutting-edge thin film growth techniques together with a wide-range of materials characterization methods [63].

It is worthy to mention that some optimized MBE-related systems for ultrathin film deposition have blossomed in recent years, among which the typically example is laser-MBE (L-MBE). L-MBE is a new film growth technique developed in recent years, which successfully possesses not only the excellent properties *in situ* detection and ultra-high vacuum of traditional MBE, but also the superiority in accurate component controlling and wide application range of PLD. Thus observation and regulation at atomic level will be realized with the expected monoatomic layer epitaxy in ferroelectric films becoming possible. It can be predicted that L-MBE technique has extreme potential to promote techniques for ultrathin ferroelectric film formation into a new promising level.

### 2.4. Sputtering Method

**Figure 4:**
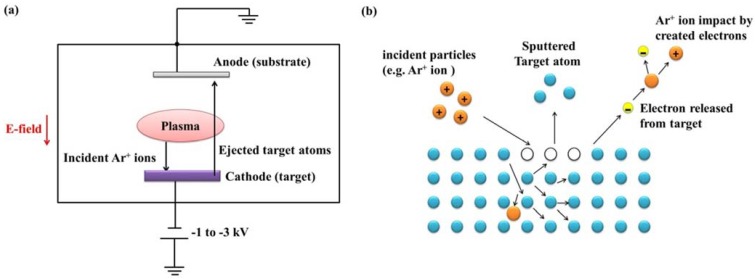
(**a**) Schematic diagram of a basic sputtering system [11]; (**b**) schematic sputtering mechanism.

Sputtering method is a widely used physical vapor deposition method, based on a mechanism where atoms are dislodged from the target surface by an incoming flux of highly energetic particles [75]. In this process, secondary electrons emitted from the target surface are crucial to the plasma maintaining. Figure 4 schematically shows a basic sputtering system and the sputtering mechanism. This technique, in particular geometries or with specific deposition parameters, shares the main feature with PLD of being capable of stoichiometrically transferring the target composition to the grown film, which makes it suitable to grow complex compound films (like ferroelectric films) compared to thermal methods like vacuum evaporation and MBE [11,76,77]. It is worthy to mention that substrate re-sputtering cleaning techniques can also owe their success to the basic mechanism of sputtering process.

For complex ferroelectric film formation, different elements are sputtered at different rates from targets, thus the deposition rate is the key for stoichiometric component of the film, and to a significant extent, kinetic energy of incident ions is vital for the target atoms being dislodged out to form plasma, especially for the compounds containing volatile heavy elements [78] like PZT. Some other factors should also be taken into consideration in sputtering process, including sputtering pressure for the control of surface structure, substrate temperature that determines film growth mode, and other factors like voltage bias and substrate position which are significant for the decrease of defects.

There are several types of sputtering techniques, depending on how the flux of energetic particles that bombards the target surface is created, the kinetic-energy range of the bombarding particles, and the geometry of the system [11], such as on-axis dc magnetron sputtering [79], cylindrical magnetron sputtering [80], off-axis sputtering [81], and ion-beam sputtering [82]. Among them, magnetron sputtering is most widely used. It introduces a magnetic field to bound surrounded charged-particles near the target surface, which successfully overcomes the limitation of low ionization efficiencies in the plasma under high vacuum. With the advent of multi-source deposition, significant advances in sputtering of complex chemical composition materials have been obtained [63,83], such as ferroelectric thin films of PZT [84,85], (Pb,La)(Zr,Ti)O_3_ (PLZT) [86], BFO [87], BST [88], SBT [89], and some other oxides like ZnO [90]. In comparison with PLD, magnetron sputtering requires a higher gas pressure (for complex oxides, pure O_2_ or Ar-O_2_ mixtures are necessary to assure oxygen stoichiometry closing to the desired level) before a plasma is generated. It has noticeable advantages in large-scale deposition in a high speed owing to the bounding and orientating effects of magnetron field, although stoichiometry variation between grown film and target commonly happens due to the different volatilities in the target elements during sputtering. So far, a move towards combining magnetron sputtering with other deposition, or surface modification techniques, in so-called duplex surface engineering processes is rising up, aiming at extending the performance of the component beyond that either process can achieve on its own, and to allow the use of cheaper base materials in high-performance applications [91,92,93].

Alternatively, rather than confine the sputtering source of ions near the target, ion-beam sputtering utilizes a relatively focused “collimated” ion beam to sputter the target. Ion-beam from a separate ion source (e.g., Ar ions) bombards onto the target surface in a certain angle to cause sputtering of the target atoms. In comparison with other sputtering methods, ion beam sputtering have many advantages. On one hand, there is little scattering happens in the sputtering ions due to the very low working deposition pressures with film depositing substrate far away from the ion beam source, resulting in scarcely extra loss. On the other hand, the diffusion process of target [94] can be successfully guaranteed by the focused “collimated” ion beam which possesses high energy. Besides, film deposition can be flexibly controlled as need because of the altered direction of incident ion beam and substrate, as well as the energy and current of the ion beam source. Ion beam sputtering and reactive ion beam sputtering are often engaged in modern researches not only in a variety of thin film deposition including ultrathin ferroelectric films, metal/semiconductor films, special compounds films, *etc.* [95,96,97,98], but also in formation of some complex microstructures like nanodots, thin film ripples, nano-sculpting, *etc.* [99,100,101,102]. Moreover, its application in electron microscopy techniques and re-sputtering cleaning processing is expanding increasingly.

### 2.5. Chemical Deposition Methods

Since materials tailoring becoming the objective of modern materials science, chemical methods for film deposition have edged into commonly used economical methods to obtain thin film with a desired set of properties. In field of growing complex oxides films, such as ferroelectric thin films, chemical deposition methods have set their foot for dozens of years and own an irreplaceable possession on account of their flexible control in chemical components. A variety of approaches including sol-gel, chelate, and metalloorganic decomposition have been employed with success in the fabrication of perovskite materials [103,104,105,106]. Nowadays, some methods have been engaged in ferroelectric thin film synthesis, generally including the widely used chemical solution deposition (CSD) [107,108], chemical vapor deposition (CVD) [109], hydrothermal-electrochemical method [110] and advanced atomic layer deposition [111].

#### 2.5.1. Chemical Solution Deposition

Chemical solution deposition (CSD), also called sol-gel method, is involved in the formation of perovskite thin films in the mid-1980s, begin with the successfully deposition of the desired PZT thin films and engaged in a rapid expansion of research in film–fabrication field [106]. CSD process generally consists of (1) precursor preparation with hydrolysis and refluxing in raw materials; (2) gel dip-coating on substrate; (3) thermal pre-treatment for solvents volatilizing and amorphous films formation; and (4) final thermal treatment for films densification and crystallization. Despite the problems in film qualities such as poor film-compactness, leakage current derived from defects and dislocations introduced during thermal treatment, and unsatisfactory morphology at film surface, absolute predominance can be found in CSD technique. Compared to other film-formation methods, facilities for CSD are simple and the experimental process is easy to control. Uniform films at molecular level can be obtained, and large-scale films can be deposited. Success is obvious in ferroelectric formation through CSD method [112,113], such as SBT [114], Pb-based compounds [115,116], and BFO [117], *etc*. By the way, metalloorganic decomposition (MOD) routes have also been engaged in ferroelectric film fabrication, which is the same as CSD method except for the use of metalloorganic precursors instead of sol.

#### 2.5.2. Chemical Vapor Deposition

Another well-known chemical deposition method is chemical vapor deposition (CVD), whose typical process is that volatile molecular precursors are transported to the substrate and react (maybe decompose) on the substrate surface to produce the desired deposit. Through CVD methods, low temperature deposition on a selected area is achieved and the deposited films are always dense with good step coverage. Many kinds of CVD techniques have been developed including metal-organic chemical vapor deposition (MOCVD), metal-organic vapor phase epitaxy (MOVPE), metal-organic MBE (MOMBE), laser-assisted CVD (LCVD), liquid source CVD (LSCVD), and plasma-enhanced CVD (PECVD), among which MOCVD technique presents the greatest potential for synthesis of complex ferroelectric thin films [118].

In a MOCVD process, the precursors are metal organic materials, which are transferred to a reaction chamber and sequentially experience physicsorb, chemisorb and nucleation with the following film growth process on the surface. The process of MOCVD is sophisticated on account of many chemical reactions. The technique has superiority to other deposition methods with regard to easy and reproducible control of film stoichiometry, conformal coating of arbitrary geometries, excellent film uniformity, high deposition rates, possible formation of multilayers and graded composition layers, high flexibility in doping and substitution of components, and direct growth of epitaxial or polycrystalline textured films without any subsequent annealing [119,120,121,122,123,124,125]. For the ferroelectrics containing volatile constituents (for instance Pb and Bi), the re-evaporation can be well prevented by the perseverance of relative high gas pressure in the MOCVD chamber. Nowadays, despite routine applications in the electronic industry, the MOCVD technique has advanced into an attractive method for ultrathin ferroelectric deposition, which shows great potential in ferroelectric thin-film fabrication in industrial manufacture. Many high quality thin films of oxide materials have been created using the MOCVD technique like PbTiO_3_ (PTO), PZT, STO, SBT, Bi_(4−x)_La_x_Ti_3_O_12_ (BLT) *etc.* [109,126,127,128,129,130].

The availability of appropriate metal-organic precursors has been a challenge for MOCVD in oxide thin-film formation. For many elements with high atomic number like Ba, Sr, Bi, Pb, the precursors typically have limited vapor pressure at room temperature and thus it is essential to heat the bubblers and all the lines in the system at elevated temperatures to avoid clogging. What’s worse, bubbler may undergo “ageing” effects at the elevated temperature, causing poor reproducibility in the long term [131]. Besides, uniform heating is necessary in order to exclude the precursors premature at hot spots or condense at cold sites. The limitation imposed by the necessary high evaporation temperature, has triggered the development of alternative methods of precursor delivery and of new chemical approaches to precursor design [121].

#### 2.5.3. Other Chemical Methods

Apart from the techniques mentioned above, there are other chemical methods engaged in growing ultrathin ferroelectric films. The commonly used ones are atomic layer deposition/epitaxy (ALD/ALE), hydrothermal-electrochemical method, and liquid phase epitaxy (LPE). ALD technique, sometimes called atomic layer MOCVD, deposits alternating monolayers of different elements onto a substrate, combining the feature of MBE and CVD methods. The film achieved is thin, uniform, and aligned with the structure of the substrate. In comparison with MBE, ALD requires lower film growth temperature, which is in favor of preparing not only epitaxial or polycrystalline textured films with abrupt interfaces, but also complex nanostructures. However, the expensive and complex experiment condition and the low growth rate impose restrictions on its application. Recently, ALD has been applied for the growing ultrathin ferroelectric films, e.g., barium and strontium titanates crystalline films [132,133] and BST thin films [134] *etc*. Hydrothermal-electrochemical method deposits desired films in a hydrothermal environment according to the principle of electrochemistry. Growth of ultrathin ferroelectric films utilizing hydrothermal-electrochemical method has been achieved, including film-deposition of STO [135], BTO [136], and BST [137], *etc*. Liquid phase epitaxy (LPE) is another method to grow desired monocrystal thin films from a certain solution onto solid substrates. At conditions that are close to the equilibrium between dissolution and deposition, the film deposition on the substrate is relatively fast and uniform. To facilitate nucleation, and to avoid tension in the grown layer, the thermal expansion coefficient of substrate and grown layer should be similar. With the advantages of simple facility, high depositing rate, good integrality and purity of deposited crystals, some ferroelectric films have been successfully fabricated, among which the most prominent one is LiNdO_3_ and doped LiNdO_3_ [138,139,140,141].

In summary, nowadays the most widely used methods for growth of ultrathin ferroelectric films are mainly five types. They are CSD (sol-gel), MOCVD, sputtering, PLD, and ALD. To be more intuitive, the strength and weakness as well as some common applications of these methods have been compared and listed in Table 2. Readers should get detailed information on film deposition and properties of the deposited films according to their needs.

**Table 2:** Comparison of five mainly synthesis methods for ultrathin ferroelectric films.

Characterization	Synthesis methods				
	CSD (sol-gel)	MOCVD	Sputtering	PLD	ALD
**Stoichiometric ratio**	better	better	ordinary	good	better
**Doping difficulties**	easy	easy	hard	hard	easy
**Precursor obtained**	easy	easy	easier	easier	ordinary
**Adhesion to substrates**	good	good	better	good	better
**Growth rate**	low	high	high	high	lower
**Epitaxial ability**	strong	weak	strong	stronger	strongest
**Uniformity**	better	better	good	good	best
**Thickness control**	hard	easy	easy	easy	best
**Surface morphology**	ordinary	better	good	good	best
**Repeatability**	better	better	ordinary	good	better
**Suitable for large-scare preparation**	ordinarily	better	good	no	better
**Compatibility for heterostructures/superlattices formation**	ordinary	good	good	better	best
**Applicability in ferroelectric film preparation**	Almost suitable for all of the perovskite compounds	Not suitable for films containing elements with high atomic number such as Ba,Sr, Bi, Pb, *etc*.	Restrict to compounds containing volatile heavy elements like PZT	Almost suitable for all of the perovskite compounds besides those cannot be fabricated into PLD targets	Suitable precursors and substrates with appropriate chemical properties are needed for surface chemisorptions

### 2.6. Ferroelectric Thin Film Heterostructures

Ferroelectric thin-film devices are generally thin film heterostructures, which are obtained by depositing ferroelectric thin films onto other film structures or reversely, e.g., substrates, substrate/electrode structures, and semiconductor films, *etc*. From mid-1980s to the early 1990s, ferroelectric thin film heterostructures were usually semiconductor/ferroelectrics/metal structures, which suffered serious devices fatigue resulting from interface defects and mismatch. Therefore, materials which are perovskites and closely lattice-matched to the common perovskite ferroelectrics are demanded. With the rapid advances in integrated microelectronics and optoelectronics, devices with tailored functionality are desperately needed, which pushes fabrication of ferroelectric heterostructures to a vital point for modern integrated ferroelectrics. 

Highly oriented Pt/(La,Sr)CoO_3_(LSCO)/PLZT/LSCO ferroelectric capacitor heterostructures have been successfully fabricated through ion beam sputtering (for Pt) and pulsed laser deposition by Ramesh *et al.* [142]. The introduction of perovskite-structured electrodes of SrRuO_3_ and LSCO commendably solves the mismatch problem of ferroelectric/electrode interface and gives rise to anti-fatigue heterostructures. Then heterostructures which possess fully perovskite structure of (PZT/(La,Ca)MnO_3_) have been reported with new effects [143]. Today, a variety of deposition methods have been exploited for high-quality heterostructures growth including low-temperature sputtering, multi-targets sputtering/PLD, and laser MBE, *etc.*, by which heterostructures with distinct interfaces can be fabricated. Depending on film epitaxial methods and combining various methods together, new efficient techniques are continuously emerging and researches are done for heterostructures formation [123,144,145]. Furthermore, complex heterostructures of ferroelectric/superconductor or ferroelectric/conducting oxide have drawn increasingly attention for their mutual promotion effect of each other and the great prospect in newly integrated ferroelectrics. Ferroelectric heterostructures with high performance could be achieved by “interface engineering”, e.g., by introducing buffer layers between ferroelectric and substrate, or by replacing traditional electrodes like Pt with the conducting oxide (commonly use Y-Ba-Cu-O, LSCO and SrRuO_3_). 

**Figure 5:**
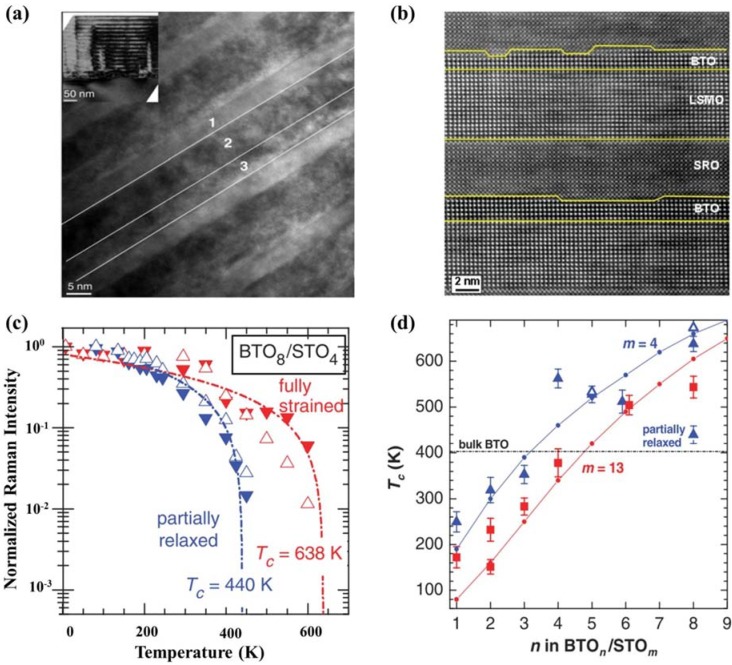
(**a**) Cross section high resolusion transmission electron microscopy (HRTEM) micrographs of BTO, LSMO and SRO layers (marked by “1”, “2” and “3”) [146]; (**b**) cross section high angle annular dark field scanning transmission electron microscopy (HAADF-STEM) micrographs of LSMO–SRO superlattices with BTO interlayers (the lines indicate the interfaces) [146]; (**c**) temperature dependencies of normalized Raman intensities of TO_2_ (solid triangles) and TO_4_ (open triangles) phonons for (BTO_8_/STO_4_) × 10 and (BTO_8_/STO_4_) × 40 (partially relaxed), with the dash-dotted lines being fit to a linear temperature dependence [147]; (**d**) dependence of *T_c_* on n and m in superlattices BTO_n_/STO_m_ (n and m refer to the thickness in unit cells). Blue symbols are for *m* = 4 and red symbols are for *m* = 13. Open triangles are from temperature-dependent XRD measurements. Circles with lines are from the 3D phase-field model calculations. The black horizontal dash-dotted line shows the *T_c_* in bulk BaTiO_3_ [147].

Synthesis techniques with atomic-level control also have shed light on artificially designed ferroelectric superlattices, with their physical properties being explored. Direct observation of superlattice structures have been achieved through microscopic techniques (see Figure 5a,b [146]). Enhanced ferroelectric and dielectric properties have been reported in superlattices composed of BTO (ferroelectric: FE) and STO (paraelectric: PE) layers, which is known to originate from the strong strain coupling of ferroelectric polarization in BTO-based ferroelectrics [147]. Dependence of polarization and *T_c_* on layer thickness and superlattice period has been demonstrated as well (see Figure 5c,d) [147]. CaTiO_3_/BaTiO_3_/SrTiO_3_ ((CTO)*n*/(BTO)*n*/(STO)*n*) superlattices have also been designed and raised an intriguing issue of artificially broken inversion symmetry, resulting in enhanced ferroelectric properties [148,149]. Further studies aiming at optimizing properties of superlattices have been carried out. For example, Seo *et al.* [150] have demonstrated the importance effect of superlattice period on FE polarization, and revealed that both well-strained lattice and proper choice of sub-layer thickness in FE/PE heterostructures are essential for enhancing FE properties in superlattices. Additionally, exchange coupling and exchange bias in (La,Sr)MnO_3_-SrRuO_3_ (LSMO-SRO) superlattices with different nanometrically thin interlayers also have been investigated, attracting many interests [146]. To further reveal the excellent properties of ferroelectric superlattices and to optimize superlattice structure designing, systematically experimental investigations are still underway.

## 3. Characterization of Ferroelectric Thin Films

In addition to the successful deposition of ultrathin ferroelectric films, their properties have drawn enormous attention and stimulate numerical characterization techniques being employed for exploring the properties. The special features of ultrathin ferroelectric films have to be taken into account when borrowing and adapting existing techniques employed in the study of bulk materials to meet the challenges posed by thin-film applications [5]. In general, the characterization techniques can be divided into two kinds, *i.e.*, the ones for material characterization including film composition, structure, and surface, and the ones for property characterization such as the electrical, mechanical and thermal properties of the film. In this section, an overview of characterization techniques for ultrathin ferroelectric films will be presented, aiming at providing a relative comprehensive insight on this aspect.

### 3.1. Material Characterizations

#### 3.1.1. Composition and Structure

Modern techniques have provided access for high-quality ultrathin ferroelectric films which are epitaxial and deposited along a preferred orientation on substrates. As film quality is prerequisite to film properties and is largely influenced by many factors such as deposition and annealing behavior during growth, composition and structure of the obtained films must be firstly characterized. This is especially important for the films deposited through some techniques (for example, sputtering method) which may cause nonstoichiometry. Many techniques have been involved in composition and structure characterizing, mainly including X-ray diffraction (XRD), transmission electron microscopy (TEM), and optical methods like Raman spectrometer, infrared (IR) spectrometer, photoluminescence (PL) spectra, cathode-ray luminescence (CL) spectra, and Spec Ellipsometer.

XRD is an essential tool used for determining the atomic and molecular structure of a crystal, where the crystalline atoms cause the incident X-rays beam to diffract in specific directions. Based on the measured angles and intensities of the diffracted beams, and compared to the standard PDF card, composition and structure information of the film can be determined, including the mean positions, chemical bonds as well as disorder of the atoms. Different from the tests for powders, some of the XRD diffraction peaks cannot be detected for thin films due to the oriented growth on substrates [151]. For polycrystalline ferroelectric films, composition and structure information can be obtained through conventional XRD techniques. For some special films like epitaxial monocrystal thin films, a double-crystal X-ray diffractometer (DCXRD) should be employed for higher resolution and more lattice information. Almost in all of the experimental researches on ultrathin ferroelectric films, XRD occupies a fundamental position [152,153,154]. Besides XRD, the structure information of thin films can be inferred from high resolution electron images and electron diffraction images through TEM tests. On account of the limited testing range of less than 100 nm, TEM is well suited for the study of ultrathin films. From the contrast of a TEM electron diffraction image, either a bright field image or a dark filed image, fine structure with a resolution of about 1–2 Å of the samples can be acquired [155]. Through plan view, dislocation distribution and film growth pattern have been clearly observed [153,156]. Through cross-section view, interface structure information of multilayer films can be well viewed [157,158].

Among the optical methods, Raman spectrometer is a common spectroscopic technique used to observe vibration and rotation modes in a molecular system, relying on Raman scattering of monochromatic light [159]. In the field of ferroelectric thin films, it is utilized for identifications of molecular structure, qualitative and quantitative analysis of film composition and film epitaxial process [146,160,161]. Spontaneous Raman spectroscopy is also used to characterize the crystallographic orientation of a given film as it has characteristic phonon modes. IR spectrometer is another spectroscopic technique similar to Raman spectrometer to analyze molecule vibration modes in samples but according to the infrared absorption spectrum, which can be used to characterize molecular structure and chemical constituents of thin films [162]. On account of the weak light source of conventional IR spectrometer and to increase detecting efficiency, a Fourier transform infrared spectroscopy (FTIR) is commonly engaged in characterizing of ultrathin ferroelectric films [163]. Recently, corporate with microscopy, IR microscopy techniques are exploited not only for timely and precisely study of film composition, structure and morphology, but also for detection of structural phase transition, which has absolute superiority to other techniques of this kind.

Moreover, the electron structure of ferroelectric thin film can be characterized by PL spectra method. Currently, a number of diverse structurally disordered thin films (mostly including ABO_3_ perovskite titanate) have attracted much attention due to their optical properties, which have been intensively studied and characterized through PL spectra [164,165,166]. When luminescence is excited by high-power electron beams (in order of 10 KeV), the acquired PL spectrogram is CL spectrogram. CL, which is commonly an extra part of scanning electron microscopy, has the significant advantage of micro-zone analysis at the range of less than one micrometer, which is superior to other spectral analysis especially for ultrathin films. When it comes to film thickness, Spec Ellipsometer has been popularly exploited to detect film thickness, optical properties, as well as sample roughness on surface and interfaces, giving rise to a new characterizing method for ultrathin films and superlattices [167,168,169].

#### 3.1.2. Surface Information

During the past decades, development of thin film and surface science has culminated an impressive array of surface analytical instruments both experimental and commercial. The current commonly used surface characterizing techniques are summarized as those for surface morphology study including scanning electron microscopy (SEM), scanning probe microscopy (SPM), those for surface composition study including energy dispersive X-ray spectroscopy (EDS/EDX), X-ray photoelectron spectroscopy (XPS), auger electron spectroscopy (AES), secondary ion mass spectroscopy (SIMS), as well as those for surface structure study including reflection high-energy electron diffraction (RHEED), low energy electron diffraction (LEED), and Helium atom scattering (HAS). The following general introduction of these techniques provides readers an overview of these techniques and reference for technique chosen when necessary.

SEM is one of the most important techniques for morphology study of film surface. By detecting signals generated by scanning electron beams, high resolution images better than one nanometer can be achieved. Commonly, the detected signals are the secondary electrons excited by the incident electron beams. The plume of secondary electrons will be mostly contained by the sample with a flat surface; on the contrary, for the sample with a tilted surface, the plume will be partially exposed with more electrons being emitted, giving rise to more strength signal and clearer surface images. Therefore, SEM is conveniently appropriate for the observation of rough surface like nanodots, nanoislands, and nanowires on thin films [170]. For films with smooth surface, SEM may not be an advisable choice for surface morphology test due to the weak recognizability of crystal boundaries. In addition, film cross-section images can be acquired through scanning on transversal surface, from which section information including film thickness can be obtained [116,171]. Moreover, with the development of an environmental SEM, samples can be studied in either high or low vacuum, and even in wet conditions. EDS is often used cooperatively with SEM or TEM, which is a technique used for elements and composition analysis relying on the fundamental principle that each element has a characteristic X-rays spectrum [172]. Rapid analysis can be completed within only several minutes and damage-free testing can be achieved through low energy scanning, but precise quantitative information of elements cannot be acquired [173].

Instead of electron beams, a physical probe is used in SPM to obtain surface morphology images. Interactions between the scanning probe and the testing samples are recorded into two-dimensional or three-dimensional surface images. Currently, the most widely used SPM are STM and AFM. Based on the concept of quantum tunneling and the testing of tunneling current, STM can be used for surfaces imaging and manipulating at the atomic level. It can be used for observing surface structure (including surface defects, absorption, *etc.*) of a certain atom and surface diffusion process at different circumstances such as in ultra-high vacuum, air, liquid or gas ambient, and in a large range of temperatures. Nevertheless, STM generally require conductive samples, making it defective for ferroelectric thin film analysis. This problem is well-settled by AFM. Being a microscopy detecting atom forces, AFM has emerged as the most important technique for surface imaging and measuring on the nanometer scale, especially for the non-conducting and flat ultrathin ferroelectric films in comparison with SEM and STM. Thus AFM has been widely engaged in ferroelectric thin film studies [174,175]. Finally, it is noteworthy that SPM techniques have common drawbacks in limited scanning area, slow scanning speed and restriction of scanning probe. 

For more accurate analysis on film elements and chemical states, XPS technique can be adopted. XPS is a surface-sensitive spectroscopic technique under high vacuum with the detecting depth of 1–10 nm, whose spectra are obtained by measuring the kinetic energyand number of escaping electrons caused by the incident X-ray beams. When ions are employed as stripping tools for samples, intensive analysis can be established on the newly exposed surfaces. Despite the advantages in high sensitivity and damage-free analysis, XPS meets challenges in its detecting limit that only those element contents higher than 0.1% can be detected. Furthermore, the irradiated area of X-rays is relatively large, resulting in the weak focus range of millimeter size. Compared to XPS, AES technique, a spectroscopic technique based on the analysis of energetic electrons emitted from an excited atom after a series of internal relaxation events underlying the Auger effect, can be employed for surface elements identification in a smaller area (even less than 30 nm) and higher focusing depth. However, chemical states of film surface cannot be absolutely sure due to the absence of standard spectrum data. Moreover, elements of Hydrogen and Helium cannot be detected by XPS or AES because of the absence of inner electrons, which is the curtail members being actuated and emitted in both of the techniques [176,177,178]. 

In addition, ion beam analysis technique like SIMS and Time of Flight SIMS (TOF-SIMS) also can be used for composition analysis of ferroelectric film surface in a depth of 1–2 nm [179,180]. RHEED and LEED are alternative sensitive surface characterizing techniques for atomic surface structure and morphology information relying on electron diffraction, which can be simultaneously applied in one multifunctional surface analyzer, promoting researches on film surfaces to a great extent [181,182]. HAS technique, which provides information about the surface structure and lattice dynamics, should be useful to establish surface researches on sputtering, annealing, and adsorbed-layer depositing of ferroelectric thin films [183,184,185].

To be more forthright, the main surface characterizing techniques have been summed up and enumerated in Table 3 [5], providing a quick overview.

**Table 3:** Commonly used material characterizing techniques for ultrathin ferroelectric films [5].

Techniques		Characterizing applications	Primary beam	Secondary signal
**XRD**		Crystal composition and structure	Photon (>1 keV)	X-ray
**TEM**		High-resolution structure	Electron (100–400 keV)	Electron
**SEM**		Surface morphology appropriate for tilted surface	Electron (0.3–30 keV)	Electron
**SPM**	**STM**	Surface morphology and manipulation for conductors or semiconductors at atomic scale	None (based on physical probe)	None (based on physical probe)
	**AFM**	Surface morphology appropriate for flat surface		
**EDX/EDS**		Surface region composition	Electron (1–30 keV)	X-ray
**XPS**		Quantitative surface composition	Photon (>1 keV)	Electron
**AES**		Quantitative surface layer composition in micro-region	Electron (500 eV–10 keV)	Electron
**SIMS/TOF-SIMS**		Trace composition *vs* depth	Ion (1–15 keV)	Ion
**RHEED**		Surface structure	Electron (30–50 keV)	Electron
**LEED**		Surface structure	Electron (20–200 eV)	Electron
**HAS**		Surface structure and lattice dynamics	Monochromatic helium beam	Diffracted atoms
**Raman spectrometer**		Composition and molecular structure within Raman active mode	Optical wave	Optical signals
**IR/FTIR**		Composition and molecular structure within IR active mode		
**PL**		Electron structure, band gap, impurity grade, defects, atomic arrangement		
**CL**		Similar to PL, special at micro-zone analysis (<1 μm range)		
**Spec Ellipsometer**		Film thickness, optical properties, sample roughness on surface/interfaces		

### 3.2. Property Characterizations

Except for essential properties of composition, structure, and surface information, ferroelectric thin films have dominant advantages superior to other materials in their unique and valuable properties of polarization behavior, domain structure, transport properties, thermal properties and mechanical properties, which leads to a strong need for extensive investigation of the nanoscale properties in ferroelectric materials, and thus yields substantial advances in development and optimization of characterizing techniques on these properties.

#### 3.2.1. Polarization Switching and Hysteresis Loop

**Figure 6:**
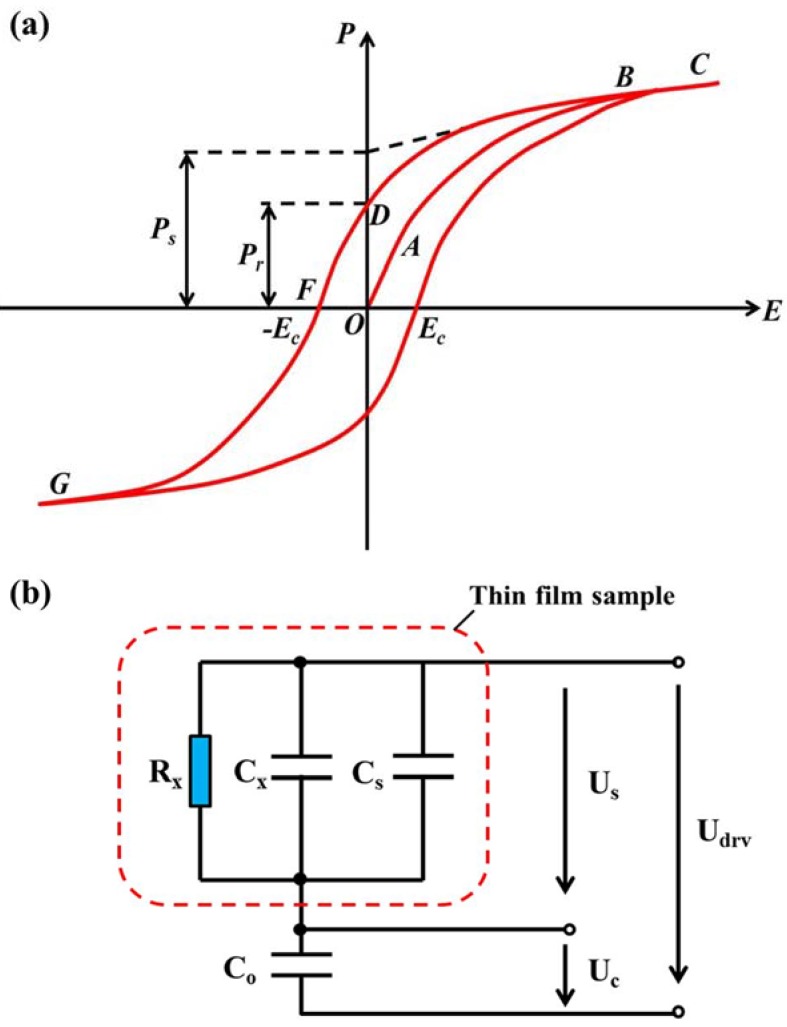
(**a**) Sketch of a typical ferroelectric hysteresis loop, *i.e.*, polarization–electric field (P-E) hysteresis loop; (**b**) typical Sawyer–Tower circuit for thin film test.

Ferroelectrics have a distinguishing property that they possess a spontaneous polarization *P_s_* that can be reversed by an applied electric field, yielding a nonlinear hysteresis loop (see Figure 6a). Materials exhibit ferroelectricity only below a certain phase transition temperature of Curie temperature (*T_c_*), and are paraelectric above this temperature. A typical Sawyer–Tower circuit [186], which is shown in Figure 6b, is essentially used for experimental ferroelectric hysteresis loop measuring below *T_c_*, and attempts have been done to the exploitation of modified Sawyer–Tower circuits for more accurate and strength measurement [187,188]. For the test, a capacitor of electrode/ferroelectric/electrode should be fabricated. Pulsed signals in square-wave or sine-wave form are usually used for the study of polarization switching. Ferroelectric information including the remnant polarization of *P_r_*, the spontaneous polarization of *P_s_*, the coercive field *E_c_*, and even ferroelectric loss can be acquired through a hysteresis loop. In polycrystalline materials, true spontaneous polarization equal to that of a single crystal can never be reached and it is more correct to speak of saturated rather than of spontaneous polarization. Note also that the coercive field *E_c_* that is determined from the intercept of the hysteresis loop with the field axis is not an absolute threshold field. It is possible that the polarization will eventually switch under an applied low-value electric field for a long time [189,190,191].

It has been widely recognized that domain structure has great impact on the polarization switching behavior. The shape of hysteresis loop, *i.e.*, the coercive field, spontaneous and remnant polarization, can be also affected by many factors such as the device factor including thickness, mechanical stresses, film fabrication conditions, the presence of charged defects, and interfaces of thin film and electrode, as well as the testing conditions including temperature, applied electric field, and test frequency, *etc*. Moreover, the polarization behaviors are strikingly different between ultrathin films and bulk materials especially in the magnitude of the coercive field, which is higher in thin films than in bulk materials and shows a decrease with an increase in the film thickness [192,193]. This phenomenon will be discussed in Section 4.

In addition, it is worth noticing that a closed loop in the graph of switched charge Q *versus* applied electric voltage V cannot be the evidence of ferroelectricity. Closed Q-V loops have been reported in non-ferroelectric materials such as some spurious artifacts and even ordinary household objects like bananas [194,195,196,197]. These non-ferroelectric objects can exhibit very misleading “hysteresis” loops which are nearly identical to those misinterpreted as ferroelectric hysteresis loops in crystals, thus providing completely meaningless coercive field and remanent polarization values. ‘Bananas’ (nicknamed by Scott [194]) exhibited by non-ferroelectric objects or cigar-shaped loops which are typical in lossy dielectrics, have very little to do with ferroelectricity. For a normal ferroelectric hysteresis loop, it should be saturated and has a region in Q-V loop that is concave [194]. Electrical hysteresis measurements determine only switched charge Q, which can be influenced by polarization, space charge (e.g., oxygen vacancies) and leakage currents in ferroelectric thin films. Well-designed tests with proper parameters and conditions (e.g., a series of testing frequency and appropriate modes of the applied electric field) may to some extent separate them from each other. Besides, it is possible to measure polarization magnitudes and directions in crystals optically (for instance) via second harmonic generation [198].

Moreover, even though a normal hysteresis loop is obtained, a simple hysteresis loop measured at a single frequency is not always solid evidence of ferroelectricity in systems such as thin films or nanoscale structures. A ferroelectric-like hysteresis loop can be obtained in a non-ferroelectric system consisting of two back-to-back metal-semiconductor Schottky contacts with a large concentration of traps distributed over a finite thickness near the electrodes [196]. In particular, for the capacitors which are composed by ultrathin ferroelectric films, the influence from electrodes and electrode/film interfaces, which probably dominates the electrical response, must be carefully taken into consideration.

#### 3.2.2. Domain Structure

The mechanism of polarization switching has been studied in detail for many ferroelectrics [199,200]. Although complex factors may have influence on the switching behavior, it is widely believed that domain structure in ferroelectrics is the initial factor in polarization switching as the consequence of domain-wall motion, growth of existing antiparallel domains, as well as nucleation and growth of new antiparallel domains [199,201]. Therefore, ferroelectric domain structure, which is derived from the surface effects, nonuniformity and mechanical constraints, is in desperate need to be well characterized. For ferroelectric thin films, the most commonly used characterizing methods are piezoresponse force microscopy (PFM) and electron microscopy (e.g., SEM and TEM), which will be particularly summarized and introduced in this section. Besides them, there are other techniques that contribute to observation of domains, mainly including X-ray scattering [202,203], optical characterization [204,205], powder deposition technique [206], nematic liquid crystal (NLC) method [207], pyroelectric probe technique (PPT) [208], *etc.*, here we will not cover it all, information in detail can be looked up in the papers cited above.

**Figure 7:**
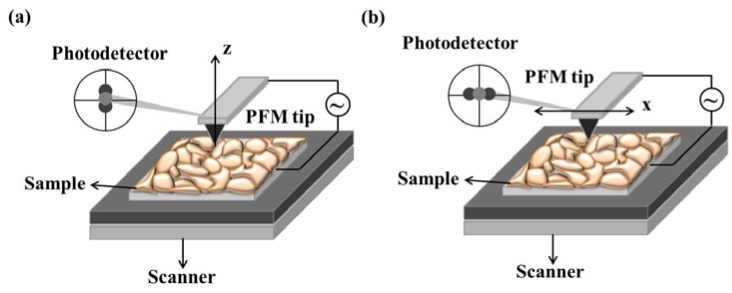
Schematic diagrams of (**a**) vertical piezoresponse (VPFM) and (**b**) lateral PFM (LPFM) [209].

Belonging to SPM technique, piezoresponse force microscopy (PFM) is based on the detection of local piezoelectric deformation of a sample induced by an external electric field [209]. It opens new venues in domain-related characterizing, giving rise to not only nondestructive visualization of nanoscale domain structures in ferroelectric thin films, but also nanoscale domain controlling for such applications as high-density data storage and ferroelectric lithography [210,211]. Usually, PFM based on dynamic piezoresponse is utilized for high-resolution imaging of domain structures, for the reason that the domain imaging based on static piezoelectric deformation is difficult to implement besides some limited cases (e.g., samples with very smooth surface, relatively high values of piezoelectric constants and coercive fields). The dynamic PFM method has the ability in domain delineation with polarization both vertical and parallel to the sample surface, which is the so-called vertical piezoresponse (VPFM) and lateral PFM (LPFM) [212,213]. Schematic diagrams of VPFM and LPFM have been respectively shown in Figure 7a,b [209]. In VPFM mode (see Figure 7a), when an AC modulation voltage (always a sine-wave or cosine-wave signal) is applied on the sample, surface displacement happens on the thin film, which can be measured using a standard lock-in technique by detecting the vertical vibration of the cantilever, which follows sample surface oscillation. By scanning the surface while detecting the first harmonic component of the normal surface vibration, a domain map in out-of-plane directions can be acquired. In LPFM mode (see Figure 7b), depending on the relative orientations of the applied field and the polarization vector, sample deformation can be in the form of elongation, contraction or shear. On account of the piezoelectric shear deformation, vibration in the direction parallel to film surface can be generated by the applied modulation voltage across the sample. This surface vibration, translated via the friction forces to the torsional movement of the cantilever, can be detected in the same way as the normal cantilever oscillation in VPFM [209].

Another significant feature of PFM is the capability in manipulating the domain structure. When a small DC voltage is applied between the conductive probing-tip and bottom electrode, an electric field of several hundred kilovolts per centimeter will be generated, which is higher than the coercive voltage of most ferroelectric thin films, thus inducing local polarization reversal. The technique has for the first time provided an opportunity for controlling ferroelectric properties at nanoscale and direct studies of the domain structure evolution under an external electric field, which has been applied to ultrahigh density data storage and ferroelectric lithography [209,210,211]. Furthermore, PFM should be an excellent instrument in measurement of local hysteresis loops at approximately 10 nm levels through applying stepped or pulsed voltage. Information on local electromechanical activity and coercive voltage variations between dissimilar grains can be acquired from a PFM hysteresis loop. An advanced approach of three-dimensional (3D) PFM, which is based on combination of VPFM and LPFM, has been applied not only to differentiate 90° and 180° domain switching but also to perform complete reconstruction of polarization in ferroelectric thin films such as PTO, BTO, PZT and BFO [214,215,216,217,218], *etc*.

Currently, PFM has emerged as a powerful tool for high-resolution characterization due to its advantages of universality to virtually all types of materials and casual testing conditions without vacuum. For film samples as ultrathin ferroelectric films, a bottom electrode is needed for film testing, which leads films being deposited on thin metal layers like Pt or Au. Despite the excellent features of PFM technique in robust qualitative domain imaging, quantification domain imaging for ferroelectric thin films cannot be perfectly completed so far due to the complexity of the tip-sample interaction which involves electromechanical and electrostatic components. Experimentally, some factors are supposed to have impact on the testing results and should be taken into consideration. For instance, physical properties of the samples such as film thickness, dielectric constants, orientation, defect structure, crystallinity, electrode material, *etc.*, and testing conditions such as applied voltage, frequency, loading force, cantilever force constant, tip apex radius, ambient environment [209,219]. On account of the poor time resolution of PFM, more challenges come in the observation of the polarization switching occurring within a matter of microseconds and faster, which is several magnitude of the time required for image acquisition in PFM (often need several minutes). Therefore, PFM techniques possess unsuitability *in situ* measurements of domain dynamics with fast polarization reverse, while readily possess comparative suitability in investigation of slow polarization relaxation processes with characteristic times of the order of minutes and above. The method of characterizing domain structure dynamics in a quasi-static regime using step-by-step switching has been applied to thin films [210].

Another technique of electron microscopy also possesses a dominant position in domain characterization for their ability in visually high-resolution observation of domain structure and domain evolution under an applied field (commonly an electric field). TEM and SEM are commonly important members of electron microscopy engaged in domain observation. TEM is a widely used unique tool to study domain structures in crystals, which not only dues to its possibility of in-situ and high-resolution study at a small area in direct space as well as in reciprocal space with a known orientation relationship between both, but also because of its ability in observation of defects related to domain behavior in crystals. Different kinds of domain walls can be differentiated from comprehensive analysis of the obtained diffraction contrast images, diffraction patterns, and high resolution images. To carry out domain observation researches through TEM techniques, sophisticated pretreatment for sample should be preceded. Commonly, plan view TEM samples should be thin foils, which can be prepared either by dimpling and grinding or wedge polishing followed by single-sided ion milling either on a liquid nitrogen cooled stage or at ambient temperature [220]. For ultrathin ferroelectric films, the pretreatment procedure should be relatively easier than bulk materials due to the small film-thickness.

Diffraction contrast images from TEM are maps of the intensity distribution in a diffraction spot (dark field image) or in the incident beam (bright field image), which are actually magnified diffraction spots [221]. Considering the intensity of the diffracted as well as transmitted beams is a sensitive function of the deviation in the exact Bragg condition, domains where the lattices differ in orientation can be revealed as regions of different brightness in the bright or dark field image under two beam conditions, giving rise to alternative identification of domain structures. Additionally, from diffraction patterns of TEM, the symmetry elements that connect with domain structures on either side of the interface can be determined by the diffraction pattern taken across an interface, and domains related by rotations can be identified in a straight forward manner from the diffraction pattern along the zone parallel with the rotation axis. Moreover, atom columns parallel with the electron beam can be imaged simultaneously in different domains directly from TEM, which gives an access to direct visualization on the symmetry relation between domains from high resolution images of TEM. Besides, the projected displacement vector can be read-off directly from these images with the access to study the translation interfaces. In a weak-beam TEM, by fitting simulated fringe profiles to experimental ones, a quantitative analysis of the thickness fringes that appear on weak-beam images of inclined domain walls can be established to extract the thickness of the domain walls in a quantitative way [222,223,224]. Furthermore, quantitative measuring of the local polarization and investigating on the domain structures can be realized due to the probability in imaging the local polarization dipoles at atomic resolution [225]. A large number of works have reported on the observation of domain structures, evolution, as well as switching of ferroelectric thin films through TEM [155,220,226,227,228,229,230,231]. For more detailed information, one can refer to other more professional papers and books [221,232].

For film surface research, SEM should be a previous choice for its direct visualization on surface. Usually, samples for testing need pretreatment of etching and even surface coating methods, for the reason that different etching degree will happen on domains with different polarity, resulting in the rugged area on surface which can be easily distinguished by SEM. In a widely used field emission SEM (FESEM), domain observation in SEM should owe its success to voltage contrast by imaging the sample at its upper crossover voltage typically 2–5 kV, which is the accelerating potential of the electron beam where the number of incident electrons from the primary beam equals the total secondary and back-scattered electron yield from the sample. Thus, no surface charge will result from imaging with an electron beam, leading to domains being differentiated based on their sense of polarization. The positively terminating domains on surface will attract escaping secondary electrons back to the surface and appear dark, different from those negatively terminating domains repelling any secondary electrons and appearing relatively bright, resulting in the SEM domain images we observed. Experimentally, the voltage contrast, which can be obtained from an accelerating voltage window in SEM, will weaken with deviation from the true crossover voltage as the surface charge created by the primary beam increasingly masks the surface charge due to polarization [220]. An environmental SEM (SSEM) is sometimes needed especially for insulating ferroelectric materials, for the reason that it can operate at pressures 10,000 times higher than that of a standard SEM, with an opportunity to examine unprepared and uncoated insulating samples [233]. A scanning electron acoustic microscopy (SEAM), which is integrated with electron-optic techniques, electron audio techniques, and piezoelectric sensors, has been employed for damage-free and high-resolution imaging of domains [234]. With the development of SEM techniques, many studies have been successfully established on ferroelectric domain observation through SEM techniques, such as PTO, PZT, BTO, Bi_4_Ti_3_O_12_, LiTaO_3_, KTiOPO_4_ (KTP) [235,236,237], *etc*.

#### 3.2.3. Transport Properties

Transport properties, which are usually characterized in current-voltage (I-V) relationships, are also factors of great significance in ferroelectric thin films. With respect to thin films, I-V test always begins on an electrode/films/electrode capacitor in an automatic way via equipments such as Keithley 4200 semiconductor characterization system (SCS) (Keithley Instruments Inc., Cleveland, OH, USA), RT66 ferroelectrics test system (Radiant Technologies, Albuquerque, NM, USA) and conductive AFM (c-AFM). Since ferroelectric films prepared by normal routes are often complex polycrystalline with a fine-grained microstructure, significant leakage currents may be caused by a host of factors such as grain boundaries, defects, conduction processes such as Schottky injection or Fowler–Nordheim tunneling *etc.* Considering the complicated conditions in ferroelectric thin films, some intensive researches have been carried out on conduction mechanism of ferroelectric thin films and some theories have been put up including Schottky emission mechanism [238], space charge limited conduction (SCLC) theory [239,240], and grain boundary limited conduction (GBLC) [241], *etc*. Generally speaking, leakage current is one of the most concerned issues for device application of ferroelectric films, which is usually carried out at a specified voltage and is desired in many cases as small as possible [242]. Current-time (I-t) measurements with sufficient time should be taken into account to obtain true steady-state leakage current instead of relaxation current [243], from which resistively degradation can also be investigated due to its intrinsically time-dependent relation with DC conduction of the films under constant DC field [244]. In contrast to leakage current I–V curves, the corresponding current–voltage loops can be obtained during voltage sweeps with cycled voltage. The loops coinciding with the polarization–voltage hysteresis loops show clear switching peaks at the coercive voltage of the sample, which can be a criterion to differentiate ferroelectrics with leaky dielectric [11]. Currently, the resistive switching effect, which is supposed to have a relationship with the regular motion of charge carriers (usually oxygen vacancies) in films, has been reported in many ferroelectric thin films [245,246,247], attracting increasingly attention in studies about transport properties of ferroelectric thin films. Giant electroresistance (GER) is another effect related to transport properties special in ultrathin ferroelectric films [248,249,250]. More detailed introduction on transport properties of ultrathin ferroelectric films can be found in Section 4.

#### 3.2.4. Dielectric Permittivity

A capacitor configuration in which a ferroelectric film is sandwiched by two electrodes is needed for the testing of dielectric permittivity. Therefore, incomplete charge compensation, from the finite free charge carriers in the conducting electrodes to the abundant polarization bound charges induced at the surfaces of ferroelectric layer, will happen and induce a depolarization field (*E_d_*), which will have increasingly larger impact on polarization and permittivity of the capacitor with the decrease of film thickness [251,252,253]. In general, an impedance analyzer with an applied small-amplitude ac signal (usually a small ac ripple superimposed on a dc voltage) is needed in experimental study, where the real and imaginary parts of the impedance will be measured and balanced with a set of references inside, giving rise to the capacitance and loss afterwards being calculated automatically by the machine. A characteristic “butterfly loop” of capacitance voltage relationship is usually acquired in ferroelectric materials because their capacitances appear different behavior as increasing and decreasing voltage. Impedance spectroscopy can be obtained when a series of different frequency of the ac signal is applied on the capacitor, from which information on the time scales at which processes operate can be received [195]. In particular, since the permittivity maximum of thin films, which depends much on film thickness, not necessarily occurs at the transition temperature of ferroelectric thin films, the traditional methods that measuring the transition temperature of bulk materials through the measurement of dielectric constant and loss will make no sense [254,255]. For ultrathin ferroelectric film-capacitors, especially those with film-thickness near tens of unit cell length, the permittivity contributed by electrodes (ε_e_) has a striking influence on the whole permittivity and must be taken into account. This can be carried out through optical spectroscopy measurement with a series of subsequent analyses, for instance, ε_e_ of high-quality ultrathin BTO capacitors have been elaborately measured and analyzed [256].

#### 3.2.5. Mechanical Properties

**Figure 8:**
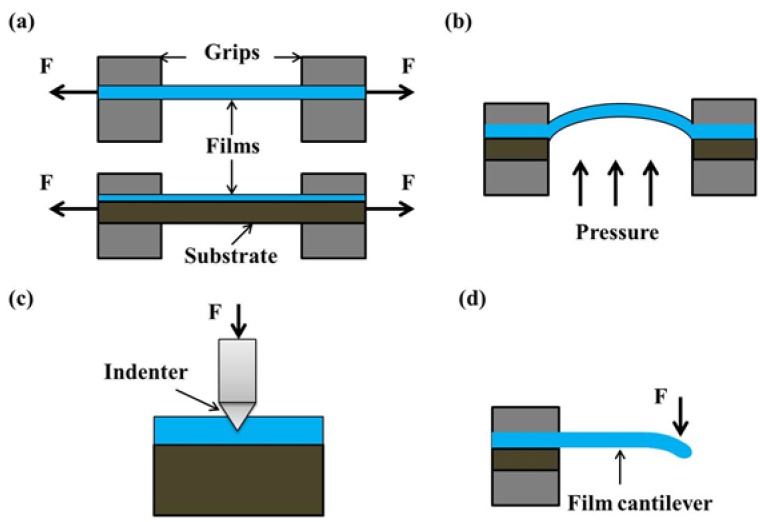
Methods for mechanical characterizing of thin films from the response to the applied stresses [5]: (**a**) tensile testing; (**b**) bulge testing; (**c**) indentation (micro or nano) hardness testing; (**d**) deflection of microbeams.

Mechanical properties of the ferroelectric thin film are important in determining the film structure and behaviors, as the film is often subjected to mechanical loads, e.g., lattice mismatch strain from the substrate, inhomogeneous strains caused by defects, and strain caused by incompatibility of domains. As devices continue to shrink in size, stress effects on film structures and properties must be well understood and measured. The mechanical properties characterization for thin films can be divided into two kinds, one is to characterize the important mechanical properties of films such as Young's modulus *Y*, yield stress σ_0_, ultimate tensile stress σ_UTS_, and film hardness *H* from the response to the applied stresses, the other is to measure internal stress of unloaded films which is largely rooted in concepts of elasticity. For the former one, commonly used methods are tensile testing, Bulge testing and nanoindentation testing (including nanohardness and deflection of microbeams), which have been schematically depicted in Figure 8 [5]. 

Strains are typically measured through optical methods or X-ray based methods. Novel micro-tensile testing devices have been incorporated within electron microscopes, enabling direct observation of defects and recording of diffraction patterns during straining. Tensile testing, which usually employ X-ray and optical methods in measuring process [257], is appropriate for bulk material testing but far from routine for thin films, because of the experimental challenges in detaching thin films from substrates and handling them during the whole measurement. By contrast, Bulge testing is more widely used for film testing, which introduces lithography and etching methods to remove film substrates, depending on measurement on the maximum height of the resulting hemispherical bulge in the film optically with a microscope or interferometer and finally converted to strain [258]. Furthermore, for the films integrated in devices for application which cannot be detached from substrates, nanoindentation testing which can express nanohardness and microbeam deflection (which is indenter induced deflection of cantilever microbeams which obtained through photolithographic and etching processes similar as microelectronics technology) should be useful [259]. A nanoindentor is always employed for film testing on a small volume of 1 μm^3^, and resolutions better than 0.01 μN for load and 0.01 nm for displacement have been claimed in the better instruments [5,259]. Additionally, microbeams can also been micro-machined at different locations of wafers to locally monitor stress variations in subsequently deposited thin films due to their tiny size [260,261]. For the testing of internal stresses in thin films, there are basically two general categories of experimental techniques for measuring, including the one relies on measuring the deflection or curvature of substrates to which the films are attached in real time during film growth through some techniques like laser-scanning methods with the smallest detectable stress of ~0.2 MPa in film thickness of 100 nm [262]; the second involves the direct determination of elastic strains by means of X-ray diffraction methods [263] with the smallest detectable stress ~50 MPa in film thickness of 100 nm [132]. Different testing methods have respective advantages and the sensitively detectable stress can be looked up in other detailed books [5].

#### 3.2.6. Thermal Properties

When mentioning about thermal properties of ferroelectric thin films, pyroelectric effect is the most important one. It refers to the change of the electric polarization *P* induced by a temperature *T* change, which can be quantified by the pyroelectric coefficient *p* (*p* = *dp* / *dT*) [264]. In ferroelectrics, pyroelectric effect usually exists below a certain transition temperature of the Curie point (*T_c_*) [265], changes in pyroelectric current in a heating and cooling process can be evidence for appearance and disappearance of polarization. There is increasing interest in ferroelectric pyroelectric sensors which on the basis of pyroelectric properties with promising potential in device application, thus urges more reliable characterizing of thermal properties. Although a variety of methods have been exploited for measurements of pyroelectric coefficients, most of which can be generally divided into two kinds [199]. One is to directly measure the pyroelectric current and at the same time vary the temperature. The other is to measure the polarization or charge which can be estimated either by the integration of pyroelectric current during the continuous heating of the sample or recording the hysteresis loop at various temperatures by the Sawyer-Tower technique [265]. The related induced charge can be measured either through the induced voltage or the induced current, carried out respectively through an ‘open circuit’ method (measured with a high impedance voltmeter) and a ‘short circuit’ method [266]. The temperature changes *dT* can be achieved through many methods generally including the use of a chopping incoming radiation source or AC heating (known as Chynoweth technique [267]), Byer-Roundy methods [268], and heating systems which always based on a heating plate, *etc*. Based on the methods mentioned above, some attempts have been done to develop new testing methods for pyroelectric coefficient measurement, among which more straightforward and accurate ones depending on newly designed circuits are preferred [269,270,271]. Moreover, for pyroelectric materials, a change of polarization can also lead to temperature change, namely an electrothermal effect, which is a reverse effect of pyroelectricity [272]. The electrothermal conversion of this kind of materials also have been experimentally measured using a differential scanning calorimetry upon the application of an electric field, for instance, 0.75(PbMg_1/3_Nb_2/3_)-0.25(PbTiO_3_) (PMN-PTO) [273]. 

## 4. Important Phenomena in Ferroelectric Thin Films

With the trend of device miniaturization, progresses achieved in fabrication and characterization methods of ferroelectric thin films have pushed the investigation on properties to nanoscale level, giving rise to the development of ultrathin ferroelectric films with a variety of intriguing phenomena that are significantly distinguished with those of larger size ferroelectric systems. These include ferroelectric stability, electromechanical behaviors, domain structure and related properties, electronic transport behaviors, as well as ferroelectric fatigue, *etc*. In this section, we will review the developments of researches, mainly pay close attention to experimental researches, on important phenomena in ultrathin ferroelectric films and discuss the challenges and prospects. The physics behind these phenomena would be mentioned briefly (readers interested in the theory of ferroelectricity thin film properties can refer to [11,195,274]).

### 4.1. Ferroelectricity and Critical Thickness

Due to the collective nature of ferroelectricity and its interplay with surface/interface, ferroelectric thin films generally show size dependence of ferroelectric behaviors, namely ferroelectric size effect. An important concept related to ferroelectric size effect in ferroelectric thin films is critical thickness, which is defined as the minimum film thickness that can maintain ferroelectricity. Since the concept of critical thickness in ferroelectric thin films is put up, a large amount of researches both theoretical and experimental have been carried out on it. According to the phenomenological theory, the critical thickness of a single-domain ferroelectric thin film is a result from the competition between bulk free energy, surface energy and depolarizing energy [275,276,277]. The bulk free energy and surface energy can be strongly modified by mechanical strain (e.g., misfit strain, strain of defects and surface tension) due to coupling between mechanical strain and polarization. The depolarizing energy, manifested with a depolarization field, is caused by incomplete compensation of bounded charges, which tends to suppress ferroelectricity/phase transition and thus increase the critical thickness. For ultrathin films, as film thickness is reduced, finite screening occurring at ferroelectric/electrode interface begins more significant on affecting the ferroelectricity stability. Based on the former discussions, we would like to discuss the ferroelectricity and critical thickness of ultrathin ferroelectric films from two aspects: strain effects stemming from substrate misfit strain and structural defects, and effects of electrodes, mainly focusing on experimental studies. 

#### 4.1.1. Effect of Substrate Misfit Strain and Structural Defects

Both homogeneous and inhomogeneous strains in ultrathin films have substantial impact on ferroelectricity stability of the film. Among various strain sources, lattice mismatch between the film and the substrate is an important origin of film strain, which has decisive effects on the structure and properties of ultrathin ferroelectric films [278,279]. Particularly, growth of epitaxial films on appropriate substrates provides the opportunity to control film strain state and to tailor the ferroelectric behaviors. Strains can also arise from structural defects such as dislocations, cracks and voids formed during film deposition or loading processes. The ferroelectric stability and related properties of the thin films not only vary strongly near the “defective” regions due to the highly inhomogeneous strain field, but also can be overall affected in a similar way as substrate misfit strain as defects may also contribute to a homogenous strain. In general, homogeneous and inhomogeneous strains always coexist in ultrathin ferroelectric films and have substantial impact on film properties including static ones, e.g., transition temperature, critical thickness, dielectric permittivity, coercive field, domain structure, and dynamic ones, e.g., domain switching, and piezoelectric responses, *etc.*

In regard to film critical thickness, the first important experiment value of critical thickness has been obtained in 1998 by Bune *et al.* [280], who reported measurements of ferroelectric transition in Langmuir–Blodgett (LB) crystalline P(VDF-TrFE) films of just 10 Å (two monolayers) thick. These films are considered as two-dimensional ferroelectrics due to the near-absence of finite-size effects on bulk transition. Since then, with the help of *ab initio* calculations [252], experimental data on critical thickness of ferroelectric films, especially those with perovskite structures blossoms. A significant experimental progress was achieved in the year of 1999, when Tybell *et al.* [281] demonstrated a stable ferroelectric polarization in atomically smooth Pb(Zr_0.2_Ti_0.8_)O_3_ films down to thicknesses of 40 Å (ten unit cells) using off-axis RF magnetron sputtering. The striking result is well in accordance with former calculations that predict ferroelectricity down to monolayer thicknesses. Later theoretical researches were devoted to verify whether the limit in film thickness could be pushed further, which significantly simulated and guided subsequent experimental investigations. For ultrathin films, direct experimental quantification of ferroelectricity are complicated by extrinsic effects such as leakage, therefore methods other than the traditional ferroelectric hysteresis loop have been reported to characterize the stability of the polar state, (see, e.g., the typical one by Streiffer *et al.* [282]). In 2004, Nagarajan *et al.* [283] conducted a systematic experimental study of the switched polarization in (001) epitaxial Pb(Zr_0.2_Ti_0.8_)O_3_ films of thickness down to 4 nm in the presence of SrRuO_3_ electrodes based on a Radiant resting system in combine with an AFM based probing technique. Later in the same year, meritorious advance was made by Fong *et al.* [253], who reported a stable ferroelectric phase in epitaxial PbTiO_3_ films grown on (001) SrTiO_3_ substrates with film thicknesses down to 1.2 nm (3 unit cells) at room temperature, which implied that no thickness limit is imposed on practical devices by an intrinsic ferroelectric size effect, *i.e.*, an absence of critical size. Their intensive synchrotron X-ray study also indicated that 180° stripe domains are very effective at neutralizing the depolarizing field and stabilizing the ferroelectric phase in ultrathin films. Meanwhile, the suppression of ferroelectricity in ultrathin films with substrate misfit strain has been observed. For instance, Sun *et al.* [284] reported that the critical thickness for misfit dislocations to occur in PbTiO_3_ films which were grown on (100) SrTiO_3_ substrates was found to lie between 2 and 4 nm. They studied the formation of the dislocation array and found that the percentage of misfit strain relaxation through the formation of a dislocation array is a function of film thickness, indicating gradual relaxation of mismatch strain with increasing film thickness.

These researches have intensified debates about whether critical size exists in ferroelectric materials. Intuitive experimental evidence was put forward in the year of 2007. Jia *et al.* [285] carried out researches on ultrathin PTO films on SrRuO_3_-buffered STO substrates by means of TEM. They observed a systematic reduction of atomic rumplings at the substrate/film interfaces, suggesting that interface suppression of the ferroelectricity plays a critical role in the ferroelectric size effect. Afterwards, there have been intensive works on broader range of materials. For example, Bastani *et al.* [286] reported a critical thickness of 50 nm in highly (100)-textured Pb(Zr_0.53_Ti_0.47_)O_3_ films in 2011. Strong polarization decays in ultrathin strained (001) BFO films below a critical thickness of 5–7 nm were reported by Rault *et al.* [287] in 2012. Recently, many experiments exploited new ways to expand the critical thickness to a smaller range. In 2009, Garcia *et al.* [250] have demonstrated that highly strained BTO films retain robust room-temperature ferroelectricity down to 1 nm. In 2013, highly ordered two-dimensional arrays of PZT nanorods patterns with a notable thickness of approximately 10 nm have been successfully fabricated on n-doped Si substrates by Varghese *et al.* [288], which showed obvious ferroelectric behavior with the thickness close to the critical size limit of PZT. The excellent experiment results have to some extent provided a new way for decrease critical thickness of materials through fabrication of other nanostructures. 

In addition, the strong coupling between strains and ferroelectricity leads to shift of phase transition temperature *T_c_* and related properties*.* Early in 1998, Specht *et al.* [289] had demonstrated variation of *T_c_* with layer thickness in KTaO_3_/KNbO_3_ strained-layer superlattices. *T_c_* shift of more than 100K was observed due to the interlayer coupling of strain in superlattice. Subsequently, Streiffer *et al.* [282] and Fong *et al.* [283] carried out a series of experiments on the influence of epitaxial strain on phase transition temperature, with significant impact being verified. Later in 2003, novel dielectric property different from that of independent SrTiO_3_ and BaZrO_3_ thin films had been reported in SrTiO_3_/BaZrO_3_ superlattices by Christen *et al.* [290], which was most likely because of strain-induced room-temperature ferroelectricity in SrTiO_3_ and spacing-dependent coupling between the layers. Closely in 2004, Haeni *et al.* [182] provided an alternative way to adjust *T_c_* of ferroelectric films through strain, differing from the traditional way of chemical substitution. Strain-induced enhancement in *T_c_* of hundreds of degrees had been reported with room-temperature ferroelectricity in SrTiO_3_ being produced, which is of great use to device applications. Importantly, Choi *et al.* [66] demonstrated that the ferroelectric properties of heteroepitaxial BaTiO_3_ thin films could be markedly enhanced through strain engineering, giving rise to a broad range of operating temperatures as well as higher remanent polarization for improved noise immunity and the ability to scale FeRAM to smaller cell sizes. Over the coming years, more and more ferroelectric thin films had involved in these kinds of researches. For example, in 2007, Gariglio *et al.* [291] investigated strain relaxation and the ferroelectric critical temperature of Pb(Zr_0.2_Ti_0.8_)O_3_ thin films epitaxially fabricated on metallic 0.5% Nb-doped SrTiO_3_ substrates. Their detailed X-ray diffraction studies revealed that strain relaxation progressively occurs via misfit dislocations as the film thickness increases from fully coherent film (below 150 Å) to essentially relaxed films (above 800 Å), without modification of *T_c_* that was much higher than their homologous bulk materials. Then the lattice symmetry mismatch at interface had been experimentally manifested, which played an important role in determining the structural properties of perovskite films. In 2011, Chang *et al.* [292] fabricated epitaxial SRO films on STO (001) substrates with SRO layer thicknesses between 10 and 200 pseudocubic unit cells, and found that the orthorhombic films underwent a structural transition to the tetragonal phase with increasing temperature. Chaudhuri *et al.* [293] reported that a ferroelectric rhombohedral phase could be stabilized in thin epitaxial PbZrO_3_ films experiencing large interfacial compressive stress at room-temperature. 

Moreover, dislocations derive from defects also have significant effects. The most prominent and voluminous defects in planar thin film heterostructures are the two interfaces, which not only break the crystal symmetry, but also are accompanied by physical and chemical reconstructions, bound charges, space charges and strains, thereby can determine the characteristics of ferroelectric switching [294]. Depend on results of high-resolution TEM, local strain field is associated with each dislocation, which arises from the vicinity of a defect core and can be greater than the macroscopic misfit strain [295]. High defects density may result in overlapping strain fields in neighboring dislocations [295]. A broadening of the phase transition has been shown along with an overall decrease in dielectric constant as the density of these dislocations increases, demonstrating the importance of this defects induced dislocation [251].

Recently, more intensive works have been established on the study of strain effect. In contrast to the results of the former researches that epitaxial strain is considered as a powerful means to enhance the ferroelectric Curie temperature in BaTiO_3_, an unanticipated strain-driven decrease of *T_c_* has been reported in multiferroic BiFeO_3_ thin films, which is ascribed to the peculiar competition between polar and antiferrodistortive instabilities. Study results have suggested that epitaxial strain predominantly acts on the antiferrodistortive rotations of the FeO_6_ octahedra rather than on polarization in BFO, along with the strong decrease of *T_c_* with compressive strain [294]. Furthermore, ability to tune ferroic transitions without epitaxial approaches has been demonstrated through liquid phase assisted growth, with the acquired BTO films exhibiting 0.15% residual differential thermal expansion mismatch strain, and a shift to the paraelectric-ferroelectric phase transition of 50K [295].

#### 4.1.2. Effects of Electrodes

Since ferroelectric thin films are usually made into a capacitor with the structure of electrode/film/electrode in practical applications, the effect of electrodes must be taken into account. Incomplete compensation of the polarization charges happens due to the finite screening at ferroelectric/electrode interfaces, inducing a depolarization field *E_d_* against the polarization inside the ferroelectric. Depolarization field exists in all real ferroelectric thin films and has been known as size dependent [251]. Its effect is larger in thinner films, and could affect the ferroelectric transition temperature, critical thickness, coercive field, phase transition order, and domain structure, *etc*. As a result, electrode properties are important factors in determination of the critical thickness and domain structure of ultrathin ferroelectric films as well as reliability problems of numerous ferroelectric devices [252,296,297,298]. Theoretical results have shown that the critical properties of ferroelectric thin films are not only functions of the ambient temperature, misfit strain and electromechanical boundary conditions, but also can be adjusted by tuning the characteristics of electrodes, surfaces and interfaces, through the incomplete charge compensation, near-surface variation of polarization and work function steps of ferroelectric–electrode interfaces [299].

Theoretical researches began to model the finite screening effect of electrodes in 1970s, which had provided an instructive guideline for experimental researches. In the 1990s, experimental studies had been carried out on the electrode-induced ferroelectricity suppression on thin film capacitors, with the inhibition at the electrode/ferroelectric interface being discussed and new types of electrode being developed for polarization fatigue-free capacitors [300,301,302,303]. In 1999, the internal screening of the ferroelectric polarization promoted by near-by-electrode injection was shown to play a decisive role in the size effect on switching in ferroelectric thin film capacitors by Tagantsev *et al.* [304]. Their electrical measurements on sol–gel derived Pb(Zr, Ti)O_3_ thin films also implied a possibility of influencing the switching properties of ferroelectric thin films by modifying the conductive properties of the ferroelectric/electrode interface. Since advances in AFM techniques, electrode effects on ferroelectric thin films had attracted increasing interests. For instance, in 2002, Roelofs *et al.* [212] probed the switching of in-plane polarization inside domains and the preferential formation of reversed 180° domains at 90° domain walls in polydomain epitaxial Pb(Zr_0.2_Ti_0.8_)O_3_ thin films via PFM, which could be well explained by the effects of the depolarizing fields caused by transient polarization charges appearing on the domain walls. 

Afterwards, studies on electrode effects had been intensively pursued through a combination of experimental studies and first-principles calculations. Here we would pay attention mainly to experimental progresses. In 2005, Kim *et al.* [256] carried out investigation on high-quality ultrathin SrRuO_3_/BaTiO_3_/SrRuO_3_ capacitors and demonstrated that the depolarization field inside ferroelectric films could cause a severe polarization relaxation which could pose a serious size limitation for ultrathin ferroelectric devices. They also revealed that the depolarization field originated from intrinsic properties of electrode material such as the finite screening length and that the depolarization field should play an important role in domain dynamics in ultrathin ferroelectric films. Later, Jia *et al.* [284] observed a systematic reduction of the atomic displacements at the substrate/film interfaces, and considered that the electrical boundary conditions played a prominent role in the stabilization of the ferroelectric polarization for films with a thickness of a few lattice unit cells. Hereto, interfacial ‘‘dead layers’’ was thought to be responsible for the orders of magnitude collapse in the polarizability of thin film-capacitor structures. Soon after in 2008, Chang *et al.* [305] found that the functional properties of the single crystal BaTiO_3_ thin film lamellae are comparable to bulk BaTiO_3_, adding confidence to the view that dead-layer parasitic capacitances are not a general intrinsic feature of thin film ferroelectric behavior. Later they [306] established analogous experiments on SrTiO_3_ isolated single crystal thin film lamellae. The results, however, demonstrated that inherent dead layers occur in SrTiO_3_ thin films with platinum electrodes, leading to significant suppression in permittivity. Therefore, they came up with the point that dielectric dead layers were an inherent feature of many dielectric-metal interfaces but could be engineered out in specific cases where there was particularly weak interface bonding, which to some extent solves the conflict between experiment and theory.

**Figure 9:**
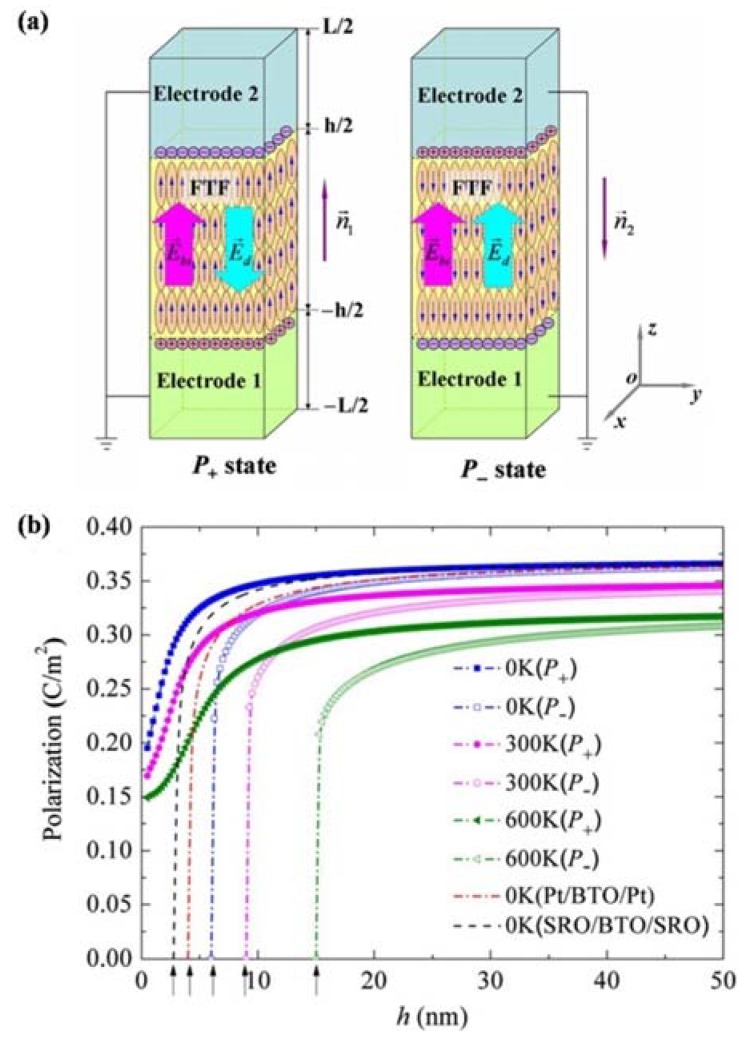
(**a**) Schematic configuration of an asymmetric-FTJ (A-FTJ) or asymmetric ferroelectric capacitor (A-FC) with 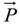 = *P*_+_ || 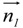 state (left), and *P* = *P*_-_ || 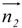 state (right). Since the asymmetric electrodes break the inversion symmetry of FC, the total free energy is certainly determined by the directions of polarization. Thus the spontaneous polarization 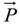 can be expressed as *P*_+_ || 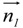, and *P*_-_ || 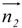, in which 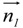 and 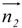 are the unit vectors pointing to the positive and negative z direction; (**b**) polarizations as functions of the ferroelectric thin film (FTF) thickness *h* of BTO A-FC, with arrows depicting the critical thickness [311].

Recently, with advances in deposition techniques, oxide electrodes have been taken to a new application level with relevance to the structural quality of oxide heterostructures. Such heterostructures have exhibited a wealth of phenomena at the boundaries where compounds with different structural instabilities and electronic properties meet, giving unprecedented access to new physics emerging at oxide interfaces (see, e.g., a review by Zubko *et al.* [307]). Moreover, attempts have been done to the fabrication of artificial multifunctional materials through well controlling of these electrode effects. It is noteworthy that recent phenomenology and first-principle calculations have demonstrated that asymmetric electrodes could lead to distinguished ferroelectric size effect, imprint behaviors, and novel properties (e.g., dielectric behavior and electron transport) in ferroelectric thin films from their symmetric counterparts, such as smearing of the phase transition, an induced piezoelectric response above *T_c_*, and reversal of the polarization asymmetry by application of biaxial strain *etc.*, which should be further verified experimentally (e.g., via x-ray diffraction analysis) [252,299,308,309,310,311,312,313]. To be more clear, we would like to take the theoretical research of Zheng *et al.* [311] as an example, where asymmetric ferroelectric capacitors (A-FCs) with a single-domain ferroelectric thin film (FTF) sandwiched between different electrodes has been investigated (Figure 9a). They revealed two important behaviors of A-FCs, *i.e.*, vanishing critical thickness for the spontaneous polarization and smearing of the phase transitions. According to the results (see Figure 9b), critical properties are highly sensitive to the structures of asymmetric interfaces and electrodes, suggesting that the critical and other functional properties of nanoscale asymmetric ferroelectric tunnel junctions or capacitors can be completely controlled by adjusting the difference between asymmetric interfaces or electrodes.

To sum up, depolarization field, which exists in all real FTFs and is electrode-dependent, can significantly affect the polarization in FTFs and usually suppress ferroelectricity, resulting in a critical thickness. Meanwhile, some other factors such as ambient temperature, misfit strain and electromechanical boundary conditions, *etc.*, also have considerable influence on critical properties of FTFs. Therefore, proper interact-adjusting of the factors may tune critical properties (including critical thickness) of FTFs as what we need, which calls for intensive researches in experiment. For instance, in experimentally epitaxial FTFs, misfit strain can enhance film ferroelectricity and weaken the impact of depolarization field, thus decrease critical thickness.

### 4.2. Electromechanical Behavior

Being a phenomenon originated from the collective distortion of lattice unitcells, ferroelectric polarization is intrinsically coupled with mechanical deformation. Consequently, ferroelectrics exhibit a mechanical response to uniform electrical inputs as well as an electrical response to uniform mechanical inputs, namely electrostriction and piezoelectricity, giving rise to potential applications in sensors and actuators of electromechanical systems. Moreover, the coupling between polarization and a strain gradient leads to a new electromechanical behavior known as flexoelectric effect, based on which mechanical force could be used as a dynamic tool for polarization control and may enable applications in which memory bits are written mechanically [314]. In this section, these two electromechanical behaviors (piezoelectric and flexoelectric effect) in ferroelectric thin films will be generally introduced along with the research progresses of this field. It is worth pointing out that recent introduction of flexible substrates (see details in Section 2.2) has offered a particularly attractive and effective way for the study of thin-film electromechanical behaviors, which breaks the limits of film brittle nature and very small mechanical deformations. Inorganic and organic ferroelectric thin films intimately integrated with flexible substrates can both exhibit excellent stretchable properties and maintain ferroelectric or piezoelectric properties without significant loss, which not only provides application possibilities in piezo-related devices, but also makes it possible to carry out visualized research on piezoelectric and flexoelectric effects.

#### 4.2.1. Piezoelectric Effect

A ferroelectric thin film can exhibit large piezoelectricity after electrical poling, even if many domain walls are presented. Ferroelectric thin films in polycrystalline form are often superior to those of single crystal in adjustability of properties, thus usually with a very large range of properties can be acquired. Piezoelectric effect in ferroelectric films has drawn great attentions since its discovery. Increasing interest in the piezoelectric properties of ferroelectric thin films stems from the attractive high piezoelectric response in systems, which has offered promising microelectronic and micromechanical applications. 

Pb(Zr, Ti)O_3_ films with compositions near the morphotropic phase boundary had been reported and considered as the best candidates in 1990s, due to their enhanced piezoelectric behavior [315] (see a review by Damjanovic [190]). To obtain the maximum voltage response and to increase the operating frequency, Ti-rich compositions that offered lower dielectric constants were desired. For more excellent piezoelectric properties and coupling factors after poling, ion doping of lead-based titanate had been extensively investigated, but no experimental data were available on the piezoelectric coefficients of these materials in thin film form until Calzada *et al.* [316] experimentally investigated effective piezoelectric coefficient d_33_ and strain in Ca-modified PbTiO_3_ films. The films could be efficiently poled even at room temperature under a field of 300 kV/cm and showed an effective d_33_ which is agree well with the piezoelectric coefficients observed in bulk counterparts. The achieved electric field-induced strain under unipolar driving showed a good linearity and small hysteresis that was a useful property for micromechanical applications. 

Later, intensive studies of piezoelectric effect in ferroelectric thin films had been established on extending fields such as characterization of electromechanical properties via probing methods like PFM [317,318] and laser interferometry [319], integration and application study mainly for micro-sensors and actuators [320], lucubrate study for effective piezoelectric coefficient [321], *etc*. Within a few years of research, tremendous progress had been achieved in understanding mechanisms of piezoelectric behavior in ferroelectrics. Considerable efforts were made to understand details of extrinsic contributions to the piezoelectric effect, focusing on the discovery of enhanced piezoelectric response along nonpolar directions in perovskite crystals. For example, effective piezoelectric coefficient *d*_33_ was found strongly related to film thickness [317]. Bühlmann *et al.* [322] had revealed a strong increase of the piezoelectric response in Pb(Zr_0.4_T_0.6_)O_3_ thin films on conductive Nb-doped SrTiO_3_ (100) substrate, which was proposed mainly due to vanishing a domains. Moreover, domain-wall contributions had been put forward to be a significant reason for variation in the piezoelectric nonlinearity with film orientation [321]. In 2003, conclusive evidence had been experimentally presented by Nagarajan *et al.* [216] for significant contributions from ferroelastic 90° domain movement to the piezoelectric responses of epitaxial PZT films when the clamping imposed by the substrate was reduced. Besides, size effect on piezoelectric properties of PZT thin film also have been investigated, revealing that piezoelectric properties are as a function of film thickness and to some extent influenced by the critical thickness. Effects of many factors, e.g., orientation, composition, grain size, defect chemistry, and mechanical boundary conditions, on the observed piezoelectric coefficients in PZT films have been discussed in a comprehensive review [323]. 

Although PZT demonstrated high-performance piezoelectric behavior, efforts have been also thrown on developing new kinds of lead-free materials due to concerns regarding the toxicity of lead. In 2004, Saito *et al.* [324] reported a lead-free piezoceramic with an electric-field-induced strain comparable to typical PZT. They designed a compositional formation of morphotropic phase boundary (MPB) between a different pair of crystal structures. Progress has been made during the following years. For instance, in 2009, Ahn *et al.* [325] fabricated K_0.5_Na_0.5_NbO_3_ (KNN) thin films on Pt substrates by a CSD method and investigated the effect of K and Na excess (0–30 mol %) on ferroelectric and piezoelectric properties of KNN thin film. They indicated that the optimized KNN thin film had good fatigue resistance and a piezoelectric constant of 40 pm V^−1^, which was comparable to that of polycrystalline PZT thin films. Later on, as the gradually expanding field of piezoelectric researches, a variety of lead-free ferroelectric thin films with considerable piezoelectric properties have been reported. BNT-based thin films are reported possessing impressive transverse piezoelectric coefficient (e_31,f_) and effective piezoelectric coefficient (d_33_), thus are considered as a potential candidate for lead-free piezoelectric devices [326,327]. Ferroelectric BiFeO_3_ thin films also have been an attractive alternative because of it is lead-free, as well as the advantages that BiFeO_3_ phases can be stabilized with a structure resembling a morphotropic phase boundary via strain tuning [294,328,329]. Zhang *et al.* [330] reported a reversible electric-field-induced strain of over 5% in BiFeO_3_ films. More recently, very large piezoelectricity in lead-free nanostructures sintered as thin films and nanofibers have been reported by Jalalian *et al.* [331]. Additionally, some polymeric nanostructured ferroelectric-related materials such as PVDF nanowires also have been reported to have enhanced piezoelectric properties with outstanding piezoelectric effect [332], which is promising in practical applications.

#### 4.2.2. Flexoelectric Effect

Term “flexoelectricity” is firstly introduced by Kogan *et al.* [333] who discussed the electric polarization induced in a symmetric crystal by inhomogeneous deformation. Flexoelectricity is afterwards regarded as a kind of electromechanical coupling effect between electric polarization and mechanical strain gradient (*i.e.*, direct flexoelectric effect) or vice versa between electric polarization gradient and mechanical gradient (*i.e.*, converse flexoelectric effect), which exists in various categories of materials including solid materials, liquid crystals, polymers, and biomembranes [334]. Since flexoelectric effect is caused by inhomogeneous strains, flexoelectricity does not require crystalline structures with no inversion symmetry, which is quite different from piezoelectricity [335,336]. Recently, an increasing amount of attention has been paid to flexoelectricity in ferroelectric thin films. Here we make a brief introduction about this topic.

Experimental observation of large flexoelectricity in ferroelectric ceramics and thin films was reported in this century, when Ma and Cross carried out temperature-dependent measurement of the flexoelectric coefficients in a series of ferroelectric ceramics including Pb(Mg_1/3_Nb_2/3_)O_3_ (PMN) [337], Ba_0.67_Sr_0.33_TiO_3_ (BST) [338], PZT [339], and BTO [340], *etc*. The large flexoelectric coefficients in ferroelectric ceramics suggested that strain gradients could affect the polarization and permittivity behavior of ferroelectrics, from which flexoelectric effect was deduced to have a significant impact on epitaxial ferroelectric thin films and mesoscopic structures. In 2005, Catalan *et al.* [341] revealed the presence of strain gradients across Ba_1/2_Sr_1/2_TiO_3_ ferroelectric thin films with different thicknesses via X-ray analysis, which showed that strain relaxation in ferroelectric thin films could lead to smooth and continuous strain gradients across hundreds of nanometers, highlighting that the pressing need to avoid such strain gradients in order to obtain ferroelectric films with bulklike properties. Experimental data had already suggested that flexoelectric effect could make an important contribution to the properties of epitaxial thin films where mismatch could give rise to very steep elastic strain gradients. Thus it could be surmised that exceedingly high strain gradients might be presented around local crystalline defects such as oxygen vacancies and dislocations. Soon after, Zubko *et al.* [342] measured polarization induced by bending in single crystals of SrTiO_3_ as a function of temperature with different crystallographic orientations allowing all components of the flexoelectric tensor in the order of 10^−9^–10^−8^ C/m to be estimated, which were suggested to be produced around dislocations or defects.

**Figure 10:**
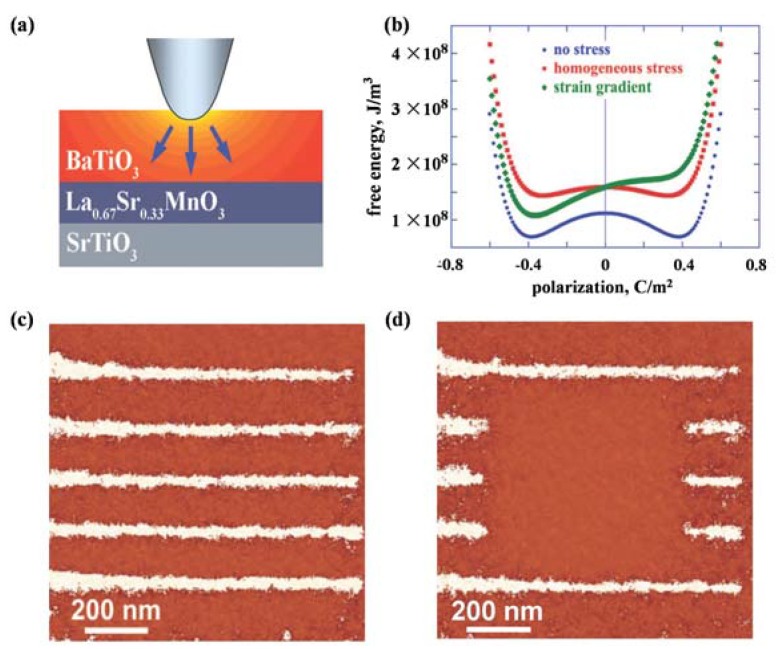
(**a**) Sketch of the strain gradient and associated flexoelectric field (arrows) induced by the AFM tip pushing on the surface of the BTO/LSMN heterostructure; (**b**) free-energy calculations for the epitaxially clamped BTO film without any tip pressure (blue curve), with homogeneous compressive stress of 3.2 GPa (red curve), and with the calculated flexoelectricity from the tip-induced strain gradient (green curve), where flexoelectricity skews the double well, forcing polarization switching toward the stable downward state; (**c**) domain lines mechanically written in the BTO film by scanning the film with a piezoresponse force microscopy (PFM) tip under a loading force of 1500 nN; (**d**) the same domain structure modified by electrical erasure of the mechanically written domains, which is performed by scanning the central segment with the tip under a dc −3-V bias [314].

With increasing interest in nanoscale and materials, flexoelectricity continued to gain prominence. In 2011, Catalan *et al.* [343] experimentally investigated flexoelectric rotation of polarization in PbTiO_3_ ferroelectric thin films through synchrotron XRD and high-resolution STM, which revealed an intricate strain distribution with gradients in both the vertical and unexpectedly the horizontal direction. They suggested the gradients to generate a horizontal flexoelectricity that forced the spontaneous polarization to rotate away from the normal, providing an alternative route to induce polar rotations in non-morphotropic ferroelectrics using purely physical means. Later on, further study on flexoelectric-related mechanical writing of ferroelectric polarization had been explored on epitaxial single-crystalline BaTiO_3_ thin films by Lu *et al.* [314] in 2012. The stress gradient generated by the tip of AFM was demonstrated with the ability of mechanically switching the polarization in nanoscale volume of a ferroelectric film (see Figure 10), providing a promising concept that pure mechanical force could be used as a dynamic tool for polarization control, thus should enable applications where memory bits are written mechanically and read electrically. Furthermore, mechanical writing on films with top electrodes would enable the targeted poling of localized areas under the electrodes with inhomogeneous electric field which cannot be achieved through applied voltage, leading to the possibility of controlled fabrication of domain walls underneath top electrodes. 

Additionally, as a size-dependent effect, flexoelectricity is more significant in nanoscale systems. The influence of flexoelectric coupling is expected to intensify at smaller dimensions in which flexoelectricity associated field and stress will be extraordinarily large. In contrast, the size of the gradients is inversely proportional to the domain size, which can be tuned by changing the film thickness or the film–substrate combination [343,344]. Moreover, flexoelectric coefficients are thought to be larger for lead-free ferroelectrics than for lead-containing ones, positively motivating in the quest for advanced lead-free materials with large piezoelectricity [345]. So far, considerable giant flexoelectric effect have been reported in many ferroelectric epitaxial thin films, which are usually six or seven orders of magnitude larger than those in conventional bulk solids, tremendously affecting film properties [346,347]. Fruitful experimental and theoretical results have revealed that flexoelectric effects are sensitive to chemical makeup, phase symmetry, and domain structures, which can make a significant impact on film properties through coupling effect with other properties in thin films such as electric (for example resistive switching), magnetic as well as ferroelectric properties, giving rise to new phenomena and considerable application potential (e.g., ferroelectric domain writing and erasing, control of film transparent properties, modifying piezoelectric properties, and modulation of interfacial barrier height, *etc.*) [334,348,349,350,351].

### 4.3. Domain Structure

Formation of domain structure is a general feature of ferroelectrics. Domain structures in ferroelectric thin films have been well-known to have important and even crucial impact on ferroelectric behaviors such as polarization switching, phase transition, critical properties and electromechanical properties, *etc.*, thus play important roles in device applications. During the past decades, on the basis of fast development of domain characterizing techniques and to meet the expanding demand for obtaining direct and complete information about domain kinetics, large amount of experimental researches have been carried out, with experimental real-time registration of domain patterns during switching being successfully achieved (see Section 3 in review). Tremendous progress has been made in this area. With an ever-expanding demand for application, increasing experimental highlights have been shed on domain structure evolution in ferroelectric thin films, as well as the properties mediated by domain and domain walls. Important and impressive experimental works in these two aspects will be generally reviewed and discussed as follows.

#### 4.3.1. Domain Structure Evolution

At the ferroelectric phase, ferroelectric thin films generally consist of small regions of different polarity, which are the so-called “domains”, with the boundaries between adjacent domains being named “domain walls”. The equilibrium domain configuration is determined by the minimum of the overall energy of the system. For an epitaxial ferroelectric thin film, strain relaxation at the substrate/film interfaces and the depolarizing field caused by electrode screening are considered as important factors to equilibrium domain configuration [352]. The critical factors controlling the domain structure, such as substrate, film composition, film thickness, introduction of an interlayer, and the lateral size of film patterning, have been described in detail by Lee and Baik [353]. 

**Figure 11:**
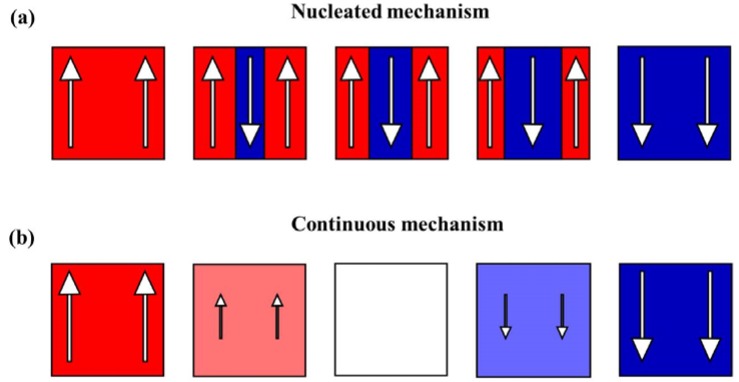
Schematic of polarization evolution during switching in a uniaxial ferroelectric: (**a**) discontinuous nucleation and growth of inverted domains (explained by Kolmogorov-Avrami-Ishibashi (KAI) mechanism); (**b**) continuous, uniform decrease of the polarization magnitude through zero without domain formation (explained by Landau-Ginzburg-Devonshire (LGD) mechanism). Arrows indicate the orientation and magnitude of polarization [203].

Conventionally, domain orientation can be switched under an applied external electric field [200]. Domain switching may proceed via nucleation mode or continuous mode as illustrated in Figure 11 [203]. Domains can only be switched by an electric field greater than a threshold called the coercive field *E*_c_. In principle, the coercive field *E*_c_ of domain switching (extrinsic switching) can be no larger than the intrinsic coercive field *E*_c0_ of polarization switching (a homogenous switching or intrinsic switching) as predicted by Landau-Ginzburg-Devonshire (LGD) mean-field theory [276,354], at which the initial polarization state becomes unstable and is switched to the new direction with no energy barrier (shown in Figure 11b). When the applied fields are below *E*_c0_, the initial state is only metastable rather than unstable. Nevertheless, domain switching is still possible via thermally activated nucleation of domains of the new orientation (shown in Figure 11a).

In literature, domain switching kinetics at the nanoscale is an important topic of both theoretical and experimental investigations [203,277,355,356]. Domain switching kinetics of ferroelectric crystals and thin films has been explained by the domain nucleation and growth theories [191,357]. The domain switching theory of Kolmogorov-Avrami-Ishibashi (KAI) successfully explained the switching kinetics of ferroelectric crystals and even thin films with thickness *l* ≥50 nm [358]. In the simplest case, KAI switching is dominated by a nucleation-limited process, where reciprocal switching time τ has an exponential dependence on temperature *T* and reciprocal electric field *E* (or voltage V), as follows,

τ^-1^ ∝ exp(−α / *E*) (3)

Where the activation field α typically has a reciprocal dependence on the temperature *T*. Note that there is no true coercive field Ec because as the switching field is reduced, switching becomes progressively slower. Thus the extrinsic coercive field obtained from P-E hysteresis loops for the bulk crystals and films does not limit the switching rate. It is the initiation by nucleation that gives extrinsic switching its essential threshold-less character, although other factors such as finite domain wall velocity and pinning can give similar character. [355]

For ultrathin ferroelectric films with thickness of 1–10 nm [280,281,359,360], the realization of KAI mechanism by three-dimensional nucleus is hardly possible, resulting in the switching mechanism of such ultrathin films could be homogeneous [277]. Contrary to the domain nucleation and growth kinetics (inhomogeneous switching or extrinsic switching), the kinetics theory of homogeneous switching (intrinsic switching), as predicted by the Landau-Ginzburg-Devonshire (LGD) theory, does have a true threshold voltage and exhibits critical behavior. The dependence of the switching rate τ^−1^ on the applied electric field has the form near the intrinsic coercive field *E*_c0_ as follows,

τ^1^ ∝ (*E* / *E*_c0_ -1)^1/2^ (4)

where *E*_c0_ decreases steadily with increasing temperature. Note that switching is only possible at the field above intrinsic coercive field. Since the intrinsic coercive field in bulk crystals or thick films is much higher than the typical extrinsic coercive field of most samples, intrinsic switching is generally not observable. It is only possible to observe intrinsic switching in thin films or nanostructures that are smaller than the minimum critical nucleation size (estimated about 10 nm). [355]

So far, both extrinsic switching and intrinsic switching have been experimentally observed, with the theories mentioned above being confirmed. Besides the first reported observation of intrinsic switching which being made with the ferroelectric copolymer Langmuir-Blodgett (LB) films [361], ultrathin (~10 nm) epitaxial ferroelectric films of PZT [362], PTO [203], and BTO [355] have also been successfully involved in observation of intrinsic switching. Moreover, extrinsic switching has been reported in a 54-nm thick P(VDF-TrFE) LB film, whereas an 18-nm thick film shows intrinsic switching, demonstrating a critical size for intrinsic switching [363]. In conclusion, there is a competition of extrinsic (domain–driven, KAI) and intrinsic (LGD) mechanisms at the nanoscale. For the thickness of the ferroelectric film comparable with critical domain size (1–10 nm) [191], the intrinsic mechanism prevails, where domain switching could be homogeneous and described by LGD theory. Conversely, when the nucleation-limited mechanism prevails, it would result in inhomogeneous switching which can be described by KAI theory. This competition can be regarded as the main feature of ultrathin ferroelectric films, which may probably determinate all properties of films including switching, hysteresis, and scaling (critical thickness), *etc.* [277].

Many other factors have also been demonstrated to be connected with domain evolution. Li *et al.* [364] demonstrated that a substrate constraint results in sequential nucleation and growth of different tetragonal domains during a ferroelectric phase transition. Because of any ferroelectrics were also ferroelastics with hysteresis in their stress-strain relationships, resulting in an intimate interplay between the ferroelectric and ferroelastic domain configurations. In continuous thin films, non-180° domain maybe clamped by the substrate, Nagarajan *et al.* [216] provide a method to significantly reduce the clamping effect by patterning ferroelectric layer into discrete islands using a focused ion beam, by which to obviously facilitate the movement of ferroelastic walls. Depolarizing-field-mediated 180° switching in ferroelectric thin films with 90° domains also had been reported by Roelofs *et al.* [212], where the depolarizing fields caused by transient polarization charges appearing on these domain walls were regarded as the reason of the switching of in-plane polarization inside a domains as well as the preferential formation of reversed 180° domains at 90° domain walls.

Since the observation of electrical control of antiferromagnetic domain structure in a single-phase multiferroic BiFeO_3_ films at room temperature [217], nanoscale control of domain architectures and evolution processes have attracting increasingly interests. Ion doping has been considered as an important factor for complex domain-related structure transitions in ferroelectric thin films according to the study of Cheng *et al.* [365]. They observed a series of phase transitions and superstructure with continued additions of Sm^3+^ doping in BiFeO_3_ thin films, owing to antiparallel cation displacements in local pockets created by Sm^3+^ doping. In the same year, Chu *et al.* [366] demonstrated an elegant approach to control the domain variants in epitaxial La-substituted BiFeO_3_ films to obtain one-dimensional and quasi-periodic nanoscale arrays of domain walls. In their experimental study, DyScO_3_ (110) single-crystal substrate together with the use of a SRO layer to break the elastic and electrostatic boundary conditions, realizing the flexible choice of obtaining either 109° or 71° one-dimensional periodic domain walls in La-doped BFO thin films.

**Figure 12:**
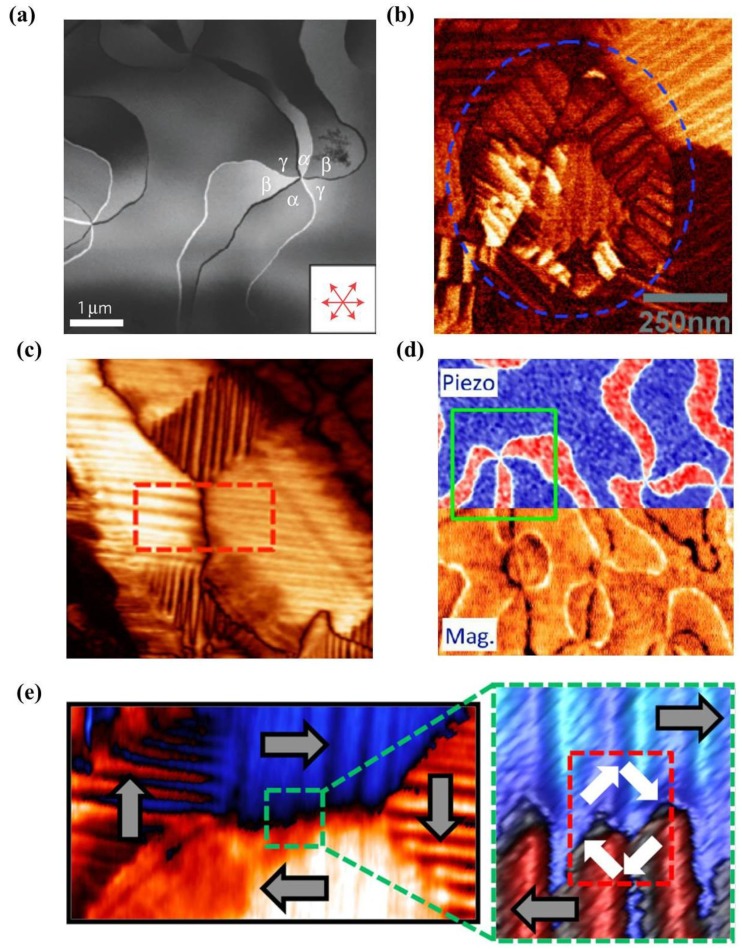
(**a**) TEM dark-field image of cloverleaf domain patterns in ferroelectric YMnO_3_, showing six antiphase domains (α−β−γ−α−β−γ) emerging from one central point (cited from the paper of Choi *et al.* [371]); (**b**) enhanced piezoresponse force microscopy (EPFM) imaging of a native vortexlike 90° domain structure within a macroscopic polarization vortex in polycrystalline PZT thin films (taken by Ivry *et al.* [372]); (**c**) PFM amplitude image of domain structures at the edges of the PZNPT single crystal lamella, which are found to consist of blocks or bundles of a_1_-a_2_ 90° stripe domains (taken by Chang *et al.* [373]); (**d**) room-temperature PFM image and MFM image taken at the same location on the (001) surface of a hexagonal ErMnO_3_ single crystal (completed by Geng *et al.* [374]); (**e**) domain structures at the edges of the PZNPT single crystal lamella, which are found to consist of blocks or bundles of a_1_-a_2_ 90° stripe domains (mapped by Chang *et al.* [373]).

Recently, improper ferroelectrics result from other phase transitions such as magnetic orders, charge orders or structural transitions owing to zone-boundary instability, have attracted increasing attention [367,368]. Since recent identification that topological defects can play important roles in phase transition in hexagonal manganites, topological defects quickly become a current focus, especially in hexagonal REMnO_3_ (RE refers to rare earth, which maybe Ho–Lu, Y and Sc *etc.*) [369,370]. Domain walls in hexagonal are considered as topologically protected, detailed nature of these ferroelectric domains has been vastly investigated lately. Choi *et al.* [371] have observed a unique ferroelectric domain pattern with a cloverleaf shape in hexagonal REMnO_3_ (shown in Figure 12a), where ferroelectricity is driven by a trimerization-type structural instability, providing a new paradigm for the scientific and technological issues of coupling among structural domain boundaries, ferroelectric domain walls, magnetic domain walls and charge carriers in REMnO_3_. 

Moreover, novel toroidal domain structures in ferroelectrics, which is a kind of topological domains (called vortex domain structure), have attracted much attention. Han *et al.* [375] have established significant experiment on ferroelectric switching dynamics of topological vortex domains, which determined and illustrated that topological defects orchestrate the domain switching process in hexagonal manganite (ErMnO_3_). For vortex domain structure in ferroelectric thin films, the study of Ivry *et al.* [372] (see Figure 12b) showed that vortexlike 90° domain structures were not unique to nanodots and could also be found in polycrystalline films, with two different flux closure configurations of a quadrant azimuthal structure and a radial structure being observed in ferroelectric PZT thin films. When applying an external electric field, the vortexlike structure was induced far away from a grain boundary, indicating that physical edges were not necessary for nucleation contrary to previous suggestions. Subsequently, despite the intensive studies of vortex domains on nanorods, many works have been lucubrated into complex vortex behavior in ferroelectric thin films. Recently in 2013, Chang *et al.* [373] have mapped an interesting topological polarization object spontaneously formed at the edge of a [Pb(Zn_1/3_Nb_2/3_)O_3_]_0.88_[PbTiO_3_]_0.12_ (PZN-12PT) lamella on cooling through the Curie temperature with distinct core singularities at the center of flux-closure arrangements being suggested energetically unfavorable (see Figure 12c,e). The discovery of the “nested” topological structure can be of great importance in extending the knowledge on the possible domain patterns that can form spontaneously in response to the significant depolarizing fields, presenting in both meso- and nano-scale ferroelectric objects. 

#### 4.3.2. Domain and Domain Wall Mediated Properties

Many properties in ferroelectric thin films are mediated by domains and domain walls. As intrinsically nanometer scale interfaces for separation of different orientations in spontaneous electric polarization, the naturally occurring ferroelectric domain walls can present radically different properties from their parent materials. Density of mobile domain walls is considered to be one of the important factors to facilitate the influence of domains which may cause a series of phenomena in ferroelectric thin films such as dramatically enhance the susceptibility of ferroelectricity, and even initiate changes in magnetic susceptibility, elastic compliance, dielectric constant or piezoelectric coefficient, *etc*.

Domain wall have been found to exhibit attractive electrical transport properties, which have been intensively investigated and more detailed information about recent progress on this aspect will be provided in Section 4.4.4. Apart from the popular properties of domain wall conduction, many properties related to domains and domain walls have been investigated. Domain wall, which can host properties absent in the bulk (e.g., they can be super-/semi-conductive and even magnetic), can carry information and act as memory devices. Domain wall width is suggested significant to its storage properties, which has been verified to have interaction with point defects around it. According to the study of Salje *et al.* [376], interaction between the order parameter and the point defect concentration caused point defects to accumulate within twin walls, resulting in width of twin walls in PbTiO_3_ being strongly broadened. The present defects is probably tend to migrate towards and may pin the domain walls, leading to a mechanism for the domain wall to have a memory of its location during annealing and to some extent altering of polarization behavior, especially for those thin films with ultra-small thickness. Lately, defect-mediated ferroelectric domain depinning of polycrystalline BiFeO_3_ (BFO) multiferroic thin films have been investigated by Bretos *et al.* [377]. In their experiments, enhanced domain mobility was obtained for the re-oxidized BFO film with larger contribution to ferroelectric polarization, as well as the current-voltage (I-V) characteristics of the film, depending on which the possible generation of new defect dipoles upon continued electric cycling or with time, which would affect the reliability of re-oxidized BFO films, maybe an area of future investigation. Moreover, manipulation of ferroelectric domains and domain walls has been indicated to be connected with optical properties and electro-optical response of ferroelectrics, for instance, periodic poling of domains in ferroelectrics always involved in nonlinear optic properties, which has been reviewed in detail by Catalan *et al.* [224]. Commonly, the design of “optimal” domain structures should correspond to “optimal” domain walls with parameters such as the domain boundary mobility, pinning properties and the formation of specific boundaries such as curved boundaries or needle domains [376].

Some new domain mediated properties have also been reported lately. An anomalous photovoltaic effect in BFO thin film has been observed by Yang *et al.* [378], which is shown arising from structurally driven steps of the electrostatic potential at nanometer-scale domain walls, demonstrating the existence of photovoltaic effects at domain walls. In 2011, Seidel *et al.* [379] described the origin of the photovoltaic mechanism driven by the periodic potential structure formed by BFO ferroelectric domains. They demonstrated that the effect was due to charge separation at the walls, and also indicate that this kind of photovoltaic effect should not only occur in BFO, but also in any system with a similar periodic potential structure. Besides, magnetism and magnetoelectric properties of multiferroic domain walls also have drawn public attentions. For example, in the interesting study of Geng *et al.* [375] (see Figure 12d), identified unprecedented collective domain wall magnetism had been demonstrated could be controlled by external magnetic fields, which revealed alternating uncompensated Er^3+^ moments at antiphase-ferroelectric domain walls interconnected through self-organized vortex networks in multiferroic hexagonal ErMnO_3_, significantly representing a major advancement in both magnetism and ferroelectrics and paving a new way for potential multifunctional applications of cross-coupled domain walls. In addition, domain evolution has been considered to have something to do with fatigue process in ferroelectric thin films. In the experimental study of domain evolutions in (111)-oriented Pb(Zr_0.55_Ti_0.45_)O_3_ and (115)-oriented SrBi_2_Ta_2_O_9_ thin films carried out by Liu *et al.* [380], the aggravation of stress in PZT film resulted in “pinning” of domains and “freezing” of perovskite cells (90° a-c domain walls form at the beginning of fatigue process and increase in amount with accumulation of switching cycles accompanied by increase of stress) which is thought to be the origin of PZT fatigue, while nearly stress-free status of SBT film during the repeated switching cycles leads to flexibility of polarization reorientation and the fatigue-free property, suggesting the importance of domain evolution and stress in film properties during fatigue process.

### 4.4. Electronic Transport in Ferroelectric Thin Films

As an important category of oxide materials, ultrathin ferroelectric films have become a popular ingredient for new concepts of electronic devices for use as next-generation nonvolatile memories, which is to a large extent due to the profusion of new physics and novel functionalities arising from their relatively small size as well as the interplay between ferroelectric orders and electronic transport on nanoscale. Throughout current popular phenomena in ultrathin ferroelectric films, electronic transport properties have possessed a dominant position, which can be majorly summarized as giant tunnel electroresistance (GER) effect, giant piezoelectric resistance (GPR) effect, photoelectric and photodiode effect, domain wall conduction, and the burgeoning resistive switching. In this section, these newly focused phenomena will be introduced, with the research status being abstracted mainly in respect of experimental studies.

#### 4.4.1. Giant Tunnel Electroresistance Effect

**Figure 13:**
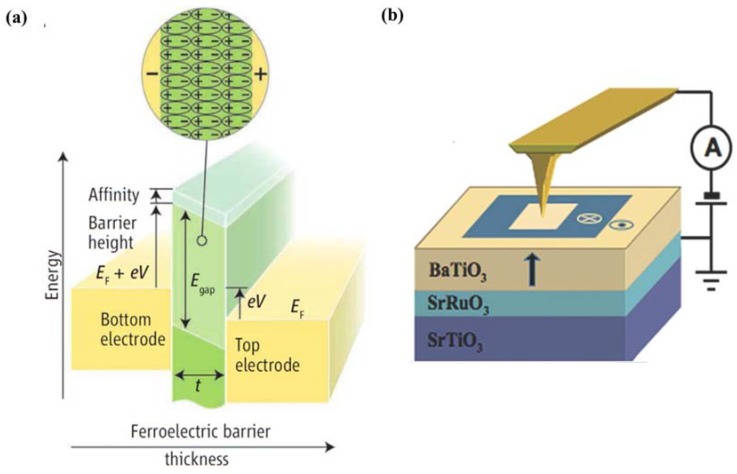
(**a**) Schematic viewgraph of a ferroelectric tunnel junction which consists of two electrodes separated by an nm-thick ferroelectric barrier layer (cited from Tsymbal *et al.* [381]); (**b**) sketch illustrating the geometry of experiment for PFM/c-AFM studies of tunneling electroresistance effect in ultrathin BaTiO_3_ films (Gruverman *et al.* [383]).

Giant tunnel electroresistance effect (GER) is an important phenomenon on the basis of electron tunneling, which has been known since the advent of quantum mechanics, drawing a considerable interest from the point of view of the fundamental science as well as device applications. When the thickness of the ferroelectric reaches a few nanometers, electron tunneling becomes possible, which may more probably happen in ultrathin ferroelectric films. A ferroelectric tunnel junction (FTJ) usually consists of two metal electrodes separated by an ultrathin ferroelectric barrier layer, which has been well illustrated through schematic diagram by Tsymbal *et al.* [381] (see Figure 13a). Asymmetry between two metal/ferroelectric interfaces will give rise to an asymmetric electrostatic potential distribution caused by the ferroelectric polarization. Thus the averaged barrier height of the tunnel barrier will change as the polarization orientation switching, resulting in potentially large changes of the tunnel current, which depends exponentially on the square root of the barrier height [249]. Hence, ferroelectric polarization reversal in a FTJ will lead to a change in resistance of the junction, which is known as the phenomenon of tunneling electroresistance (TER) effect [382], providing a simple means for the nondestructive resistive readout of the ferroelectric polarization state. As the critical size of ferroelectric thin films experimentally decrease to several nanometers, FTJs are considered useful in practical device applications.

Almost none experimental results on GER have been demonstrated since 2009, when Garcia *et al.* [250] showed direct evidence of GER in ultrathin (from 1 to 3 nm) strained BTO bare surfaces with PFM tips being the top electrode, revealing that the resistance contrast between polarization states increases exponentially with BTO thickness. GER effect has been reproducibly demonstrated through local electron transport from an AFM tip into ferroelectric thin films. At the same year and through the same technique, Gruverman *et al.* [383] also manifested GER on BTO thin films with lateral scale of about 20 nm, the sketched geometry of experiment in Figure 13b. They also demonstrated that polarization retention was not affected by conductance measurements thus allowing multiple nondestructive polarization readouts and opening a possibility for application as non-charge based logical switches in nonvolatile memory devices. Functional properties of FTJs can be extended by replacing normal metal electrodes by ferromagnetic, which makes the junctions multiferroic, thus a multiferroic tunnel junction (MFTJ) is always adopted in experimental studies, usually with LSMO as ferromagnetic layers. The interplay between ferroelectric and ferromagnetic properties of the two ferroic constituents in a MFTJ is very likely to affect the electric polarization of the ferroelectric barrier, the electronic and magnetic properties of the ferromagnet/ferroelectric interface, as well as the spin polarization of the tunneling current [381].

**Figure 14:**
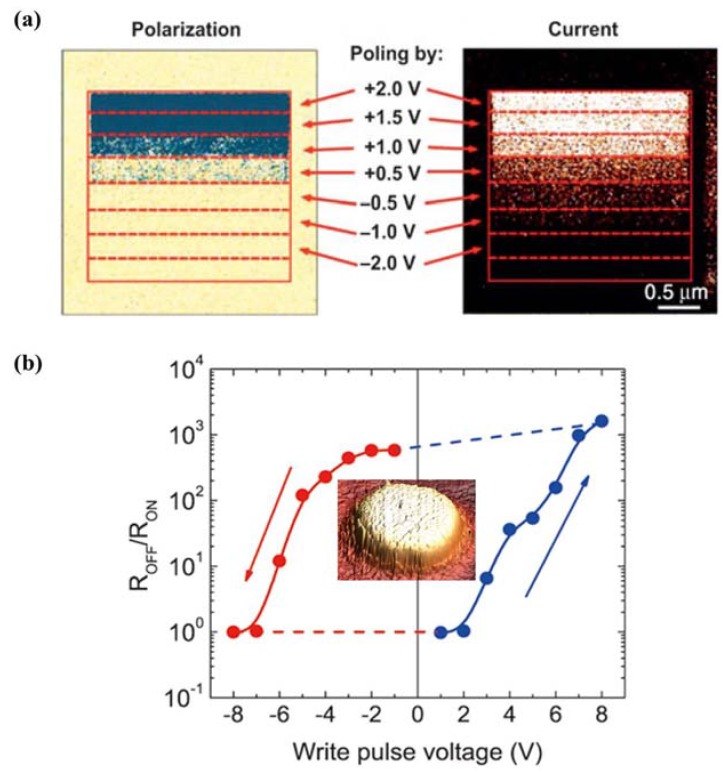
(**a**) Spatially resolved correlation between the onset of polarization reversal and a change in electrical conductance in BaTiO_3_ thin film grown on SrRuO_3_/SrTiO_3_ (Tsymbal *et al.* [382]); (**b**) R_OFF_/R_ON_ as a function of the writing pulse amplitude (pulse width is 1 ms) for the 4 u.c. thick BaTiO_3_ junctions, with topography of the junction in the center (Kim *et al.* [388]).

Later on and recently, a series of researches have been carried out to study GER effect in BaTiO_3_-based solid-state FTJs, which usually employ MFTJ structure for investigation, focusing on mechanism of GER effect, related nonvolatile storage and polarization controlling, as well as influence of different electrodes in view of interfacial coupling effect [384,385,386,387]. Some experimental result of BTO FTJs which carried out by Tsymbal *et al.* [382] and Kim *et al.* [388] have been shown in Figure 14a,b, from which obvious GER effects can be clearly seen. Additionally, research group of Wu *et al.* [386] have proposed a novel tunneling heterostructure by replacing one of the metal electrodes in a normal FTJ with a heavily doped semiconductor (*i.e.*, a metal/ferroelectric/semiconductor heterostructure, implemented in Pt/BaTiO_3_/Nb:SrTiO_3_ heterostructures). In such a FTJ, the height as well as the width of the barrier can be effectively modulated, leading to a greatly enhanced tunneling electroresistance (TER), due to the creation/elimination of an extra barrier on the semiconductor surface in response to the polarization reversal in the ferroelectric barrier.

Furthermore, roust GER effect is also reported in FTJs with PZT barriers, showing clearly the ability to control the local electron transport through a ferroelectric oxide by using small voltages in macroscopically large tunnel junctions [389,390,391]. In recent study of Yamada *et al.* [392], FTJs based on ultrathin T-type BFO films (thickness of 4 nm) have also been reported to possess GER effect, demonstrating a very large OFF/ON ratio (>10,000) in 180 nm wide ferroelectric tunnel junctions, with good retention properties reinforcing the interest in nonvolatile memories based on ferroelectric tunnel junctions. The changes in resistance are observed scale with the nucleation and growth of ferroelectric domains in the ultrathin BFO films, suggesting potential as multilevel memory cells and memristors. 

According to reports, the tunneling current can be modulated by varying the tunneling barrier height along with the polarization reversal; thereby, the resistance of a FTJ also can be controlled through polarization state, resulting in ferroresistive switching, which is promising for switchable electronic memory devices. Meanwhile, the mechanism of GER is various, including the change in electrostatic potential through incomplete screening by the electrodes, effects related to ferroelectric ionic displacement at the interfaces, the strain effect that changes the barrier width owing to the piezoelectric effect, as well as defect-mediated conduction, *etc*. Various theoretical works have been carried out on FTJs, such as the interesting change on critical thickness and ferroelectricity in ultrathin asymmetric FTJs [393]. Although considerable experimental progress have been acquired, many more attempts are in desperate need regarding the stimulation of theoretical prediction (such as FTJ with organic ferroelectric barriers of PVDF [394]), and, more importantly, regarding the necessary application. 

#### 4.4.2. Photoelectric and Photodiode Effect

**Figure 15:**
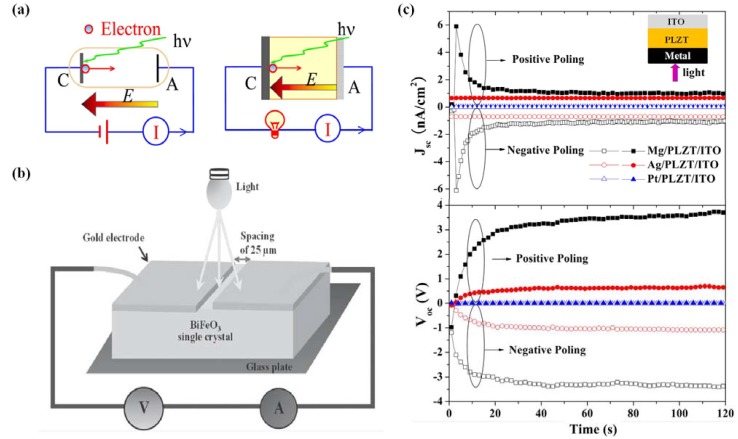
(**a**) Illustrations of a classic photoelectric diode, and a proposal ferroelectric capacitor consisting of the ferroelectric layer and the photoemission electrode respectively shown in the left and right, where A refers to anode, C refers to cathode (Zhang *et al.* [396]); (**b**) schematic illustration of the sample geometry used for the electrical measurements of BFO single crystals (carried out by Moubah *et al.* [407]); (**c**) short-circuit current density (diagram above), and open-circuit voltage (diagram below) *vs.* measuring time (from the work of Zhang *et al.* [396]).

Photovoltaic devices can convert light into electrical energy. Besides the well-known semiconductor photovoltaic devices, photovoltaic effects in ferroelectrics have been studied for decades. Commonly, two parameters are employed to evaluate the performance of a photovoltaic device including open-circuit voltage (*V_oc_*) and short-circuit current (*I_sc_*), both of which are strongly depended on the light absorption ability of active layer, the strength and space of built-in field. Bulk photovoltaic effect (BPVE) also has been found in ferroelectric materials which are often without a center of symmetry since 1950s, *i.e.*, steady-state photocurrent can exist in a homogeneous medium under uniform illumination [395]. In contrast to the traditional semiconductor, a photovoltaic effect in ferroelectrics is relied on in the intrinsic polarization-induced internal electric field instead of a p-n or Schottky junctions, which can not only improve the separation and migration of light-generated electron-hole pairs but also reduce the cost of cell fabrication, resulting in enhances of photovoltaic effects (which have been illustrated in Figure 15a [396]) [379,397]. Although ferroelectrics possess high carriers recombination rate and usually with too large bandgaps to harvest photons in the visible spectrum (*I_sc_* is very small even if under irradiation of ultraviolet (UV) irradiation), it is also appealing for their probable ability to break the limitation in conventional semiconductor-based photovoltaic devices with low *V_oc_* and narrow space-charge region of a p-n junction [398,399,400]. 

Coupled with fast developments in ferroelectric thin-film deposition, ferroelectric thin film-based photovoltaic devices have drawn increasing attentions with many researches carried out on photoelectric and photodiode effect in ferroelectric thin films such as PTO-based heterostructures [401], Pb(Ti, Zr)O_3_-based heterostructures [402], La_0.7_Sr_0.3_MnO_3_/Si p-n junctions [403], Au/Bi_4_Ti_3_O_12_/Si/Al heterostructures [404], as well as the most popular investigated BFO-based structures [405], *etc*. In the past works, some progress with respect to BPVE in thin films have been acquired, for example, remarkably higher photovoltaic efficiency has been suggested can be achieved in thin films [406], large *V_oc_* has been obtained in ferroelectric thin films with in-plane interdigital electrodes [398] *etc*. Significantly, in 2009, Choi *et al.* [245] reported that a substantial zero-bias photovoltaic current could be induced by visible light in single ferroelectric domain BFO crystals. Later, one of the most charming studies should distribute to the work of Ji *et al.* [405], they further demonstrated that the large portion of the photovoltage and photocurrent (about two thirds) in a epitaxial multiferroic BFO thin film was switchable in response to the switching of the ferroelectric polarization, with the direction of the photocurrent opposite to that of the polarization vector, indicating ferroelectric polarization as the main driving force of the observed photovoltaic effect. Importantly, the as-deposited BFO films, as reported, are self-polarized with readily function as a photovoltaic cell without any poling, which has a large obtained open-circuit voltage. Significant study also has been carried out in single domain BFO crystals by Moubah *et al.* [407] (schematically illustrated in Figure 15b), aiming at deeper understanding of the actual mechanism leading to charge separation in ferroelectrics, which have demonstrated the central role of the internal field and carrier density played in the photoelectric properties. 

More recently, to meet the expanding needs of clean and renewable solar energy, continuously attempts have been engaged in exploration for novel materials and fundamentally investigating photoelectric conversion mechanisms for the better performance of photovoltaic devices. In 2012, a newly high *T*_c_ multiferroics of KBiFe_2_O_5_ with narrow band gap has been reported by Zhang *et al.* [397], which possesses high photovoltage (8.8 V) and photocurrent density (15 mA/cm^2^) comparable to the reported BiFeO_3_, opening a new avenue to discovering and designing optimal FE compounds for solar energy applications. Later in 2013, Zhang *et al.* [396] reported a much enhanced photovoltaic effect in metal/PLZT/ITO cells by using low work function metals as the electrodes suggesting that electrons in the metal with low work function could be photo-emitted into PLZT and form the dominant photocurrent in the devices. Despite the results that short-circuit current density and open-circuit voltage of Mg/PLZT/ITO cell was about 150 and 2 times larger than those of Pt/PLZT/ITO cell under AM1.5 illumination (see Figure 15c), they also demonstrated that the photovoltaic response of PLZT capacitor was expanded from ultraviolet to visible spectra, and revealed a promising way for further improved photoelectric performance via optimizing the thickness and crystalline quality of ferroelectric materials as well as using an anti-reflection layer to reduce light losses.

Although the possibility to output high photovoltage and photocurrent has made ferroelectric thin films competitive to traditional semiconductor devices reconsidered for targeted applications, for pursuit excellent photoelectric and photodiode effects in ferroelectric thin films, the intrinsic properties regarding electronic transport and the origin of their internal field should be more intensively studied. Also, new efficient ferroelectric-related photovoltaic materials with a band gap matching the solar spectrum, a large light absorption coefficient, as well as an intermediate carrier concentration should be exploited. 

#### 4.4.3. Effect of Mechanical Load: Giant Piezoelectric Resistance Effect

**Figure 16:**
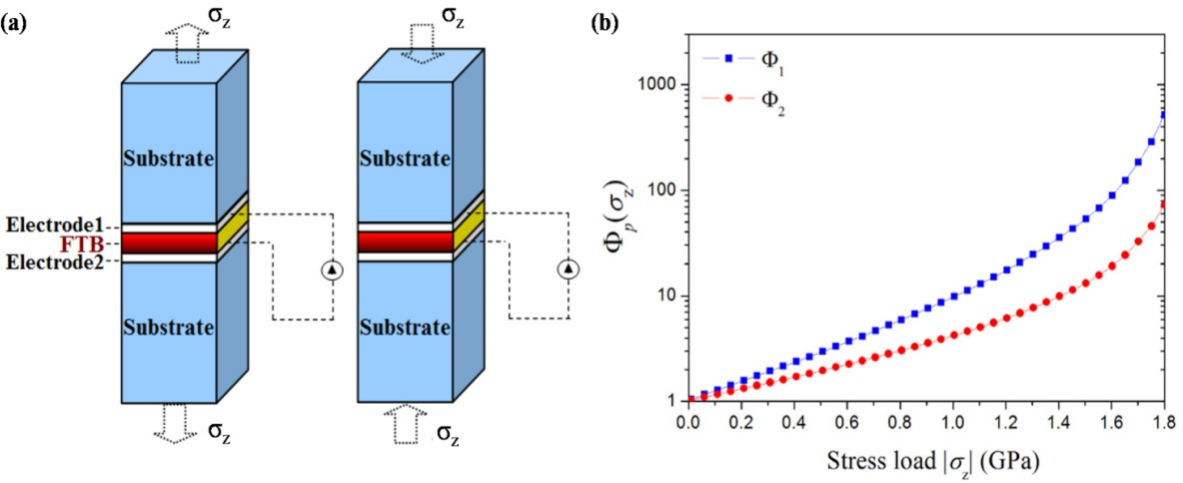
(**a**) Schematic structures of FTJ with applied tensile and compressive stress loads; (**b**) Stress dependence of the giant piezoelectric resistance (GPR) ratio Φ *_p_* (σ_z_). (From the work of Zheng *et al.* [411]).

As what has been introduced in our former text and based on theoretical researches, tunneling conductance across a FTJ can vary more than an order of magnitude depending on the direction of the polarization relative to the electronic transport [381]. According to previous theoretical studies, the polarization is a strong function of the applied mechanical load in many nanoscale ferroelectrics [408,409,410,411,412]. Thus the applied mechanical stress is highly likely to have influence on the electronic transport behavior and further the resistance of a ferroelectric thin film, resulting in giant piezoelectric resistance (GPR) effect. Similar to the GER effect due to polarization reversal, it is found that reversing an applied stress can also change the tunnel barrier sufficiently to produce a similar effect, which is particularly significant near the stress-dependent para/ferroelectric phase transition. Results of their works indicated that the conductance of FTJs can be modulated not only via the polarization orientations, but also by the applied stress giving rise to a GER-like effect, which they call GPR effect (shown in Figure 16 [411]). It can be seen that similar to reversing the polarization, reversing a modest applied stress may also change the height of the tunnel barrier sufficiently large to produce orders of magnitude change in the electro-resistance. Indeed, the sensitivity of the GPR system is adequate for high-sensitivity electronic and mechanical sensors, memories or other nano-devices. Although a variety of theoretical results have been obtained, experimental researches on this “new” phenomenon are relatively lacking. 

In the present experiment, mechanical load which causes piezoelectric resistance effect in a ferroelectric thin film is commonly arising from substrates or dissimilar electrodes. Considering that PZT thin films can possess a strong piezoelectric response and on the basis of the fact that many transition-metal oxides usually show a strong sensitivity of electronic properties to distortions of the crystal lattice, Tabata *et al.* [413] have demonstrated that respectively heteroepitaxial growth of bilayer films of PZT and manganites can open a perspective towards strain control of electronic properties. In the investigation of nonvolatile electronic writing of epitaxial Pb(Zr_0.52_Ti_0.48_)O_3_/SrRuO_3_ heterostructures established by Ahn *et al.* [144], metallic SrRuO_3_ layer is additionally found to be modified with the sheet resistance being changed by up to 300 ohms per square, suggesting piezoelectric resistance effect. Some later investigations have shown reproducible resistance hysteresis in few nanometer wide manganite channels controlled by the electrical polarization in an adjacent PZT layer [414,415], while challenges come in the hindering deriving from substrate clamping effect on of epitaxial film structures, which have been to some extent breakthrough by Dale *et al.* [416] via use of BTO substrates. They observe 30% changes in resistivity of La_0.5_Sr_0.5_MnO_3_ thin films for strain induced by the structural phase transitions of the BTO substrate, with a 12% change of film resistance induced by an inverse piezoelectric effect at room temperature. Meanwhile, the resistance changes induced by lattice strain and electric field effects have not been distinguished until the year 2005, when Thiele *et al.* [417] demonstrated the butterfly like hysteretic resistance modulations in epitaxial ferroelectric-ferromagnetic field effect devices of PZT/LSMO (with narrow manganite channels less than 7 nm) commensurate with strain variation from inverse piezoelectric effect of PZT. Contributions from electric field effect and strain effect have been distinguished for devices with varied channel thickness according to the type of observed resistance hysteresis loops, revealing the dominant position of piezoelectric effect over field effect.

Later on and recently, more studies have been focused on some other piezoelectric substrates for resistance tuning through piezoelectric resistance effect. For instance, the effects of substrate-induced lattice strain on the resistance of the LSMO films have also been investigated with PMN-PTO single-crystal substrates by Zheng *et al.* [418]. They achieve the modulation in the resistance of LSMO film through an applied electric field, which can *in situ* induce an in-plane compressive (tensile) strain in PMN-PTO substrate via the converse piezoelectric effect, and will transfer the strain to LSMO film which can also impose an in-plane compressive (tensile) strain on LSMO film, thereby causing a linear decrease (increase) in the resistance. Similarly, Guo *et al.* [419] have reported that the resistance of La_0.8_Ca_0.2_MnO_3_ (LCMO) thin films strongly depends on the electric field applied on the LCMO/PMN-PTO structure, indicating the effects of substrate-induced strain on the transport properties of thin films. Moreover, some piezoelectric resistance effect related phenomena and coupling effect also draw much interest, such as the work by Wang *et al.* [420], which focuses on electrical resistance load effect on magnetoelectric coupling of magnetostrictive/piezoelectric (Terfenol-D/PMN–PTO) laminated composite, providing the basis for the design of magnetoelectric sensors and their signal-processing as well as electronic circuits.

#### 4.4.4. Domain Wall Conduction

**Figure 17:**
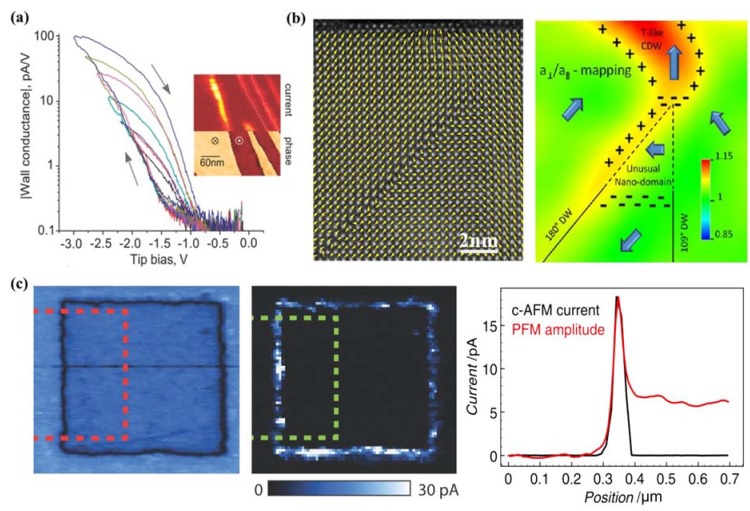
(**a**) A series of hysteretic I-V curves obtained sequentially on the surface of BFO film by increasing peak negative bias after each consecutive I-V curve, with the arrows showing the direction of the forward (toward increasing negative) and reverse tip bias sweeps. The inset shows microscopic images of piezoresponse phase (bottom half) and current (top half) acquired simultaneously on the surface of a film with dashed lines marking the center of the 109° domain walls as judged from the phase image [423]; (**b**) Z-contrast STEM image of the triangular 109°/180° domain wall junction associated with a conductive domain wall (CDW) near the free surface, with corresponding spatial distribution of the -D FB vectors (left) and color maps of the a_⊥_/a_||_ ratio (right), which schematically indicating the polarization orientation and bound charge, suggesting formation of a T-like CDW and an unusual nanodomain [424]; (**c**) domain wall conduction in PZT ultra-thin film: PFM amplitude images of a square domain written on a with a positive tip bias (left), c-AFM measurement at −1.5 V tip bias, showing an average current value of 25 pA at the position of domain walls (middle), and line profiles of c-AFM current and inverse PFM amplitude averaged over the left domain wall, as indicated by the dashed lines (right) [425].

Recently, abundant attentions have been attracted on the experimental investigations which show interesting electrical transport properties at ferroelectric domain walls, especially in multiferroic materials like BFO. Multiferroic insulator-like BFO thin films are currently closely focused on and widely studied, which have been demonstrated to possess ferroelectric domains with significant conduction in their domain walls, including 109°, 180°, and 71° domain walls [421,422]. Obvious evidence can be found through AFM techniques (see Figure 17a [423]). The atomic scale structure and properties of conductive domain walls (CDWs) also have been investigated, revealing that the CDW in rhombohedral-like BFO thin films not only induces the formation of a tetragonal-like crystal structure at the CDW but also stabilizes unexpected nanosized domains with new polarization states and unconventional domain walls (see Figure 17b) [424]. Besides the popular material of BFO, variable levels of local electrical conductance have been measured at ferroelectric domain walls in some other materials such as insulating Pb(Zr_0.2_Ti_0.8_)O_3_ thin films (see Figure 17c) [425], YMnO_3_ single crystals [426], and hexagonal HoMnO_3_ [427], *etc.* To clarify the mechanism of domain wall conduction, large amount of studies both experimental and theoretical have been carried out. At BiFeO_3_ domain walls, oxygen vacancy defect states inside the band gap have been observed clearly induce and modulate the dynamic conduction [423,428], which also applied to domain walls in YMnO_3_ single crystals [426]. For domains in reported PZT thin films, however, band gap narrowing should not play a significant role, while defect segregation at partially charged domain walls which is potentially controlled by strain and electrostatic boundary conditions, is considered to in relevance with the conduction [425]. The significant studies by Wu *et al.* [427] suggest that this enhanced conduction originates from holelike charge carriers that have accumulated to screen the negative bound charges, which demonstrates that topological defects can be harnessed to stabilize charged 180° domain walls in multiferroics, opening up opportunities for a new kind of nanoscale conduction channel in multifunctional devices. 

Although no clear consensus has emerged on the microscopic origin of the conduction, it has been known that such materials with conductive domain walls often have unconventional mechanisms driving the formation of domains, in particular in multiferroics in view of their magnetic properties. Interfaces in thin films, which can locally break the symmetry induce stress, and vary the bonding between ions, are also believed to have effect on domain wall conduction, with converse impact of giving rise to changes in bandwidth, orbital interactions and level degeneracy, opening venues for modifying the electronic structure of these strongly correlated materials [428]. It has been suggested by Seidel *et al.* [429] that artificially engineered oxide interfaces may pave the way to novel tailored states of matter with a wide range of electronic properties, including domain walls. Structure, properties, and dynamics of topological defects order parameter fields also indicated to offer a fascinating window in the physics of domain wall conduction [430]. Topological defects will possibly define macroscopic functionality either through macroscopically averaged responses over multiple non-interacting defects, or emergent phenomena due to defect-defect interactions. Depending on the advanced high-resolution electron microscopy and SPM techniques, systematic studies have been experimentally established on the internal structure, controlled design and even creation of topological defects in ferroelectrics; however, its internal structure and local properties have long remained elusive [430].

Overall, charged domain walls are of significantly scientific and technological importance as they have been shown to play a critical role in controlling the switching mechanism as well as electric, photoelectric, and piezoelectric properties of ferroelectric materials [424]. Manipulation of electronic states in complex oxides through designing and controlling of the electronic structure and chemical pressure at domain walls is important and promising for the next generation electronics applications. Meanwhile, experimental conclusion of dynamic conduction mechanism in domain walls is yet little, much more researches should be done on domain walls in those studied materials, as well as for more new materials that can exhibit similar effects, and even further to “engineer” the topological structure of the domain wall to controllably induce electronic phase transitions. Studies searching for domain wall conduction in a large range of ferroelectric materials should be envisaged to identify the best candidates for potential devices, in particular to investigate the tuning effects of intrinsic domain wall structure on the conduction.

#### 4.4.5. Resistive Switching

As mentioned above, ferroresistive switching behavior in ferroelectric films, which is based on the intrinsic switching of ferroelectric domains without invoking charged defect migration, has been investigated for decades. Since first discovered experimentally by Blom *et al.* [431] in PbTiO_3_ perovskite thin films, non-destructive readout of the binary information is believed possible from the bipolar switching between high- and low-conductance of a ferroelectric diode under two opposite polarizations, which have been further verified in other ferroelectric thin films such as PZT and BFO [245,432,433], *etc*. Recent experimental studies by Jiang *et al.* [246] and Pantel *et al.* [390] have also clearly demonstrated that ferroelectricity and conductivity coexist in a single phase with the conductivity can be modulated by ferroelectricity, realizing nondestructive resistive readout. For capacitors with ultrathin films sandwiched by electrodes, however, the probably existing defect-mediated leakage paths will cause an overwhelming leakage current, which may swamp the tunneling current and make the switching signal unreadable, leading to application limitations. 

**Figure 18:**
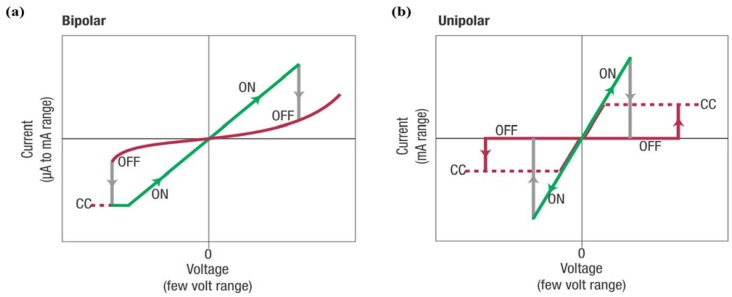
Classification of the switching characteristics in a voltage sweeping experiment (**a**) bipolar switching; (**b**) unipolar switching (cited from the paper of WaseR *et al.* [437]).

Comfortingly, another resistive switching (RS) phenomenon based on the electrically stimulation on change of the resistance, which usually related to defect-mediated effect, have been found and sprung up in ferroelectric thin films, attracting much attention for the particularly promising ability for perovskites to provide bistable switching of the conductance between non-metallic and metallic behavior by the application of an appropriate electric field. Commonly, a metal/insulator/metal (MIM) capacitor is engaged in RS investigations, where the insulator material typically shows some ion conductivity. Reproducible RS process can be achieved via proper applied electrical signals usually with compliance current to prevent device breakdown, which have been reported in various film-capacitors including transition metal oxides, several chalcogenides, and particularly popular perovskites in recent [434,435,436]. Both bipolar and unipolar switching processes have been observed, on account of whether the switching procedure depends on the polarity of the voltage and current signal, which have been schematically shown in Figure 18a,b [437]. In comparison with the largely investigated metal oxides, ferroelectric thin film-based RS behavior is engaged in relatively less studies with intensive studies can be found through the beginning of new century. Large amounts of attempts have done to RS behavior mainly focusing on the aspects of developing new types of highly efficient materials as well as to reveal the mechanism of RS behavior. In 2006, Szot *et al.* [438] demonstrated that the switching behavior was an intrinsic feature of naturally occurring dislocations in STO single crystals, which was shown to originate from local modulations of the oxygen content and to be related to self-doping capability, predicting that thin films seemed particularly promising for high packaging densities of memory elements. In the same year, two-level and multilevel RS behavior had been reported reproducible and stable in capacitor-like epitaxial BST thin films by Oligschlaeger *et al.* [439], manifesting the existence of intermediate states and indicating two or more resistive state may exist in a RS process. In many cases, two independent switching phenomena which respectively attributed to the ferroelectric switching process and resistive switching can co-exist in a capacitor-like ferroelectric film device. To distinguish the two phenomena, Kohlstedt *et al.* [440] had carried out experiments on SrRuO_3_/PbZr_0.2_Ti_0.8_O_3_/Pt ferroelectric capacitors using a c-AFM, from which the out-of-plane piezoelectric response and the capacitive and resistive current were simultaneously measured as a function of applied bias voltage.

**Figure 19:**
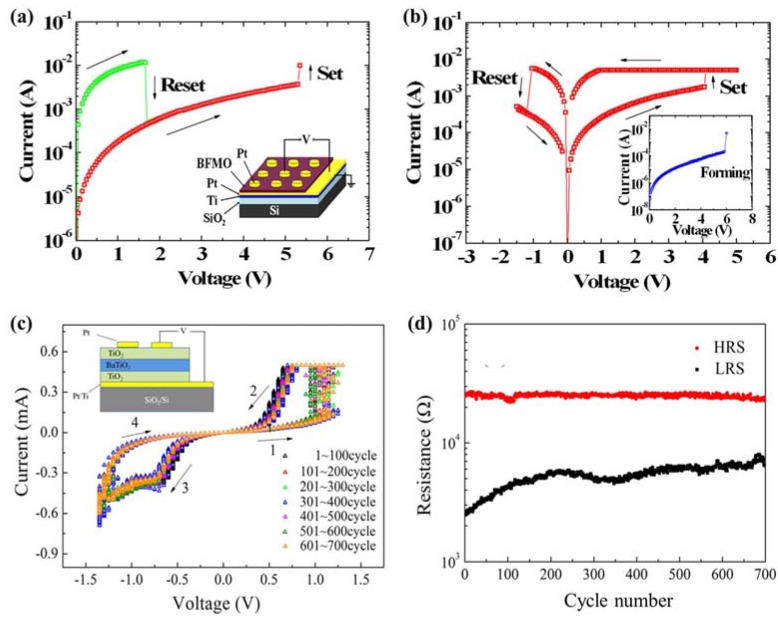
The typical current-voltage behaviors in Pt/BFMO/Pt devices: (**a**) unipolar resistive switching; and (**b**) bipolar resistive switching with absolute values plotted in semi-log scale (Luo *et al.* [443]). The bipolar resistive switching characteristics in TiO_2_/BaTiO_3_/TiO_2_ device: (**c**) I-V curves with 700 consecutive cycles; and (**d**) distributions of V_set_ and V_reset_ in 700 cycles (Ma *et al.* [444]).

With respect to mechanism of RS behavior, although several conduction models have been proposed to explain the origins of RS effect, which contains conductive filament model [247,437], Schottky barrier model [246,441] and charge trap-detrap model [434], none agreement have been reached so far. In spite of having efforts for mechanism investigation, currently, many researchers have devoted themselves to develop methods for improving the reliability and resistance ratio of RS devices. Doping has been regarded as an effective one, in particular for multiferroics which present a tantalizing evolution of doping-driven phase competition between energetically similar ground states. Yang *et al.* [442] have presented the observation of an electronic conductor–insulator transition by control of band-filling in Ca-doped BFO thin films, with the applied electric field along both the normal and in-plane directions. The effect of divalent-ion-calcium doping has been considered and the mechanism of electronic conduction modulation has been demonstrated based on the interplay of ionic and electronic conduction. More works have been established for optimization of RS behavior in ferroelectric devices through ion doping. For instance, in 2012, robust nonpolar RS behaviors have been reported in Pt/BFMO/Pt memory devices by Luo *et al.* [443] (see Figure 19a,b), with intensive RS mechanism being studied. More recently, a new concept for RS device optimizing by alternative structures of oxide/ferroelectrics/oxide has also been put forward by Ma *et al.* [444], where attractively highly uniform bipolar resistive switching behaviors have been demonstrated in TiO_2_/BaTiO_3_/TiO_2_ multilayer, verifying well the practicability and reliability of this structure (see Figure 19c,d). 

### 4.5. Ferroelectric Fatigue

As what has been observed in many experiments, ferroelectrics may exhibit fatigue process along with time, temperature variable, as well as the applied electric field. For ferroelectric thin films, the fatigue can be mainly divided into three kinds, including polarization fatigue, electrical breakdown, and retention failure. Electrical breakdown is supposed to arise from dendritelike conduction pathways through the material, initiated at the anodes and/or cathodes. Electrical breakdown in ferroelectric oxides is a hybrid mechanism which is generally influenced by film thickness, ramp rate, temperature, doping, and electrodes. Retention failure comes since retention time for device is linked to fatigue process in ferroelectric thin films, *i.e.*, when a potential builds up over a period of time, it can destabilize the ferroelectric polarization state and thus cause loss of information [195]. Previous studies have suggested that retention failure is a result of oxygen vacancies migration and redistribution under the depolarization field or other built-in fields in the material [445,446]. In the following we will mainly introduce polarization fatigue, which has received most of concern.

Most ferroelectrics exhibit degradation in switched polarization during the applied electrical cycling, *i.e.*, polarization fatigue. It is one of the most serious device failure mechanisms in ferroelectric thin films, especially in devices with Pt electrodes. The mechanisms of polarization fatigue have been investigated for decades with many physics models being put forward for explaining the phenomenon. Electrical stress can probably either develop a passive dielectric layer in a film capacitor, giving rise to a decrease of the effective electric field [447], or generate pining force on domain wall motion, leading to domain pinned in a particular direction or domain nucleation inhibited [448,449]. In respect of the latter effect, direct experimental results have shown its origin attributing to oxygen vacancy migration [450,451] or electron/hole injection from electrodes [452] near interfacial region. Despite those works utilizing AFM and Raman spectroscopy techniques, X-ray microdiffraction should be a significant technique for precisely observation about how regions of the film become fatigued, through which reversible fatigue effect under relatively low field and irreversible fatigue effect under very high field, have been observed [453]. Although the commonly used Pt electrode has high work functions, adsorption of oxygen onto Pt surfaces will cause obvious polarization fatigue [240]. To decrease polarization fatigue, large amount of attempt have been established on electrodes. Some oxide electrodes, such as iridium oxide, ruthenium oxides have been demonstrated can decrease the fatigue in PZT thin films [179,454], which have been explained by de Araujo *et al.* [455] for the fact that oxide electrodes can reduce or deoxidize reversibly and repeatedly without degradation. Besides, dopants for PZT films with Pt electrodes have also been demonstrated to be efficient to improve film fatigue performance [456]. 

These years polarization fatigue in PZT thin films are still attracting much attention. Polarization fatigue has been demonstrated to in relevance with polarity of electrical cycling in lead zirconate (PZO) and PZT thin-film capacitor by Lou *et al.* [457,458]. Their experiment shows that unipolar fatigue is noteworthy but less severe than bipolar fatigue, which is attributed to the switching-induced charge injection or a series of periodic events of polarization backswitching and switching during unipolar cycling. They for the first time estimated the effective thickness of cycling-induced phase-decomposed pyrochlorelike layer during bipolar/unipolar fatigue according to the dielectric results. More complicated fatigue studies with thermal influence have been carried out by Cao *et al.* [459], who observed irreversible deteriorations of ferroelectric properties with increasing fatigue–annealing cycles which probably due to the element diffusion at the metal/ferroelectric interfaces accelerated by the phase decomposition. Measurement frequency has also been verified to affect polarization fatigue [460].

**Figure 20:**
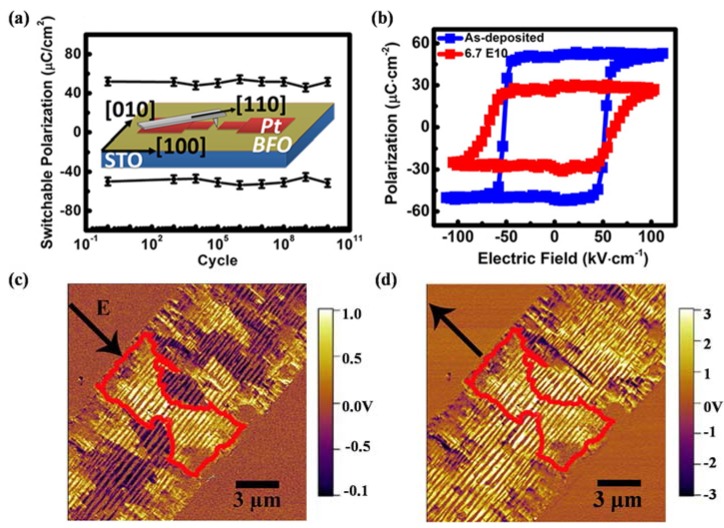
(**a**) Switchable polarization obtained from the remanent hysteresis loops of a planar (001)-oriented BiFeO_3_ device shows no fatigue up to 10^10^ cycles, with the inset showing the planar device configuration; (**b**) fatigue of BiFeO_3_: remanent hysteresis loops indicate reduction of switchable polarization after 6.7 × 10^10^ cycles; (**c**,**d**) IP-PFM images which demonstrate domain pinning across the channel (red outline). (Zou *et al.* [465]).

BFO thin films also have attracted much attention with respect to fatigue, leading to a number of works [461,462], where motion of oxygen vacancies and the injection of electrons, as well as low frequency and low field related charge trapping and domain wall pinning are considered important to fatigue process. Particularly, in the study of Ke *et al.* [463], an anomalous fatigue behavior is observed in the La and Mg co-substituted BiFeO_3_ (BLFM) thin film, where the polarization is enhanced rather than degraded upon the repeated switching cycles, owing to evolution of charged defects in the film during the fatigue process. The nature of polarization fatigue in BFO thin films have been intensively investigated by Baek *et al.* [464]. According to the results that intrinsic polarization fatigue depends on the different switching path in monodomain epitaxial BFO thin films, they have proposed a model that the complex multistep switching process of 180° polarization reversal results in domain wall pinning by incorporation of mobile charge carriers into non-neutral domain walls, providing design rules for the reliable performance of multifunctional devices controlled by polarization switching. Moreover, Zou *et al.* [465] have demonstrated direct observation of charge-polarization interaction and domain evolution during cycling in a planar BFO device, revealing that the electron-injection-pinned domains grow across the channel upon further cycling and eventually lead to fatigue, which is of great significance to better understand the mechanism of ferroelectric fatigue (see Figure 20). Additionally, polarization fatigue in ferroelectric polymers has drawn increasingly interest due to their potential use in nonvolatile memories [466].

Accompany with the studies on fatigue mechanism, much effort have been done to pursuit fatigue-free devices with ferroelectric thin films, which almost have been achieved by SBT-based capacitors [455,467], PZT or PLZT thin films with oxide electrodes [468,469], Bi_4_Ti_3_O_12_-based films capacitors [470,471], as well as buffer layer or ion doping modified BFO film capacitors [472,473], *etc.*, generally with fatigue-free behaviors up to 10^10^ switching cycles. Meanwhile, as the devices integration trend, more works are needed for fatigue mechanism and fatigue-free studies in ferroelectric film-related capacitors.

## 5. Applications

The fruitful phenomena and properties in ultrathin ferroelectric films provide wonderful possibilities in device applications. Currently, utilizing their dielectricity, ferroelectricity and piezoelectricity, ferroelectric thin-film structures integrated onto Si chips have made inroads into the semiconductor industry. They are playing more and more essential roles in microelectronics and microelectromechanical systems (MEMS). Moreover, ferroelectric thin films have found their important applications in memory devices, integrated optics and optoelectronics field, microwave devices and even more broaden fields. With respect to ultrathin ferroelectric films, many applications of thicker films still work well, according to the size downscaling law of the properties. Besides, applications utilizing the unique properties of ultrathin ferroelectric films, such as quantum transport properties, abnormal electromechanical behaviors, and giant flexoelectricity, *etc.*, are promising. In this section, we conduct a survey of the main applications of ferroelectric thin films, with special attention to those of ultrathin films. The general device concepts and structures, overview of current application and future prospects, as well as some challenges are to be included.

### 5.1. Applications in Memory Devices

#### 5.1.1. Ferroelectric Random Access Memory (FRAM)

One of the most important applications for ferroelectric thin films should lie in nonvolatile memory, which is more reliable and effective in practical use than that of volatile memory such as dynamic random-access memory (DRAM). For a typical ferroelectric random access memory (FRAM) device, ferroelectric thin film is usually sandwiched by electrodes, with the “0” and “1” information represented by the stable negative or positive remanent polarization state [191,125]. Readout operation is usually performed by altering the polarization state with an applied voltage, manifesting with an output current dependent on the stored polarization state. Such a readout operation is destructive as the stored polarization may be erased by the large applied voltage [474]. FRAMs show attractive features for electronic application such as fast access speeds (5 ns), high densities (64 Mb), and low power consumption, *etc.*, which is to some extent superior to the performance of other nonvolatile technologies. Nevertheless, due to the destructive readout, the challenges and limits are met in retention time and endurance performance of FRAMs. To overcome the key issue of reliability, attempts have been done to exploit enduring design of FRAM cells and newly fatigue-free structures for the targets of increasing the writing speed, endurance and decreasing the read/write voltage [475,476].

**Figure 21:**
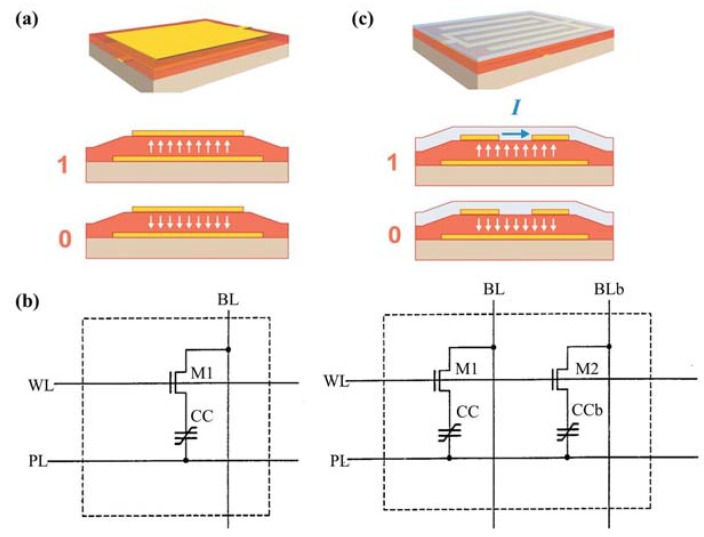
(**a**) Schematic image of a typical ferroelectric random access memory (FRAM) cell (from Heremans *et al.* [477]); (**b**) circuits of a one transistor–one capacitor (1T–1C) design (left), and a two transistor–two capacitor (2T–2C) design (right) [478]; (**c**) illustration of the structure for a FeFET (from Heremans *et al.* [477]).

**Figure 22:**
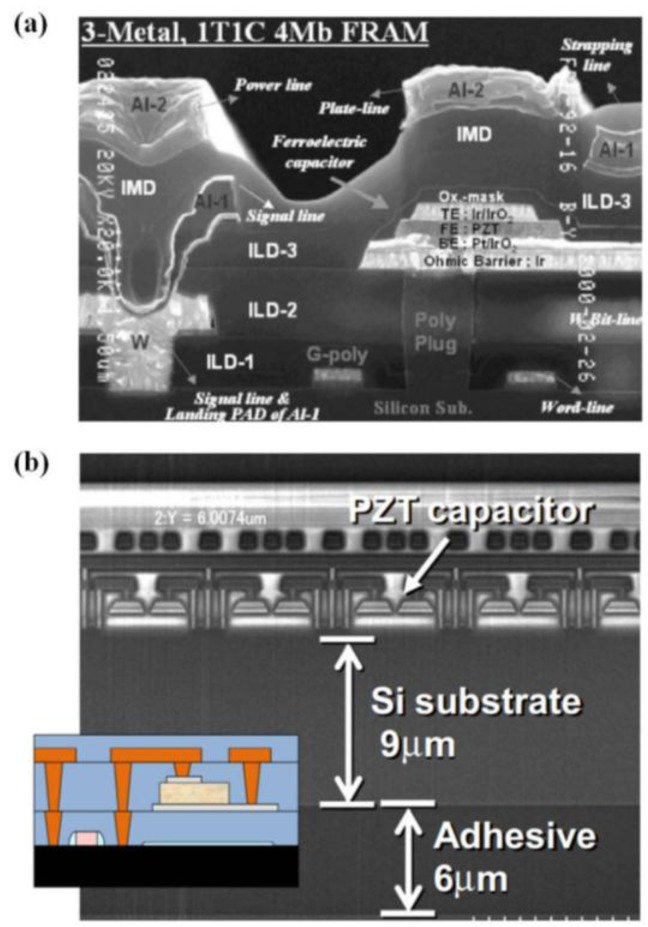
(**a**) Cross-sectional SEM image of a real commercially used FRAM: the Samsung 4 Mbit1T-1C 3-metal FRAM [195]; (**b**) cross-sectional SEM image of FRAM memory containing PZT capacitors (taken by Kim *et al.* [481]).

Schematic image of a typical FRAM cell is illustrated in Figure 21a [477], where a pass-gate transistor is required to control the applied voltage thereby guarantee the selection of a certain cell from an array of the FRAM cell. Most widely used electrical circuit for memories are circuits of a one transistor–one capacitor (1T–1C) design or a two transistor–two capacitor (2T–2C) design as Figure 21b shows [478], in which 1T–1C design is much more space effective but the reference capacitor will fatigue much faster, contrarily, 2T–2C design possesses better device life but with high space occupation. To improve performance and reliability of FRAMs, many circuits for high performance device application have been designed based on the 1T–1C and 2T–2C designs, with many patents being reported [479,1]. One typical commercial use is the Samsung 4 Mbit 1T-1C 3-metal FRAM (see Figure 22a [195]). To decrease the size of the memory cell and provide a nondestructive readout, ferroelectric field-effect transistor (FeFETs) is a good choice. It is practically a metal-oxide-semiconductor FET (MOSFET) whose gate dielectric is ferroelectric (conceptually shown in Figure 21c [477]). Meanwhile, mainly due to the current short retention time, together with some harmful interface traps and reactions at ferroelectric-semiconductor interfaces, FeFETs have not been commercially used. They are of great interests but needs more tremendous researches on device designing with various material combinations and different layouts.

It has been for some year using PZT for integrated ferroelectric memories. Also efforts have been done to use new materials or capacitor structures to improve performance of FRAM devices, for lead-free trend and difficulty in achieving >10^12^ read/write cycles. Investigations for fatigue-free ferroelectric film capacitors have brought many new ferroelectric capacitors based on BST, Bi_4_Ti_3_O_12_, PZT or PLZT thin films with oxide electrodes, and capacitors with Pb_5_Ge_3_O_11_ thin films [482], *etc*. Particularly, BST has marketable FRAM products, attributed to its high switching speed of 0.5 µs and endurance of as high as 10^11^ read/write cycles [482]. However, devices with BST thin films have fundamental limitations in retention time (~10^5^ s at 85 °C [483]), as well as in relatively low memory capacity. Bi_3.25_La_0.75_Ti_3_O_12_-based film capacitors have also been employed for FeFETs, which, however, have poor performance with lower switching speed (8.6 μs) and more limited retention time (~10^4^ s at 27 °C) in comparison with SBT [484]. 

Recently, PZT thin films have been reported to achieve up to 10^14^ endurance cycles, same-state and opposite-state retentions up to 10^3^ h, and stable operation in the range of −40 °C to 125 °C [485], which can bring more promising commercial FRAM products. Additionally, HfO_2_-based ferroelectrics such as Si-doped HfO_2_ ultrathin films (10 nm), which can be deposited into high-aspect-ratio geometries with exceptional scalability and CMOS compatibility, should have great potential to enable the development of next-generation 3D FRAM capacitors [486,487]. Low access energy nonvolatile memory is also a target in FRAM devices, with challenges being encountered in sensing data at low power supply voltage. Some progresses have been achieved in devices designing. For instance, a successful design of 1 Mb 1T1C FRAM have been reported [488]. Polymer ferroelectric memories also have proved viable alternatives [489] in developing cheap and viable nonvolatile memories. More information about polymer nonvolatile devices can be referred to a recent review of Heremans *et al.* [477]. Furthermore, impact of wafer thinning process on FRAM has been evaluated by Kim *et al.* [481], with PZT capacitors being integrated onto thinning wafers (Figure 22b), implying that logic device based on strained silicon and low-k interconnect as well as FRAM memory can be applied to a 3D integration. 

#### 5.1.2. Resistive Random Access Memory (RRAM)

**Figure 23:**
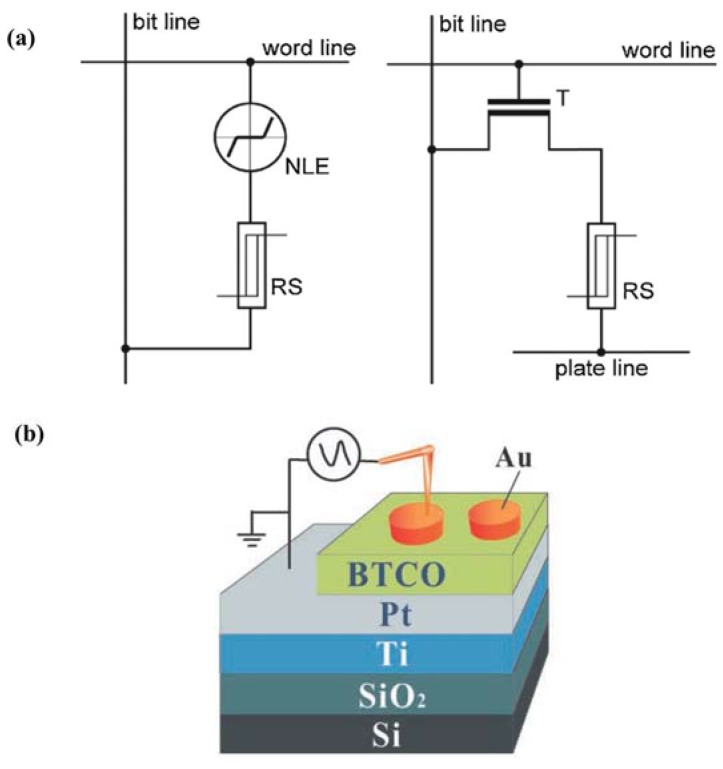
(**a**) Circuit diagram of a storage node in the matrix of a resistive random access memory (RRAM): passive matrix (left), in which NLE is a serial element with a specific non-linearity, and active matrix (right) with a select transistor T (from [436]); (**b**) schematic diagram of a high performance RRAM cell with the Co-doped BaTiO_3_ thin films as active layers (from [492]).

It should be pointed out that, presently adaptively room-temperature functional FRAMs of high performance and smaller size which can adapt to the fast developing electronic fields are still in desperate need. FRAMs exhibit technological and inherent problems in the scalability, which are hard to adapt to the continuously increasing density of Si-based Flash memory devices. To overcome the scaling problems, various new nonvolatile memory concepts have been put forward, among which resistive random access memory (RRAM) attracts a great deal of attention. RRAM is based on resistive switching behavior of metal-insulator-metal (MIM) structures and has been regarded as a notable alternative for FRAM. The OFF/ON states in a RRAM (corresponding to high-resistance/low-resistance states) can be reproducibly written (set) or erased (reset) by applying appropriate voltage. The state of the RRAM cell is detected by measuring the current under a small read voltage (V_read_). Two kinds of circuits of a storage node in the matrix of a RRAM have been shown in Figure 23a [436]. RRAM cells have been predicted as the fourth basic circuit element because of the conceptional symmetry with the resistor, inductor, and capacitor which can be described as memristors [490,491]. However, as what have been introduced in Section 4.4, although the resistive switching behavior has been studied actively over the past several decades, the precise switching and conduction mechanisms are still unclear with more woks being needed.

On account of commercial use, performance evaluation for a RRAM cell should consider the following criteria, *i.e.*, low write voltage (better in the range of a few hundred mV), short write time (desired to be <100 ns), low read voltage and current (less than approximately 1 µA) with short read time (preferably shorter than write time), relatively high R_OFF_/R_ON_ ratios (>10, for small and highly efficient sense amplifiers), good endurance (preferably more than the range of 10^3^ to 10^7^), as well as long retention time [436]. For this purpose, a variety of studies have been established on RRAM capacitors to exploit high performance cells. Despite the area scaling investigation of the basic RRAM cells of MIM structures, c-AFM is usually used to elucidate the nanoscale current distribution and switching properties of thin film heterostructures. While compared to the widely reported transition metal oxides which possess excellent RS behavior, ferroelectric thin films RRAM cells with satisfactory behaviors are less reported. One exciting example for high performance RRAM cells is achieved via using the Co-doped BTO thin films as active layers by Yan *et al.* [492], with the cell structure schematically shown in Figure 23b. The stable unipolar resistive switching characteristics, with a R_OFF_/R_ON_ ratio over 10^4^, retention time longer than 7 × 10^4^ s, endurance over 10^5^ cycles, and switching speed less than 10 ns/70 ns for the set/reset, have been demonstrated, which is of great potential for commercial use. Therefore, it has reasons to believe that ferroelectric-film-capacitor based RRAMs are promising nonvolatile memory of the future.

### 5.2. Piezoelectric and Integrated MEMS

MEMS often refers to a functional miniature system combining electronics with other components such as sensors, transducers, and actuators through integrating and micromachining techniques, which has drawn much attention since its first presence as a technological discipline during the 1980s. Practically used MEMS processing have profited from the advances in film deposition as well as a general improvement of micromachining techniques such as dry etching techniques. Along with the trend of miniaturization and integration trend, functional ferroelectric thin films of high energy density in particular with excellent piezoelectric properties, have been favorably incorporated in MEMS offering integrated actuation, sensing, and transduction. On the basis of piezoelectric and electrostrictive effect, ferroelectric thin film devices in MEMS can provide a direct transduction mechanism to not only convert signals from mechanical to electrical, which can be configured as sensors, but also convert electrical signals to generate strain or stress which can configured as actuators. Reviews on piezoelectric MEMS and on issues of ferroelectric thin films for micro-sensors and actuators are available [493,494,495,496]. 

Commonly, metrics such as the piezoelectric coefficients, electromechanical coupling coefficient, mechanical quality factor and acoustic impedance, *etc.*, have been employed to evaluate the effectiveness of ferroelectric thin films for MEMS applications. PZT thin films have been considered as a suitable choice, which exhibit clear advantage in actuator applications with piezoelectric coefficients being 5–30 times higher than those of commercially used ZnO and AlN structures. Efforts have been undertaken over decades to integrate PZT (typically 0.5–2 µm thick films) into MEMS devices and to demonstrate applications. The effective transverse piezoelectric coefficient of sol–gel processed PZT thin films has been investigated as a function of composition, film texture and film thickness [116], which provides reference for device optimizing. The most frequently applied PZT thin films are those with optimal composition at the 53/47 or 52/48 proportion which exhibits outstanding transverse piezoelectric properties at the morphotropic phase boundary, among which {100}-textured PZT_53/47_ thin films are in favor for their good integrating performance on silicon devices. Although almost only PZT thin films are currently in use in MEMS, other ferroelectric thin films have also been reported as promising candidates for MEMS such as relaxor ferroelectrics [497], the recently reported lead-free thin films [498] and some piezoelectric polymers (which have been recently reviewed by Ramadan *et al.* [499]). A study carried out by Baek *et al.* [500] has demonstrated strong piezoelectric activity of PMN-PT epitaxial thin films, which dramatically enhance the freedom of designing small devices with better performance. Integrating methods for piezoelectric materials into MEMS also of great importance, including bulk micromachining, surface micromachining, and integrative approach (*i.e.*, integrate precision micromachined silicon structures onto PZT substrates), which have been introduced in detail somewhere else [496,501]. 

**Figure 24:**
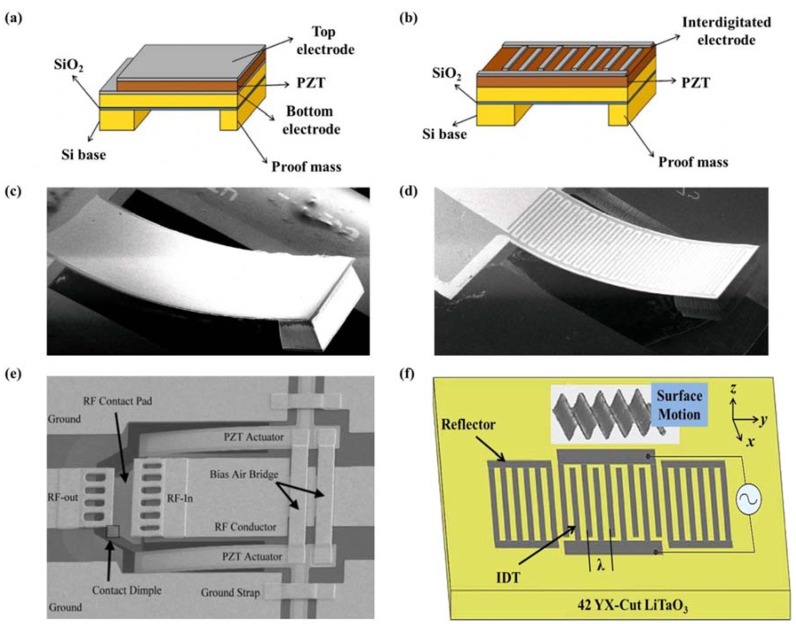
Schematic diagram of the piezoelectric microelectronics and microelectromechanical systems (MEMS) generators: (**a**) {3–1} mode configuration; and (**b**) {3–3} mode configuration (from Lee *et al.* [504]). SEM photograph of the finished piezoelectric MEMS generators: (**c**) {3–1} mode device; and (**d**) {3–3} mode device [504]; (**e**) a SEM image of a PZT RF MEMS switch showing the residual-stress-engineered PZT actuator to result in a net negative curvature under unactuated conditions and allowing for a positive deflection upon {3–1} mode actuation (from Polcawich [507]); (**f**) a surface acoustic waves (SAW) resonator structure (from Frank Z. Bi and Bradley P. Barber [510]).

With respect to MEMS devices, PZT micro cantilever devices are attractive. In spite of the past reported applications such as PZT coated Si cantilevers for sensing and actuation in AFM heads [502], cantilever MEMS arrays fabricated by LG Microelectronics [503], there are still other promising progresses. Piezoelectric MEMS generators, which can convert ambient vibrational energy to electrical energy and can power a wireless sensor node, have been reported can be realized via different configuration modes, among which two common modes have been schematically illustrated in Figure 24a,b, with SEM images of the finished piezoelectric MEMS generators shown in Figure 24c,d [504]. Efforts have been done to improve the performance of this kind of devices by increasing the total power output or decreasing the power consumption and working duty ratio of a wireless sensor node [505,506]. Thereby, the ambient vibrational energy can be efficiently converted to electrical energy via the MEMS piezoelectric energy harvesting device and would significantly power wireless MEMS sensor node. Besides residual stress, engineering has also been effectively used to create cantilever structures with negative curvatures by Polcawich [507], with the fabricated PZT RF MEMS switch being shown in Figure 24e. Another cantilever-based piezoelectric device is piezoelectric accelerometers, which can be used for accurate inertial measurements, specifically for vibration level sensing and for monitoring temporal variations in acceleration [508,509]. Just as cantilevers, membrane based devices have also evolved from single element as micromotors to membrane arrays. The silicon micro-machined ultrasonic applications have drawn much interest which is on the basis of the fact that piezoelectric thin films can be integrated into freestanding micromechanical structures to create high pixel density arrays of ultrasonic transmitters and receivers, which has been well reviewed by Tadigadapa *et al.* [496].

Meanwhile, for ferroelectric thin films application in MEMS, some limitations must be considered, for instance, the deposition and crystallization processes for ferroelectric thin films typically involve high temperatures in the range 200–800 °C and these materials often include elements that are incompatible with standard CMOS technology. In comparison with the main momentum in ferroelectric films for memory devices, ferroelectric thin film-based MEMS have not yet established their positions in MEMS. There is an increasingly indepth knowledge on ferroelectric thin films as well as fast developing technology on low-temperature ultrathin-film (less than 100 nm thick) deposition, integration and micromachining. They are also considered as potential candidates for future devices in MEMS and even the next-generation nano-electromechanical systems (NEMS).

### 5.3. Surface Acoustic Wave (SAW) Devices

Surface acoustic waves (SAWs) are usually used for filters in mobile communication and passive signal processing devices, such as resonators [511], which also have limitation in miniaturization since they are difficult to fabricate on-chip. Conventional SAW devices employ periodic metallic interdigital transducers (IDTs) as electric input and output port, which is usually deposited on uniformly polarized piezoelectric crystals or films, transmitting applied radio frequency (RF) signals to the surface of piezoelectric, which will produce deformation thereby results in the launching and traveling of SAW along the piezoelectric surface. In contrast, the launched SAWs can subsequently be converted back to an electric signal by a receiving IDT. The structure of a SAW resonator has been shown in Figure 24f [510]. Along with the low-cost miniaturization trend as well as the need in increasing operation frequency of modern communication systems, acoustoelectric bulk acoustic wave (BAW) devices with improved insertion loss, temperature stability, power handling capability, electrostatic discharge protection, selectivity and mechanical quality factor, are very likely to take the place of discrete SAW components, particularly represented by the breakthrough in miniaturization of frequency-selective devices brought from thin-film bulk acoustic resonator (which is bulk micromachined, offering excellent temperature stability), solidly mounted resonator and high overtone bulk acoustic resonator technology. Many reviews are available on technology and devices of SAW and BAW, including design process, modeling, fabrication, as well as marketing application [512,513,514,515], *etc*.

Piezoelectric film materials for acoustic wave applications have changed markedly over the past twenty years in terms of available material types and the quality of individual samples. Piezoelectric AlN and ZnO thin films have been used in practice for BAW [516], with commercial products such as single resonators, bandpass filters and duplexers being launched by Agilent [517]. Although ferroelectric thin films have not been practically used, they present more excellent piezoelectric effects promising in application of high performance BAW devices than those of non-ferroelectric materials. As the functional and commercial needs, efforts on material exploiting, device design, modeling, and fabrication study have caused a rapid development of thin film resonator technology during the past years, with some ferroelectric thin films being reported as potential candidates for BAW, such as BST thin films [518,519], PZT thin films [520] as well as some thin polymer films [521], *etc*. 

In spite of material type, structure design and fabricating technology are also of great importance to BAW resonators and filters, with the reliability (for wireless applications, the critical issues involve mechanical stress environmental factors and operating life) of these devices must be taken into account, which have been introduced in detail in the reviews mentioned above. Efforts aiming at high-performance ferroelectric-thin-film based SAW and BAW devices are continuously going on. Specially, nanoscale ferroelectric domain manipulation and AFM writing of periodic piezoelectric transducers in epitaxial films are also promising approaches allowing the implementation of very high-frequency SAW devices, which has been verified by Sarin Kumar *et al.* [522] via successful fabrication prototypes of such devices on epitaxial PZT films. It is worth to note that proper substrates such as SrTiO_3_ and LaAlO_3_ are more preferable for ferroelectric film based components than the standard Si wafers, due to their ability to provide good lattice matching to the perovskite ferroelectrics. More recently, Vorobiev *et al.* [523] have studied the BAW performance in BiFeO_3_-0.33BaTiO_3_ (BF-BT) films, demonstrating that the variations in the BF-BT film microstructure can result in corresponding variations of the BAW resonator performance, which allows for optimization of the deposition conditions and achieving the high resonator performance. 

### 5.4. Application in Integrated Optics and Electrooptic Devices

Based on strong electrooptic effects and excellent nonlinear optical properties, ferroelectric oxides have long been used for signal processing in optical communication systems especially in optical modulators. The technology has been mostly satisfied by bulk LiNbO_3_ (LN), with 40-Gbps optical modulators having been commercialized. However, limitations in bulk single crystals, such as driving voltage *versus* device length, minimization limits, and dc drift, have stimulated the fast development of various kinds of ferroelectric thin films applying to integrated photonic circuits, high-speed optical devices, and electrooptic devices, such as electrooptic waveguide modulators and high-speed optical switches, *etc*. Increasing interests have lied in this field owing to the advantages of ferroelectric thin films, including large bulk electrooptic coefficients in epitaxial films, being almost transparent among large optic region (from the midinfrared to the visible spectral), high integratability, as well as strong optical confinement and high optical power density. In the following, we mainly focus on applications of ferroelectric thin films in electrooptic modulators.

#### 5.4.1. Electrooptic Waveguide Modulators

Ferroelectric thin film based waveguides devices have attract increasingly interests in recent years. Advanced film deposition methods such as PLD, MOCVD, MBE, RF sputtering and ALD have provide the opportunity for epitaxial growth of high-refractive ferroelectric thin films on low-refractive-index substrates with large linear electrooptic coefficients promising for high-bandwidth electrooptic wave modulators. An excellent review on ferroelectric epitaxial thin films for integrated optics application is available [524].

Enormous progress in material choice, film deposition and device processing for high performance waveguide devices with large electrooptic coefficient, has been shown for decades. A number of ferroelectric thin films deposited on various substrates (such as MgO, LaAlO_3_, MgAl_2_O_4_, and STO, *etc.*) with appreciable electrooptic coefficients, have been reported as potential candidates for electrooptic waveguiding devices. Besides those summarized by Wessels [524] including LiNbO_3_ (LN), BTO, PZT, PLZT, BST, KNbO_3_, (Sr,Ba)Nb_2_O_6_ (SBN:60, SBN:75) and electrooptic polymers, some other materials also have been demonstrated as electrooptical active materials with low propagation loss and large electrooptic effect, such as epitaxial PMN-PT thin films [525] and perovskite-structured Na_0.5_K_0.5_NbO_3_ thin films [526]. BTO thin film is very likely the material of choice, which exhibits an electrooptic effect much larger than that found in LN [527], with a large electrooptic coefficient on the order of *r* = 100 pm/V, and large birefringence due to its atomic structure, ∆*n* = 0.05 [528,529]. A cubic substrate of MgO with a low refractive index and microwave index is often chosen as the substrate for epitaxial BTO thin films, with which waveguides of different structures have been fabricated, with propagation losses and electrooptic performance being investigated [530]. Furthermore, doping seems to be an effective way for improving film qualities, with an example having been shown in the erbium-doped BTO film, which had the potential as an active optical gain medium in integrated devices [531]. In ferroelectric thin films, domains can be manipulated and switched by applying an appropriate electric field across the material, which is accompanied by a large change in the index of film due to the birefringence in the material, resulting in polarization depended optical properties. Specially, integrated heterostructures of a multiferroic BFO thin film onto STO-buffered MgO substrates have recently been reported to have a strong coupling between ferroelectric and optical properties, which make BFO a good candidate material for advanced electrooptic modulators operating over a broad range of temperature and frequency [245,532].

Even though numerous advantages have been demonstrated on thin film ferroelectric waveguides, optical losses are still the main limitation for their application, which can result from surface scattering (considered as the main loss mechanism which caused by the appreciable surface roughness in highly strained heteroepitaxial thin films, as well as the high optical field intensity at the surfaces and the interfaces) [533], scattering by complex grain and domain boundaries in a polycrystalline thin film (which is polarization dependent; therefore, a single-domain epitaxial thin film is desired for optimal performance, but difficulty to realize due to the existing complex influences of strains), and leakage loss, *etc*. Propagation losses are implied can be further reduced by growing films of the proper thickness and improving thickness uniformity [534]. In practical use, light loss should less than 1 dB/cm. For this purpose, some progresses have been made, with polarization-dependent loss as low as 0.1 dB/cm and propagation loss of less than 0.9 dB/cm being achieved by a novel Si_3_N_4_ strip-loaded BTO waveguide structure [535]. For ferroelectric modulators with ultrawide bandwidth, limitations may come in some aspects such as impedance mismatch, electrode losses, and phase velocity mismatch between microwave and lightwave, *etc*. Among them, phase velocity mismatch is considered to have primarily influence on bandwidth of electrooptic waveguide modulators, which maybe improved effectively by lower the microwave index such as using a composite structure consisting of SiO_2_/BTO/MgO layers [536]. In light of device speed, efficiency, and high-bandwidth, the ideal waveguide structure consists of an epitaxial thin film material with a high electrooptic coefficient deposited onto a material possessing a small microwave dielectric constant (low-index substrates) is preferred.

#### 5.4.2. Electrooptic Switch

**Figure 25:**
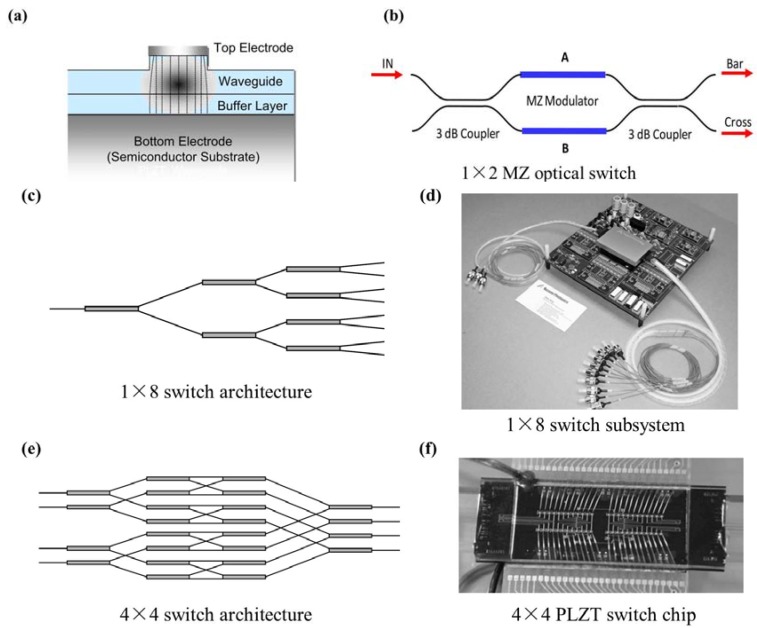
(**a**) Sandwiched electrode geometry for electrooptical switches; (**b**) the 1 × 2 MZ optical switch; (**c**) the designed 1 × 8 switch architecture; (**d**) a practical 1 × 8 switch subsystem; (**e**) the 4 × 4 switch architecture; (**f**) a practical 4 × 4 PLZT switch chip. (**a**,**c**–**f** are cited from [537], **b** is cited from [538]).

Another appealing application is the electrooptic switch, which realizes optical switching functions by using electrooptic effects, offering relatively faster switching speed. High-speed optical switches are attracting increasing interests for a variety of applications such as burst-switching and packet-switching for telecommunications and data communications including interconnection. The main difference between electrooptic modulators and switches is structure design of devices. The high confinement in thin film waveguides creates the potential for fast, low voltage electrooptic switching, facilitated by the small electrode separation which is allowed due to the small dimensions of these waveguides. PLZT has been reported as one of the best candidates in terms of high-speed, low-voltage, and low-power consumption optical switching due to its efficient voltage-induced index change. A sandwiched electrode/waveguide/buffer-layer/electrode structure makes it possible to optimize the overlap integral between an optical field and an electric field without increasing the optical propagation loss (see Figure 25a), resulting in efficient control of coupled light in the waveguides [537]. Ferroelectric thin-film channel waveguides are also employed for switches. 

A large amount of researches have carried out on designing of switching networks and subsystems based on high-speed and low-loss PLZT switches, resulting in many effective (low crosstalk and a small switching bias) architecture being proposed and switching chips being fabricated (some examples have been shown in Figure 25b–f), such as 1 × N switches which have a simple tree structure so that the introduced optical signal is delivered to one of N output ports, and N × N switches which are tree-type double-switch architectures using a strictly non-blocking tree structure composed of dc-switch elements *etc.* [537,538]. Additionally, an optical switch in simple waveguide geometry with a metal thin film sandwiched between two layers of electrooptic materials (BST) has been demonstrated with the advantages of very low crosstalk (−27 dB) and high efficiency [539]. For more intensive knowledge, some papers on optical switching technologies are available [540,541,542].

To conclude, epitaxial deposition techniques have yielded highly transparent ferroelectric thin films with low loss, which covers a wide spectral region that are suitable for high performance optical waveguide devices such as electrooptic modulators and switches, promising in practical use. Furthermore, their potential incorporation with other devices in multifunctional optical integrated circuits can result in producing of integrated optical devices that can modulate, switch, and amplify light potentially feasible all within the same platform, attractive for future applications.

### 5.5. Tunable Microwave Devices

Tunable microwave devices, such as tunable oscillators, phase shifters and varactors, *etc.*, have attracted great attention for their wide applications in field of communication. Ferroelectric materials, which have strong electric field dependence in dielectric permittivity and the relatively low dielectric loss at microwave frequency, are considered as the most promising candidates for microwave devices since 1960s [543]. Among them, ferroelectric thin films with low tuning voltages and relatively low production costs are of considerate interests, and have inspired wide investigations since 1980s, corresponding to the trend of miniaturization [544]. Far different from those components such as SAW introduced in the former text which based on piezoelectric effect, tunable microwave devices are on the basis of permittivity tenability. Excellent reviews covering different aspects of tunable ferroelectric materials and their applications for tunable microwave device can be found in the open literature [544,545,546,547].

For practical use, some factors including high dielectric tunability, low dielectric loss tangent, appropriate level of dielectric constant, appropriate working frequency, and fast tuning response are basic requirements for tunable microwave devices. Generally, ferroelectric hysteresis is unwanted in tunable microwave applications for the reason that it causes ambiguity in the applied voltages required for reaching a certain value of the capacitance, attributed to the complex domain evolution process at ferroelectric phase. The paraelectric phase has lower dielectric loss due to the disappearance of hysteresis, thus ferroelectrics with a Curie temperature below operating temperature are usually used in practical device designs. So far, Ba_1−x_Sr_x_TiO_3_ (BST) thin films are the most widely studied ones with high performance in microwave tuning. Reviews on BST-based device fabrication processes, influence factors, application states, *etc.*, are available [545]. In order to improve the quality of tunable ferroelectric thin films, many factors must be taken into account mainly including stoichiometry and substrate misfit strain which may cause variation and shifts of the Curie temperature, dead layers (dielectric layers near the interface) which may lead to reduction of the permittivity, as well as microstructure which may induce dielectric loss. Large investigations have been carried out on quality improvement of BST thin films, among which Mg-based doping [547,548] or Mn-doping [549] has been demonstrated as an effective way. Alternatively, many competitive materials in thin film form have also been reported, such as STO (which is used for devices operating at low temperatures (~77 K)) [550,551,552], Pb_x_Sr_1−x_TiO_3_ [553], Bi_1.5_Zn_1.0_Nb_1.5_O_7_ (BZN) [554,555], (Pb,Ba)ZrO_3_ [556], and some BST-related ones like (Ba,Sr)(Zr,Ti)O_3_ (BSZT) [557], *etc.* Differently, some tunable devices with ferroelectrics in the ferroelectric phase have been demonstrated, such as Na_0.5_K_0.5_NbO_3_ thin films [558].

Large amounts of microwave devices integrating STO and BST thin films have been demonstrated in the past 20 years [551,558,559]. Varactor is the simplest tunable passive component based on ferroelectric thin films, which is widely used in microwave technology for analog (non-digital) tuning purposes. Ferroelectric varactors with better Q factor and tunability than semiconductor analogs in the frequency range above 10–20 GHz are preferred, which usually fabricated in two basis structures including sandwich structure and planar structure [544]. The former one offers smaller tuning voltages but limits high frequency applications, while the latter offers smaller capacitance and the possibility of trading tunability for lower losses but higher tuning voltages. The tunable microwave ferroelectric phase shifter is another widely used device playing important role in phased array antennas, which often based on varactors. Integration of ferroelectric thin films in phase shifters with appropriate microwave circuits have substantially reduced the size, mass, and cost of the antennas. Commonly, a phase shifter component simply contains a transmission line patterned on a ferroelectric thin film (usually a BST thin film) with appropriate length or a transmission line periodically loaded with sandwich-structured varactors. Some reported works on phase shifter devices are available [560,561]. Besides these two kinds of widely used components, ferroelectric thin films also have gained ground for other devices such as delay lines, tunable filters, and microwave switches, *etc*. 

To acquire better performance at room temperature, tunable microwave devices which are on the basis of ferroelectric thin films still need adequate treatment. Incorporation of ferroelectric thin films like STO (for low temperature use) and BST (for room-temperature use) with high temperature superconductors such as YBa_2_Cu_3_O_7-δ_ (YBCO) can be an effective way for the improvement in loss performance [545]. Heterostructures such as BZN/BST [562], BST/ZrO_2_ [563], Mn-doped BST/BZT [564], *etc.*, have been reported to possess excellent dielectric properties stable over a broad temperature range, promising for high-performance room-temperature tunable microwave devices. In particular, Lee *et al.* [565] have created a new kind of nanoscaffold composite ferroelectric material containing BST and Sm_2_O_3_ with a very high tunability which scales inversely with loss, indicating great potential in practical use.

### 5.6. Applications Based on Pyroelectric and Electrocaloric Effects

Pyroelectric effect of ferroelectric thin films (e.g., BST, Mn-doped PZT, PLZT, (Na, Bi)TiO_3_) has been exploited to produce room-temperature sensitive detectors of modulated infrared radiation, and have been applied to non-cryogenic, low-cost, compact-structured and high-performance thermal imaging systems [566,567,568,569]. Advanced thin film depositing methods have allowed the construction of better thermally isolated infrared sensor structures with enormous progresses being made, which have been reviewed by several researchers [496,570]. In contrast, depending on electrocaloric effects (the converse pyroelectric effect), an applied electric field can reversibly change the temperature of an electrocaloric material under adiabatic conditions, which is strongest near phase transitions. Giant electrocaloric effects have also been demonstrated in ferroelectric thin films near their ferroelectric Curie temperature, such as PZT [571] and PMN-PTO [572] reported by Mischenko *et al.*, which will widen the potential for applications in cooling systems such as providing cooling solutions for electronic components, recover useful electrical power from waste heat, *etc.*

### 5.7. Prospective Applications of Ultrathin Ferroelectric Films

Besides the above mentioned applications, it is promising to utilize the unique properties of ultrathin ferroelectric films, such as quantum transport properties, abnormal electromechanical behaviors, and giant flexoelectricity, *etc.*, to develop novel and high performance devices. Recent progresses achieved in growth and characterization techniques have triggered intense application researches on newly discovered effects and phenomena in ultrathin ferroelectric films.

**Figure 26:**
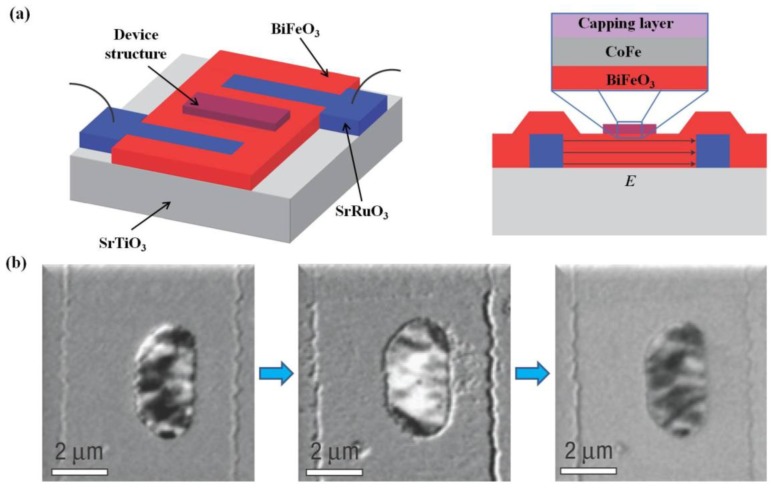
(**a**) Three-dimensional (left) and cross-sectional (right) schematic diagrams of the coplanar epitaxial electrode device showing the structure that will enable controlled ferroelectric switching and electrical control of local ferromagnetism in the CoFe features [576]; (**b**) XMCD–PEEM images taken at the Co L-edge revealing the ferromagnetic domain structure of the CoFe features in such a coplanar electrode device structure in the as-grown state (left), after the first electrical switch (middle) and after the second electrical switch (right) [576].

Since coexistence and cross-coupling of several ferroic orders such as ferromagnetic and ferroelectric have been observed in multiferroic materials, it brings about novel physical phenomena and offers possibilities for new device functions, which is of increasing interests for memory and logic device applications as the coupling enables the dynamic interaction between these order parameters. More significant, the concomitant observation of ferromagnetism and ferroelectricity in epitaxial thin films have also been reported with remarkable retaining of the multiferroic character in thin-film form down to a thickness of several nanometer magnitude [573]. Multiferroic ultrathin films offer the opportunity of combining the respective advantages of fast low-power electrical write operation and non-destructive magnetic read operation in FeRAMs and MRAMs (magnetic random access memories), resulting in the realization of four-state logic or even more state logic in an integrated single device [573,574], as well as the exploiting of magnetoelectric random access memories (MERAMs) which combines the magnetoelectric coupling with the interfacial exchange coupling between a multiferroic and a ferromagnet to switch the magnetization of the ferromagnetic layer by using a voltage [575]. Ultrathin BFO film, which is an antiferromagnetic, ferroelectric multiferroic with a Curie temperature of ~820 °C and a Neel temperature of ~370 °C, has been reported as a good choice for offering opportunity to gain electrical control of exchange interactions in MERAM devices by Chu *et al.*, with the obvious controlling result revealing an essential step towards the fabrication of prototype MERAM elements that can be reproducibly switched by an electric field (device structure have been shown in Figure 26a, with controlling evidence of XMCD–PEEM images shown in Figure 26b) [576].

**Figure 27:**
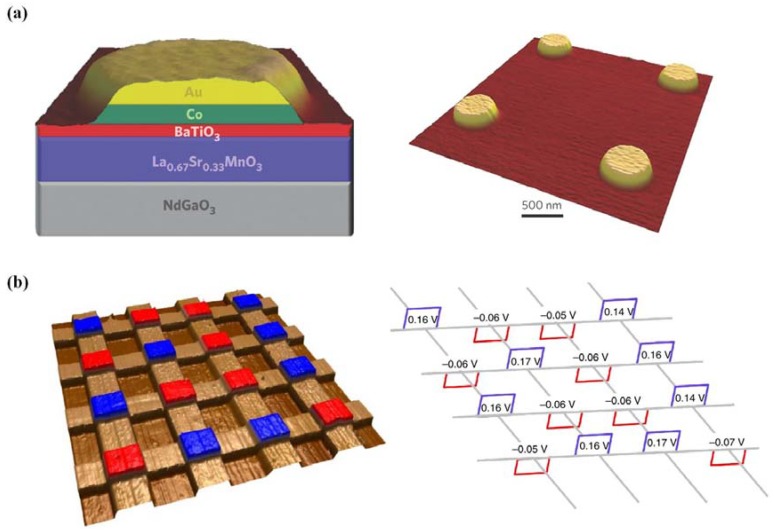
(**a**) Schematic of one gold/cobalt/BTO/LSMO nanodevice on a NGO substrate (left), and AFM image of four typical nanodevices defined using electron-beam lithography (right) [385]; (**b**) a prototype 16-cell memory based on the cross-bar architecture: topography of the device with preset polarization direction where blue ones refer to polarization up and red ones refer to polarization down (left), *Voc* of all 16 cells, measured under 20 mW·cm^−2^ light (right) [578].

**Figure 28:**
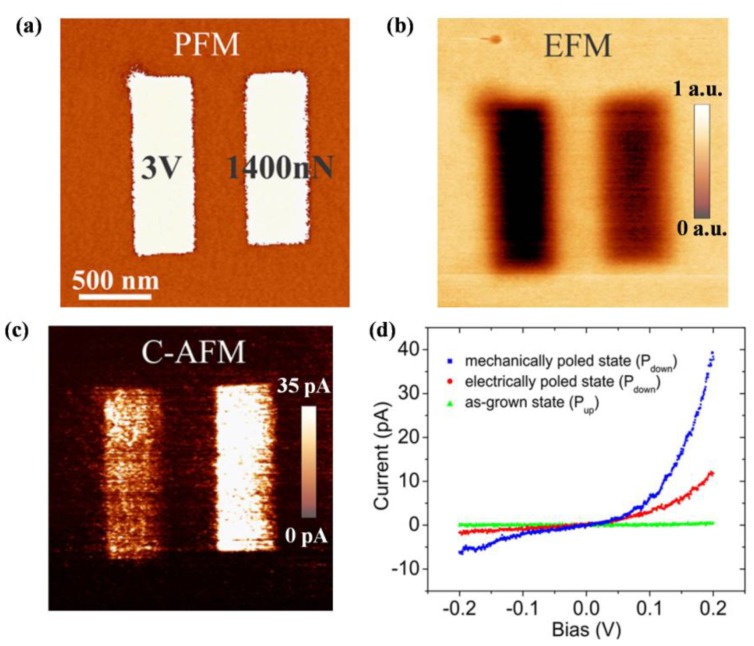
(**a**) PFM phase images of the 4.8-nm-thick BaTiO_3_ film after the generation of the downward-oriented domains by electrical poling (left) and mechanical loading force (right); (**b**) EFM image taken at the same location as the PFM images, which is acquired with a dc read voltage of +2 V; (**c**) tunneling current map acquired in the c-AFM mode with a dc read bias 0.3 V at the same location as the PFM and EFM images, where the bright contrast corresponds to a higher conductivity; (**d**) local I−V curves measured for the as-grown film area with upward polarization (green), electrically downward-switched domain (red), and mechanically downward-switched domain (blue), which reveals that mechanically induced TER is larger than the electrically induced TER [348].

In a ferroelectric tunnel junction (FTJ), coupling of ferroelectric or multiferroic and quantum-mechanical tunneling in the FTJ with ultrathin barrier layers can allow a simple and fast readout of the stored information through the influence of ferroic orders on the tunnel current, which may well meet the current quest for a solid state universal memory with high-storage density, high read/write speed, random access and non-volatility. Because of this attractive concept, recent researches have been focusing on nonvolatile memory devices depend on FTJs, especially those with a multiferroic barrier layer. As what have been significantly reported by Gajek *et al.* [573], La_0.1_Bi_0.9_MnO_3_ (LBMO) thin films retain ferromagnetic and ferroelectric properties down to a thickness of 2 nm. Spin-filter-type tunnel junctions with integrated LBMO ultrathin films as tunnel barriers can operate as a four-resistance-state system, which is related to interplay between ferroelectricity and spin-dependent tunneling, suggesting the possibility to encode quaternary information and to read out it non-destructively by a resistance measurement. Based on this demonstration of a four-state resistive memory element in a tunnel junction, Scott [577] has pointed out that multiferroics might hold the future for the ultimate memory device, which is effective at a 2 V bias operation, compatible with the standard silicon-chip 5V-logic level in practical use. Meanwhile, since the device analyzed would require liquid nitrogen cooling, the search for a room-temperature ferroelectric ferromagnetic for practical use remains, calling for more intensive works. More recently, Chanthbouala *et al.* [385] have reported another nonvolatile memory with OFF/ON ratios as high as 100 and write powers as low as ~1 × 10^4^ A·cm^−2^ at room temperature in a BTO(2 nm)/LSMO(30 nm) heterostructure, which has been schematically shown in Figure 27a. The FTJs in their work show large, stable, reproducible and reliable tunnel electroresistance with resistance switching occurring at the coercive voltage of ferroelectric switching, which store data in the electric polarization direction of a BTO ultrathin film tunnel barrier and have the advantage of being based on a purely electronic mechanism instead of voltage-induced migration of matter at nanoscale, emerging as a high performance alternative to other currently used resistive memories. It also can be inferred that FTJ coupling with magnetic electrodes has the potential to yield low-power electric-field-controllable spintronics device operation at room temperature. Moreover, strain-mediated voltage-free generation of a stable, fully reversible and reproducible tunneling electroresistance effects (TER) have also been experimentally reported in a FTJ with a single-crystalline BaTiO_3_ barrier of 4.8 nm thick (see Figure 28), which is facilitated by flexoelectric switching of polarization due to the strain gradient developed under the SPM tip pressed against the film surface, opening the possibility for application in low-energy archive data storage devices with mechanical writing and electrical reading of data [348].

Hereto, it is certain that the resistance change of a FTJ upon polarization reversal can be used to sense the polarization direction non-destructively. Thus it is reasonable to deduce that ultrathin ferroelectric films, preferably multiferroic films, integrated in FTJs should be the most promising candidates for next-generation nonvolatile memory devices. Meanwhile, FTJ devices have limitation in critical thickness of ultrathin ferroelectric tunnel barriers, which is vital to the properties and performance of this kind of memories. Furthermore, the requirements for the ferroelectric layer to be at most several nanometers thick should pose a tremendous challenge on device fabrication in practical applications. For practical use, more works should be going on.

Another appealing application in nonvolatile memory devices should attribute to intensive photovoltaic effect of ultrathin ferroelectric films. As both signs of open circuit voltage and short circuit photocurrent dependent on the polarization direction [579], they can serve as the read-out signal in a memory device [580]. Accompany with the film thickness decreasing, polarization switching will become more and more sensitive, thereby resulting in ultrathin-film based nonvolatile memory devices which present higher read/write speed. Also, the multiferroic ultrathin film of BFO, which has a band gap (~2.7 eV) within visible light range, is considered as a candidate most likely offering a unique opportunity for memory application, which has been emphasized recently by Guo *et al.* [578], who have proposed a novel approach to create a nonvolatile memory technology that uses the polarization-dependent photovoltaic effect in ferroelectrics. They demonstrated that epitaxial ultrathin BFO films can be used to sense the polarization direction non-destructively in a ferroelectric memory, with the writing time can be as short as 10 ns. Except for the favorable data retention, fatigue performance and energy consumption, a prepared prototype 16-cell memory based on the cross-bar architecture (shown in Figure 27b) has also demonstrated the feasibility of this technique, which compares favorably with other nonvolatile memories that are under development in current such as FeRAMs, MRAMs, and RRAMs. Since film thickness and electrode materials have important effects, further optimization is needed for better devices performance. Electrodes with larger screening length are preferred considering the increasing depolarization field should enhance the open circuit voltage. Of course, the concept is not limited to BFO, other ferroelectric materials such as PZT and BTO may give rise to similar behavior.

Furthermore, Yang *et al.* [378] have reported that electric-field control over domain structure can allow the photovoltaic effect to be reversed in polarity or turned off in BFO thin films. This new degree of control, together with the high voltages produced, may find application in optoelectronic devices. Since domain wall density scales inversely with film thickness, photovoltage is larger in thinner films, which implied application of ultrathin films in optoelectronic devices better than the thicker ones. Additionally, according to what have been mentioned in Section 4, ferroelectric films will be more sensitive to external mechanical load or strains arising from electrodes and substrates as the deceasing thickness, thus may give rise to more giant piezoelectric effect or contrarily electrostriction effect and even flexoelectric effect in ultrathin ferroelectric films (without crack limits in thicker films), which should to a large extent improve performance and function of piezo-based integrated MEMS, promising for the next generation of nano-electromechanical systems (NEMS). Moreover, it is more probably to realize single-domain in epitaxial ferroelectric ultrathin films with small surface roughness, which is desired in nonlinear optical waveguide devices because of their little optical scattering.

To conclude, due to their smaller film thickness and special properties, ferroelectric ultrathin films meet the minimization and integration trend of modern device technology and have great potential in effectively high-performance device applications including memory devices, integrated MEMS, as well as optoelectronic devices, *etc*. Their rapid developments have dramatically changed the current device concepts and present a comprehensive group of high-speed, high density, low-consumption, and multifunctional devices in new and upcoming application markets. Moreover, applications of ferroelectric ultrathin films are more extensive than what have been reviewed in this paper. Other potential applications are in need of exploiting and intensive researches are required for further optimization in this field, such as practical fabrication technology for ultrathin film devices and more optimized device design, *etc*.

## 6. Outlook and Summary

In this review, we have attempted to give a comprehensive overview on state-of-the-art experimental researches on the field of ultrathin ferroelectric films (typically, with thickness less than 100 nm), seeking to cover the important advances in growth methods, characterization techniques, phenomena and physics, as well as device applications of ultrathin ferroelectric films in the past a little more than a decade. Important phenomena and properties in those ultrathin ferroelectric films with film thickness done to several nanometers have been emphasized, and prospective applications of this kind of films have been highlighted.

Besides being the promising candidate for developing the next generation of nonvolatile memory, ferroelectric ultrathin films become more and more important in other device applications due to the increasingly reported properties. Notably, depending on those robust experimental results of the novel electron transport properties of ferroelectric ultrathin films, e.g., the giant electroresistance effect in ferroelectric tunnel junctions (FTJs), and the domain or domain wall mediated electron transport behaviors in ferroelectric ultrathin films, they should be of great importance in the field of next-generation electronic devices. Particularly, based on the interplay of these properties with external thermal, electrical and mechanical fields, the development of high performance sensors and transducers is promising.

At present, researches of ultrathin ferroelectric films are largely to be application driven. Although there are already many matured applications based on ferroelectric thin films, more intensive works are still in need to exploit potential applications of more thinner films and more promising mechanisms, which stimulates numerous researches to optimize film fabrication and characterization techniques for further improvement of film properties. Some important issues, such as ferroelectric size effects, strain engineering, interface electrostatics, topological defects in domain structure, and coupling effects of different order parameters in multiferroic systems, *etc.*, are yet open. Moreover, new materials as well as new structures need to be further investigated. Future experimental developments in ultrathin ferroelectric films should not only discover novel phenomena and properties but also bring remarkably broader applications.
